# Design and synthesis of naphthalimide group-bearing thioglycosides as novel β-*N*-acetylhexosaminidases inhibitors

**DOI:** 10.1080/14756366.2017.1419217

**Published:** 2018-02-02

**Authors:** Shengqiang Shen, Wei Chen, Lili Dong, Qing Yang, Huizhe Lu, Jianjun Zhang

**Affiliations:** a Department of Applied Chemistry, College of Science, China Agricultural University, Beijing, China;; b School of Life Science and Biotechnology, Dalian University of Technology, Dalian, China

**Keywords:** Thioglycosides, naphthalimide derivatives, *β-N*-acetylhexosaminidase, *O*-GlcNAcase, inhibitors

## Abstract

GH20 human β-*N*-acetylhexosaminidases (hsHex) and GH84 human *O*-GlcNAcase (hOGA) are involved in numerous pathological processes and emerged as promising targets for drug discovery. Based on the catalytic mechanism and structure of the catalytic domains of these β-*N*-acetylhexosaminidases, a series of novel naphthalimide moiety-bearing thioglycosides with different flexible linkers were designed, and their inhibitory potency against hsHexB and hOGA was evaluated. The strongest potency was found for compound **15j** (*K*_i_ = 0.91 µM against hsHexB; *K*_i_ > 100 µM against hOGA) and compound **15b** (*K*_i_ = 3.76 µM against hOGA; *K*_i_ = 30.42 µM against hsHexB), which also exhibited significant selectivity between these two enzymes. Besides, inhibitors **15j** and **15b** exhibited an inverse binding patterns in docking studies. The determined structure–activity relationship as well as the established binding models provide the direction for further structure optimizations and the development of specific β-*N*-acetylhexosaminidase inhibitors.

## 1. Introduction

β-*N*-acetylhexosaminidases (EC 3.2.1.52) are widely distributed exo-glycosidases that catalyze the release of a β-linked *N*-acetyl-d-hexosamine unit from non-reducing ends of glycoconjugates. These enzymes are classified into three glycosyl hydrolase family (GH3, GH20 and GH84) based on amino acid sequence similarities in the CAZy classification system (http://www.cazy.org)^1^^,^^2^. And they are involved in various physiological functions, such as energy metabolism^3^, cell communication^4^, cell proliferation^5^ and inflammation^6^. Among these, the GH20 human β-*N*-acetylhexosaminidases (hsHex) and GH84 human *O*-GlcNAcase (hOGA) are the most promising targets for drug development.

GH20 human β-*N*-acetylhexosaminidases (hsHex) have gained many attentions owing to their pivotal actions in osteoarthritis and lysosomal storage disorders. Research has shown that hexosaminidase is a dominant enzyme released into the extracellular compartment by chondrocytes in patients with osteoarthritis. Moreover, inhibition of the enzyme can prevent cartilage matrix degradation, providing a new avenue for treatments of osteoarthritis^6^^,^^7^. Likewise, lysosomal hexosaminidase can degrade GM2 gangliosides in neuronal cells. Dysfunction of this enzyme not only causes severe neurodegenerative lipid storage disorders, but also leads to Tay-Sachs or Sandhoff disease^8^. Inhibitors of lysosomal hsHex can therefore be used as pharmacological chaperones to increase activity of mutant lysosomal enzyme while maintain its metabolic function at a normal level^9^^,^^10^.

GH84 human *O*-GlcNAcase (hOGA) catalyzes the removal of *O*-GlcNAc from serine or threonine residues in glycoproteins, and has been found to link to Alzheimer’s disease (AD)^11^. Evidences have shown that AD is closely associated with tau hyperphosphorylation in patient’s brain, while such site remains protected by *O*-GlcNAc in a healthy neuron^12^. Thus, human *O*-GlcNAcase inhibitors that block tau hyperphosphorylation can help in the treatments of AD^13^^,^^14^.

A number of small molecule inhibitors against β-*N*-acetylhexosaminidases have been reported. These include PUGNAc^15^ (**1**), Nagstatin^16^ (**2**), NAG-thiazoline^17^ (**3**), pyrimethamine^18^ (**4**), iminocyclitols^19^^,^^20^, and naphthalimides^21–24^. Among these, PUGNAc, Nagstatin, and NAG-thiazoline are the three classic potent inhibitors, which however show non-selectivity between GH20 and GH84 β-*N*-acetylhexosaminidases^4^^,^^25^. Pyrimethamine has been approved by FDA to be a safe and a suitable pharmacological chaperone of human lysosomal hsHex A^18^. Although the well-studied inhibitors, iminocyclitols, always become handicapped in complex synthetic methods^19^^,^^20^. It is worth mentioning that Ho has reported a series of iminocyclitol derivatives, in which alkylamine chains were attached to the iminocyclitol ring^26^. The most potent compound **4** (**5**), containing two methoxyphenyl groups, has a *K*_i_ value of 0.69 nM against hsHexB and exhibits 250,000-fold higher selectivity toward GH84 hOGA^26^. Further docking analysis has revealed that a hydrophobic cleft located near −1 subsite (active pocket) and alkylamine chains that extend out into the cleft can acquire additional interactions^26^. Naphthalimides are β-*N*-acetylhexosaminidases inhibitors discovered during a high-throughput screening. One of these inhibitors, **M-31850** (**6**) exhibits IC_50_ values of 6.0 µM and 3.1 µM against hsHexA and hsHexB, respectively^21^. Following this discovery, a large number of non-carbohydrate-based naphthalimide derivatives are synthesized and evaluated as β-N-acetylhexosaminidases inhibitors by Qian and Yang^22–24^. An example of these is compound **7a** (**7**), which exhibits high inhibitory potency with a *K*_i_ value of 0.63 µM against hsHex, 3.3 folds lower than that of **M-31850**^22^. In addition, compound **Q1** (**8**) shows inhibitory potency against GH20 insect β-*N*-acetylhexosaminidase OfHex1 with *K*_i_ values of 4.28 µM. The complex crystal structure of OfHex1**-Q1** (PDB ID: 3WMB) reveals that the methylthiadiazole group of **Q1** binds the −1 subsite of OfHex1, whereas the napthalimide group is sandwiched by the amino acid residues of the +1 subsite (outside of −1 subsite)^23^.

**Figure 1.**
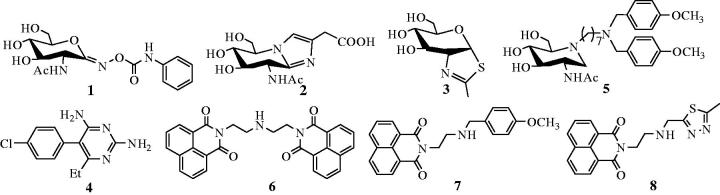
Reported β-*N*-acetylhexosaminidases inhibitors.

**Figure 2.**
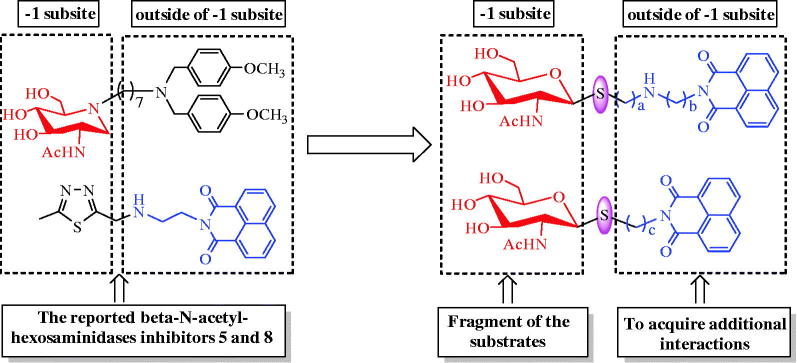
Design of thioglycoside inhibitors.

These observations prompted us to choose *N*-acetyl-d-glucamine as the structural fragment to provide strong affinity toward −1 subsite of β-*N*-acetylhexosaminidases and convert the glycosidic bond to thioglycosidic bond to prevent hydrolysis by the enzymes. Then, the napthalimide groups with different linkers were further introduced into the structure to acquire additional interactions outside of the −1 binding subsite. Influences of length and nitrogen atom of the linkers between *N*-acetyl-d-glucamine and napthalimide group could also be compared. Thus, in this work, we synthesized a series of naphthalimide-bearing thioglycoside derivatives. Their inhibitory potency was evaluated *in vitro* against two enzymes, GH20 human hsHexB and GH84 hOGA. The kinetic and molecular docking studies were carried out to further explore their interaction mechanisms with hsHexB and hOGA (Figures 1 and 2).

## 2. Experimental

### 2.1. Materials

All chemicals, reagents, and solvents were purchased from commercial sources. The solvents were dried prior to use. Reaction progress was monitored using thin layer chromatography (TLC) on pre-coated silica gel GF254 plates, in which the spots were detected by charring with 30% (v/v) H_2_SO_4_ in MeOH or by UV light (254 nm). Melting points were determined using a Ry-1 G melting point instrument. ^1^H NMR and ^13 ^C NMR spectra were carried out on a Bruker AVANCE600 spectrometer in CDCl_3_ or DMSO*-*d_6_ at 25 °C and referenced to TMS. High-resolution mass spectra (HRMS) were collected by the Beijing Launcher Scientific Co. Ltd.

### 2.2. Chemical synthesis

#### 2.2.1. Synthesis of compounds 10, 13a–13c, 19a–19e

Thiol **10** was prepared according to procedures described in the literature^27^^,^^28^. Compounds **13a–13c** were synthesized from 1,8-naphthalic anhydride as described previously^29^. Compounds **19a–19e** were prepared from 1,8-naphthalimide according to previous methods^30^. Data for compounds **10, 13a–13c** and **19a–19e** can be found in Supporting Information.

#### 2.2.2. Synthesis of compounds 11a–11d and 16

Following thiol **10** (5.5 mmol, 1.0 eq) was dissolved in acetone (20 ml) and H_2_O (10 ml), solid potassium carbonate (6.6 mmol, 1.2 eq) and α,ω-dibromoalkane (44 mmol, 8.0 eq) were added. The mixture was stirred for 20 h at room temperature and subsequently concentrated *in vacuo*. The resulting residue was diluted with DCM (50 ml), washed with H_2_O (100 ml), brine (100 ml), dried over Na_2_SO_4_, and concentrated *in vacuo*. Finally, the residue was subjected to chromatography, from which the compounds **11a–11d** and **16** were obtained.

Data for compounds **11a–11d** and **16** can be found in Supporting Information.

#### 2.2.3 Synthesis of compound 11e

Thiol **10** (1.0 g, 2.8 mmol) was first dissolved in 1,4-dibromobutane (10 ml, 83.7 mmol) and DBU (0.5 ml, 3.4 mmol) was then added. The reaction mixture was stirred at room temperature for 3 h, until TLC (EtOAc) indicated all starting materials were consumed and formed the final product. Following phase separation, the organic layer was diluted with DCM (60 ml), washed with H_2_O (100 ml), brine (100 ml), dried over Na_2_SO_4_, and concentrated *in vacuo*. In the final step, the residue was purified by column chromatography to yield **11e** as a white solid. (0.65 g, 46.7%)yield; [α]_D_^25^–78.8 (c = 0.50, CHCl_3_); mp152–154 °C; ^1^H NMR (300 MHz, CDCl_3_) δ 5.73 (d, *J* = 9.4 Hz, 1H, NH), 5.17 (t, *J* = 9.7 Hz, 1H, H-3), 5.08 (t, *J* = 9.6 Hz, 1H, H-4), 4.59 (d, *J* = 10.3 Hz, 1H, H-1), 4.28–4.04 (m, 3H, H-6a, H-6 b, H-2), 3.70 (ddd, *J* = 9.5, 4.8, 2.3 Hz, 1H, H-5), 3.43 (t, *J* = 6.6 Hz, 2H, CH_2_Br), 2.85–2.61 (m, 2H, SCH_2_), 2.08, 2.03, 2.02 (3 s, 9H, 3 OAc), 1.98 (s, 3H, NAc), 1.98–1.91 (m, 2H, CH_2_), 1.83–1.71 (m, 2H, CH_2_); HRMS (ESI) calcd for C_18_H_29_BrNO_8_S (M + H^+^) 498.0797, found 498.0791.

#### 2.2.4. Synthesis of compounds 14a–14l

A mixture of compounds **11a–11d** (2 mmol, 1 eq), compounds **13a–13c** (3 mmol, 1.5 eq) and potassium carbonate (2.4 mmol, 1.2 eq) in acetonitrile (50 ml) was refluxed for 4 h, until TLC (EtOAc/MeOH  = 6/1) indicated that the reaction was complete. After the undissolved solid was removed by filtration, the filtrate was concentrated *in vacuo*. The resulting solid residue was further purified by silica gel column chromatography using EtOAc/CH_3_OH (12:1), in which compounds **14a–14l** were obtained.

Data for compounds **14a–14l** can be found in Supporting Information.

#### 2.2.5. Synthesis of compounds 15a–15l

After compounds **14a–14l** solution (1 mmol) was mixed with MeOH (15 ml), saturated solution of NH_3_ in MeOH (6 ml) was added. The reaction mixture was stirred for 40 h at room temperature, and until TLC (EtOAc:MeOH:H_2_O = 8:1:1) indicated that the reaction was completed. The mixture was then concentrated *in vacuo*, purified by flash column chromatography (EtOAc:MeOH  = 6:1) to obtain compounds ***15a–15l***.

*2-[2-[2-[(2-acetamido-β-d-glucopyranosyl)thio]ethylamino]ethyl]-1H-benzo[de] isoquinoline-1,3(2H)-dione (****15a****)*

White solid; (0.46 g, 92.0%) yield; [α]_D_^25^–10.7 (c = 0.10, DMF); mp 223–225 °C; ^1^H NMR (300 MHz, DMSO-d_6_) δ 8.53–8.40 (m, 4H, ArH), 7.91–7.81 (m, 2H, ArH), 7.71 (d, *J* = 9.3 Hz, 1H, NHAc), 5.01 (d, *J* = 4.7 Hz, 1H, OH), 4.96 (d, *J* = 5.3 Hz, 1H, OH), 4.49 (br s, 1H, OH), 4.36 (d, *J* = 10.3 Hz, 1H, H-1), 4.17–4.08 (m, 2H, H-3, H-4), 3.66 (dd, *J* = 11.4, 3.4 Hz, 1H, H-6 b), 3.57–3.38 (m, 2H, H-2, H-6a), 3.31–3.20 (m, 1H, H-5), 3.15–3.05 (m, 2H, CH_2_NC=O), 2.85–2.72 (m, 4H, 2 CH_2_), 2.72–2.54 (m, 2H, SCH_2_), 1.79 (s, 3H, NAc); ^13 ^C NMR (75 MHz, DMSO-d_6_) δ 169.05, 163.64, 134.36, 131.41, 130.82, 127.53, 127.32, 122.26, 84.37, 81.26, 75.71, 70.63, 61.35, 54.73, 48.90, 46.39, 39.57, 29.83, 23.17; HRMS (ESI) calcd for C_24_H_30_N_3_O_7_S (M + H^+^) 504.1804, found 504.1805.

Data for compounds **15b–15l** can be found in Supporting Information.

#### 2.2.6. Synthesis of compound 17

Compound **16** (0.78 g, 1 mmol) was first suspended in MeOH (15 ml), and saturated solution of NH_3_ in MeOH (10 ml) was then added. The reaction mixture was stirred for 50 h at room temperature, until TLC (EtOAc:MeOH:H_2_O = 8:3:1) indicated that the reaction was complete. The mixture was further concentrated *in vacuo* and recrystallized from MeOH/ether, which resulted in compound **17** (0.43 g, 81.3%) as a white solid. [α]_D_^25^–33.4(c = 0.90, DMF); mp 236–238 °C; ^1^H NMR (300 MHz, DMSO-d_6_) δ 7.70 (d, *J* = 9.2 Hz, 2H, 2 NHAc), 4.99 (d, *J* = 4.1 Hz, 2H, 2 OH), 4.95 (d, *J* = 5.3 Hz, 2H, 2 OH), 4.51 (t, *J* = 5.6 Hz, 2H, 2 OH), 4.32 (d, *J* = 10.3 Hz, 2H, 2 H-1), 3.67 (dd, *J* = 11.4, 5.5 Hz, 2H, 2 H-6 b), 3.56–3.38 (m, 4H, 2 H-2, 2 H-6a), 3.31–3.20 (m, 2H, 2 H-5), 3.13–3.01 (m, 4H, 2 H-3, 2 H-4), 2.69–2.53 (m, 4H, 2 CH_2_), 1.79 (s, 6H, 2 NAc), 1.62–1.49 (m, 4H, 2 CH_2_). ^13 ^C NMR (75 MHz, DMSO-d_6_) δ 169.12, 84.13, 81.28, 75.64, 70.67, 61.35, 54.71, 28.74, 28.36, 23.19; HRMS (ESI) calcd for C_20_H_37_N_2_O_10_S_2_ (M + H^+^) 529.1890, found 529.1895.

#### 2.2.7. Synthesis of compounds 20a–20e

After thiol **10** (2.8 mmol, 1.0 eq) and *N*-(ω-bromoalkyl)-1,8-naphthalimides **19a–19e** (2.8 mmol, 1.0 eq) were dissolved in acetone (20 ml), potassium carbonate (3.4 mmol, 1.2 eq) and H_2_O (10 ml) were added. The mixture was stirred for 20 h at room temperature, until TLC (EtOAc) showed that the reaction was completed. The mixture was then concentrated *in vacuo*; and the resulting residue was diluted with DCM (40 ml), washed with H_2_O (80 ml), brine (80 ml), dried over Na_2_SO_4_, and concentrated *in vacuo*. Chromatography was carried out to purify the resulting compounds **20a–20e**.

Data for compounds **20a–20e** can be found in Supporting Information.

#### 2.2.8. Synthesis of compounds 21a–21e

Compounds **21a–21e** were synthesized by deacetylation of compounds **20a–20e** (1 mmol) using the procedure described for the synthesis of compound **17**.

*2-[2-[(2-acetamido-β-d-glucopyranosyl)thio]ethyl]-1H-benzo[de]isoquinoline-1,3(2H)-dione (****21a****)*

White solid; (0.42 g, 90.9%) yield; [α]_D_^25^ –13.5(c = 0.2, DMF); mp170–172 °C;^1^H NMR (300 MHz, DMSO-d_6_) δ 8.57–8.43 (m, 4H, ArH), 7.94–7.84 (m, 2H, ArH), 7.74 (d, *J* = 9.4 Hz, 1H, NHAc), 5.05 (d, *J* = 4.8 Hz, 1H, OH), 5.01 (d, *J* = 5.3 Hz, 1H, OH), 4.50 (d, *J* = 10.3 Hz, 1H, H-1), 4.45 (t, *J* = 6.0 Hz, 1H, OH), 4.34–4.21 (m, 2H, H-3, H-4), 3.70 (dd, *J* = 11.3, 6.0 Hz, 1H, H-6 b), 3.56 (dd, *J* = 19.5, 9.6 Hz, 1H, H-2), 3.47 (dd, *J* = 11.4, 5.6 Hz, 1H, H-6a), 3.33–3.26 (m, 1H, H-5), 3.22–3.10 (m, 2H, CH_2_N), 2.99 –2.74 (m, 2H, SCH_2_), 1.74 (s, 3H, NAc); ^13 ^C NMR (75 MHz, DMSO-d_6_) δ 169.07, 163.39, 134.51, 131.43, 130.91, 127.53, 127.33, 122.14, 84.86, 81.40, 75.62, 70.54, 61.35, 54.64, 39.98, 27.59, 23.11; HRMS (ESI) calcd for C_22_H_25_N_2_O_7_S (M + H^+^) 461.1382, found 461.1377.

Data for compounds **21b–21e** can be found in Supporting Information.

### 2.3. Enzyme preparation

Gene encoding hsHexB, which was engineered to contain a His_6_-tagged fusion protein, was cloned into pPIC9 expression vector (Invitrogen, Carlsbad, CA, USA), and subsequently transformed into *Pichia pastoris* GS115 (Invitrogen, Carlsbad, CA, USA) by electroporation. The cells were first cultured in BMGY broth (1% yeast extract, 1% glycerol, 2% peptone, 0.2% biotin, 1.34% yeast nitrogen, 0.1 M potassium phosphate, pH 6.0) at 30 °C. Methanol was added into the culture daily at a final concentration of 1% (v/v). When OD_600_ value reached 2.0 (after ∼120 h), the cells were harvested by centrifugation at 6000*g* for 10 min. While the pellet was resuspended in BMMY broth (1% yeast extract, 1% methanol, 2% peptone, 0.2% biotin, 1.34% yeast nitrogen, 0.1 M potassium phosphate, pH 6.0), the supernatant was subjected to ammonium sulfate precipitation (75% saturation) at 4 °C, in which the precipitate was further resuspended in distilled water and then desalted in buffer A (20 mM sodium phosphate, 0.5 M sodium chloride, pH 7.4). Subsequently, the resuspension solution was centrifuged at 17,000*g* for 30 min at 4 °C and passed through a 0.2-µm filter prior to loading onto a 5-ml HisTrap FF affinity column (GE Healthcare, Chicago, IL, USA), which was pre-equilibrated with buffer A. To remove nonspecific binding proteins, the column was sequentially washed with buffer A-containing 20 mM imidazole and buffer A-containing 50 mM imidazole. Finally, the target protein was eluted with buffer B (20 mM sodium phosphate, 0.5 M sodium chloride, 150 mM imidazole, pH 7.4). Purity of the protein was further analyzed by SDS–PAGE. hOGA was overexpressed in *Escherichia coli* BL21(DE3) and purified as described previously^31^.

### 2.4. Inhibitory potency assay

Potencies of hsHexB and hOGA were assayed by end-point experiment, in which 4-methylumbelliferyl *N*-acetyl-β-d-glucosaminide (4-MU*-*GlcNAc; Sigma, St. Louis, MO, USA) was used as a substrate. Various concentrations of inhibitors and substrates (50 µM, 25 µM, and 12.5 µM) were mixed with Britton-Robinson buffer (hsHexB, pH 4.0; hOGA, pH 6.0), dimethylsulfoxide at a final concentration of 2%, enzyme, and 40 µM 4-MU*-*GlcNAc in a reaction of 100-µl final volume, and incubated at 30 °C for 30 min. Then, the enzymatic reaction was terminated by the addition of 100 µl of 0.5 M sodium carbonate solution. The fluorescence of the released 4-methylumbelliferone was quantified (excitation at 366 nm, emission at 445 nm) on a Varioskan Flash microplate reader (Thermo Fisher Scientific, Waltham, MA, USA). To determine the inhibition constant (*K*_i_), the reciprocal plots of 1/velocity versus inhibitor were constructed. Data analysis was performed using Graph Pad Prism software (Graph Pad Software Inc., San Diego, CA, USA).

### 2.5. Molecular docking studies

The Sybyl Software (Version7.3; Tripos Associates, St. Louis, IL, USA, 2006) was used in molecular docking studies. The complex crystal structures of hsHexB-pyrimethamine (PDB ID: 3LMY) and hOGA-iminocyclitol-type inhibitor (PDB ID: 5M7U) were taken from Protein Data Bank (http://www.rcsb.org/pdb) and used as the starting model. Prior to docking calculations, the structures were first optimized using MMFF94 force field to gain corresponding low energy conformations. All water molecules were then removed and missing hydrogen atoms were added. After that, the ligand docking mode was performed to generate appropriate putative ligand pose, so called “protomol”, based on the Hammerhead scoring function with the molecular similarity algorithm in the active domain of the receptor^32–34^. Finally, molecular dockings between the ligands and the optimized crystal structure of hOGA or HsHexB were performed using the Surflex–Dock algorithm in the Sybyl 7.3 software.

## 3. Results and discussion

### 3.1. Chemical synthesis

#### 3.1.1. Synthesis of compounds 15a–15l

**Scheme 1.**
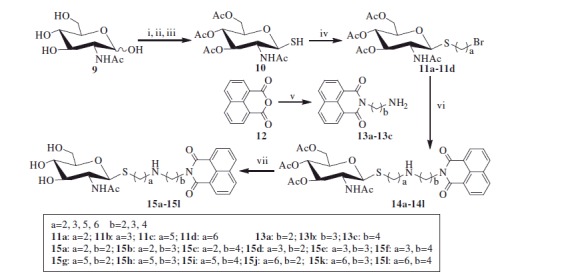
Synthesis of thioglycosides **15a–15l**. (i) AcCl; (ii) thiourea, acetone; (iii) Na_2_S_2_O_5_, DCM, H_2_O; (iv) α, ω-dibromoalkane, K_2_CO_3_, acetone, H_2_O; (v) α, ω- diaminoalkane, EtOH (vi) K_2_CO_3_, CH_3_CN; (vii) NH_3_, MeOH.

As shown in Scheme 1, the key intermediate thiol **10** was obtained from *N*-acetyl-d-glucosamine as the starting material. In the procedure, acetyl chloride was first used for acetylation and chlorination. Thiourea was then used in substitution prior to removing carbamimidoyl by Na_2_S_2_O_5_ in DCM and H_2_O. These three steps can conveniently be carried out without chromatographic purification and has made large-scale preparation of compound **10** possible. Then, compound **10** was reacted with α,ω-dibromoalkane in the presence of potassium carbonate in acetone and H_2_O to obtain mono-bromide precursors **11a–11d**. Meanwhile, 1,8-naphthalic anhydride **12** was refluxed with α,ω-diaminoalkane in ethanol to yield **13a–13c**. Subsequently, the preparations of acetyl-protected compounds **14a–14l** were completed by the reactions of bromides **11a–11d** with excess naphthalimide derivatives **13a–13c** under the condition of potassium carbonate and acetonitrile with 65–72% yield. Finally, deacetylation of hydroxyl groups by methanol-ammonia catalysis resulted in the target compounds **15a–15l**.

#### 3.1.2. Synthesis of compounds 11e and 17

**Scheme 2.**
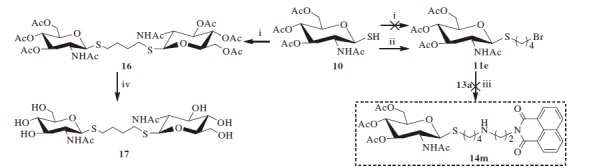
Synthesis of compounds **11e** and **17**. (i) 1,4- dibromobutane, K_2_CO_3_, acetone, H_2_O; (ii) 1,4- dibromobutane, DBU; (iii) K_2_CO_3_, CH_3_CN; (iv) NH_3_, MeOH.

Unlike the synthesis of mono-bromide precursors **11a–11d**, treatment of thiol **10** with 1,4-dibromobutane under the condition of potassium carbonate in acetone and H_2_O was not able to form the desired mono-bromide **11e**. Instead, 1,4-bisthiobutane derivatives **16** was obtained (Scheme 2). The resulting compound **16** was further deprotected by employing methanol-ammonia to gain 1,4-bis[(2-acetamido-2-deoxy-β-d-glucopyranosyl)thio] butane **17**.

In order to achieve the desired compound **11e**, 1,4-dibromobutane was selected to use as the reaction solvent and DBU was used as the acid-accepter. Under this condition, thiol **10** was successfully converted to **11e** at room temperature for 3 h with 47% yield. However, the reaction of **11e** with **13a** (in the preparation of **14a–14l)** did not yield the desired compound **14m**. Presumably, this may be because compound **11e** was not stable, and self-decomposition may take place under strong alkaline conditions (i.e. in the presence of potassium carbonate).

#### 3.1.3. Synthesis of compounds 21a–21e

**Scheme 3.**
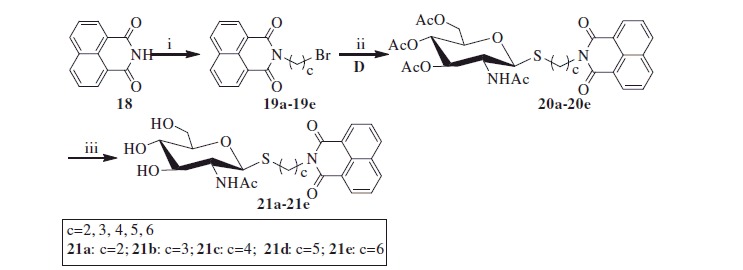
Synthesis of thioglycosides **21a–21e**. (i) α, ω-dibromoalkane, K_2_CO_3_, CH_3_CN; (ii) K_2_CO_3_, acetone, H_2_O; (iii) NH_3_, MeOH.

As shown in Scheme 3, reaction of the starting material 1,8-naphthalimide **18** with α, ω-dibromoalkane was carried out to yield bromoalkyl naphthalimides **19a–19e**. Its further reaction with thiol **10** in acetone and H_2_O resulted in acetyl-protected precursors **20a–20e** with 80–86% yield. Finally, the resulting naphthalimide derivatives **21a–21e** were deprotected by methanol-ammonia catalysis with 88–92% yield.

### 3.2. Bioevaluation of inhibitory potency

#### 3.2.1. Inhibition studies

**Figure 3.**
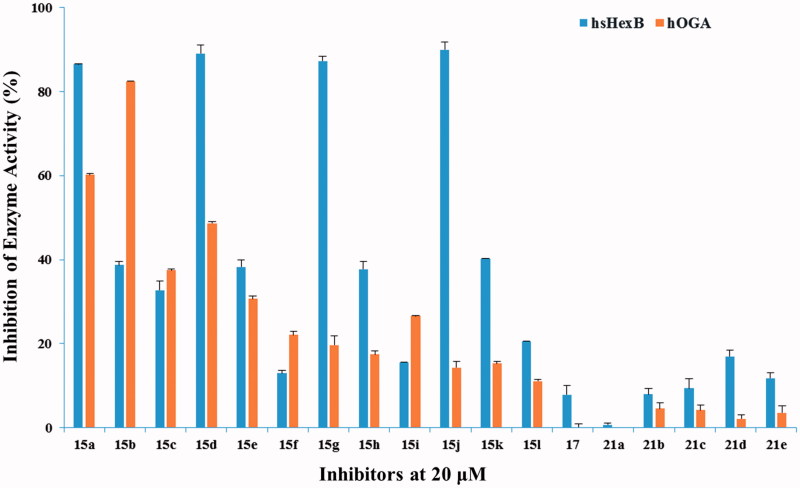
Inhibition of hsHexB in comparison to hOGA.

The target compounds **15a–15l**, **17**, and **21a–21e**, each at 20 µM, were evaluated for their inhibition potency against hsHexB and hOGA. As shown in Figure 3, compounds **21a–21e** (nitrogen atom is absent in the linker) exhibited lower inhibitory potency compared with that of compounds **15a–15l**. Compound **17** without naphthalimide moiety hardly showed activity, suggesting that naphthalimide moiety may be involved in the improved binding affinity.

Analysis of compounds **15a–15l** against hsHexB revealed a significant correlation between the efficiency of the inhibitors and the position of nitrogen atom in the linker. Specifically, compounds **15a**, **15d**, **15g**, and **15j** containing 2-aminoethyl-naphthalimide moiety exhibited the highest potencies. Increasing number of carbon atom (from 2-aminoethyl to 4-aminobutyl) brought the potency to steadily dwindle compared with compounds **15a**, **15b**, **15c**. Besides, the glycosyl moieties with different length of linkers (Scheme 1, with the value b) played partial inhibitory role against hsHexB. Further K_i_ determination showed that compound **15j** (*K*_i_ = 0.91 µM against hsHexB; *K*_i_ > 100 µM against hOGA) exhibited excellent selectivity and good inhibition potency against hsHexB.

Analysis of compounds **15a–15l** against hOGA showed that the inhibitory potency of these compounds was associated with the length of the linker between glycosyl and naphthalimide group. The linker containing five to seven atoms in compounds **15a–15e** exhibited higher inhibitory potency than those with longer chains in compounds **15f–15l**. This suggests that the existed hydrophobic domain is in the proximity of −1 binding-site pocket so that it can bind to naphthalimide group^35^. Position of nitrogen atom in the linker appears to have an effect on the efficiency of the inhibitors. For instance, although compounds **15b** and **15d** had the same length of linker, compound **15b** showed the highest potency against hOGA compared with those of compounds **15a–15l**. This suggests that nitrogen atom that is in a suitable position may lead to such molecular conformation that improves the binding affinity. Moreover, compound **15b** had *K*_i_ values of 3.76 µM against hOGA and 30.42 µM against hsHexB, indicating that it could be used as a new leading compound against hOGA in further research.

#### 3.2.2. Kinetic studies

**Figure 4.**
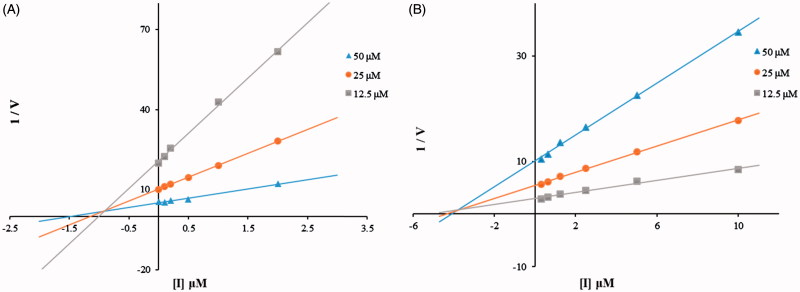
**(**A) Dixon plots for inhibition of hsHexB by compound **15j**. (B) Dixon plots for inhibition of hOGA by compound **15b**.

To explore inhibitory mechanisms toward hsHexB and hOGA by the thioglycoside derivatives, the most potent inhibitors (compound **15j** against hsHexB; and **15b** against hOGA) were selected for kinetic studies by Dixon plots. As shown in Figure 4, the Dixon plots of compound **15j** against hsHexB and compound **15b** against hOGA revealed that these active thioglycosides were competitive inhibitors. Thus, compounds **15j** and **15b** were able to bind to the active pockets of hsHexB and hOGA, respectively.

### 3.3. Molecular docking results

To further explore possible binding modes of compound **15j** to hsHexB and **15b** to hOGA, the molecular docking studies were carried out using Sybyl 7.3 software.

**Figure 5.**
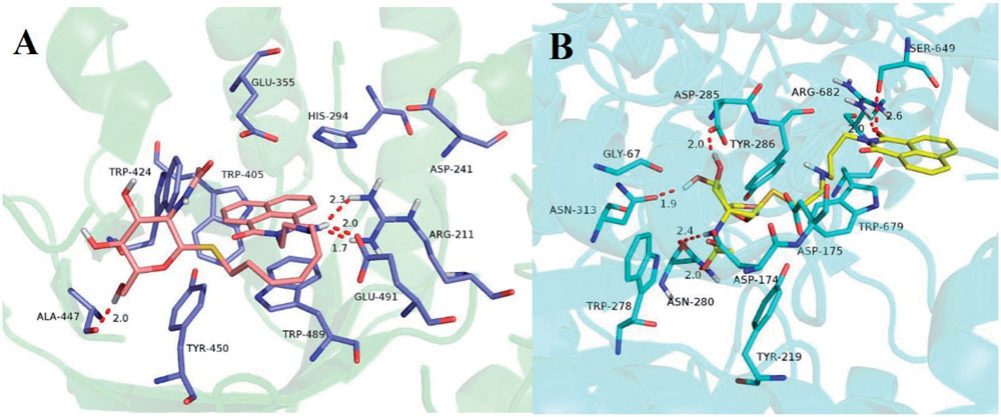
**(**A) Molecular docking of **15j** with hsHexB (PDB ID: 3LMY). The enzyme is presented as cartoon representation, the catalytic residues and compound **15j** are shown as sticks, and hydrogen bonds are highlighted as red dashed lines. Atom colors of **15j**: light pink-carbon atoms, red-oxygen atoms, blue-nitrogen atoms, dark yellow-sulfur atoms. (B) Molecular docking of **15b** with hOGA (PDB ID: 5M7U). The enzyme is presented as cartoon representation, the catalytic residues and compound **15b** are shown as sticks, hydrogen bonds are highlighted as red dashed lines. Atom colors of **15b**: yellow-carbon atoms, red-oxygen atoms, blue-nitrogen atoms, dark yellow-sulfur atoms. The molecular models were created using software PyMOL.

The molecular docking for compound **15j-**hsHexB complex in Figure 5(A) showed that naphthalimide group of compound **15j** bound to the −1 subsite (active pocket) of hsHexB via aromatic π–π stacking interactions with His294, Trp489, and Trp405. Moreover, oxygen of naphthalimide ring formed hydrogen bonds with the catalytic Arg211. These interactions are coherent with those found in the complex structure of hsHexB-Pyrimethamine^36^. In addition, NH in alkylamine linker of **15j** bound to Glu491 via hydrogen bonds at a distance of 2.0 Å. As supported by the enzymatic activity assays, this H-bonding interaction is an important factor contributing to inhibitory potency. When the linker lengths were increased (i.e. from ethylamino in **15j** to propylamino in **15k** or butylamino in **15l)**, the inhibitory potency was considerably decreased. Furthermore, the glycosyl moiety of **15j**, which extended out from the active pocket could help improve the affinity by forming a hydrogen bond with Ala447 at a distance of 2.0 Å. And the additional H-bonding interaction could explain its increasing inhibitory potency when compared with the structure of its parent compound, 2-aminoethyl-naphthalimide (*K*_i_ = 2.09 µM against hsHex)^22^.

Unlike the interactions between compound **15j** and hsHexB, as shown in Figure 5(B), the glycosyl moiety from **15b** was found to be tightly bound to the −1 subsite from hOGA via H-bonding interactions with catalytic Asn313, Asp285, and Asn280. This finding is in incoherent with those found in the complex structure of hOGA–VV347, confirming the accuracy of the docking result^35^. In addition, the naphthalimide group of compound **15b** interacted with residues of domains that were outside the −1 binging-site pocket, notably through hydrogen bondings with Ser649 and Arg682, as well as π–π stacking interaction with Trp679.

## 4. Conclusions

In conclusion, we have presented the design and synthesis of thioglycoside derivatives-containing naphthalimide moiety, and their inhibitory potency against GH20 hsHexB and GH84 hOGA. The efficiency evaluation by enzymatic assay showed that compounds **15j** (*K*_i_ = 0.91 µM against hsHexB; *K*_i_ > 100 µM against hOGA) and **15 b** (*K*_i_ = 3.76 µM against hOGA; *K*_i_ = 30.42 µM against hsHexB) exhibited the highest inhibitory potency against hsHexB and hOGA, respectively. The structure–activity relationship as well as the molecular docking studies provided some insight into the rational design for the two human β-*N*-acetylhexosaminidases. For hsHexB, the 2-aminoethyl- naphthalimide moiety, tightly bound to the −1 binging-site pocket, was found to be the critical factor in maintaining the inhibitory potency. For hOGA, while the glycosyl moiety was bound to the −1 subsite, naphthalimide group with five- to seven-atom linker contain a nitrogen atom that was in such position that led to additional H–bonding and π–π stacking interactions outside of the −1 binding subsite, and these interactions contributed to the selectivity and inhibitory potency. These thioglycoside derivatives may prove to be valuable leading compounds for further developments of new selective inhibitors against hsHexB or hOGA.

## Supplementary Material

IENZ_1419217_Supplementary_Materials.pdf
