# Supplementary material for: Design and synthesis of naphthalimide group-bearing thioglycosides as novel β-N-acetylhexosaminidases inhibitors
Source: J Enzyme Inhib Med Chem. 2018 Feb 2;33(1):445–52. doi: 10.1080/14756366.2017.1419217 (PMC6009855; doi:10.1080/14756366.2017.1419217)
Supplement: IENZ_1419217_Supplementary_Materials.pdf [file IENZ_A_1419217_SM5286.pdf]

Supporting Information

Design and synthesis of naphthalimide group-bearing thioglycosides as novel β-N-acetylhexosaminidases inhibitors

Shengqiang Shen[a], Wei Chen[b], Lili Dong[a], Qing Yang[b]*, Huizhe Lu[a] and Jianjun Zhang[a]*

[a]*Department of Applied Chemistry, College of Science, China Agricultural University, Beijing, China;* [b]*School of Life Science and Biotechnology, Dalian University of Technology, Dalian, China*

CONTACT Jianjun Zhang, E-mail addresses: zhangjianjun@cau.edu.cn, Department of Applied Chemistry, China Agricultural University, Beijing 100193, China; Qing Yang, E-mail addresses: qingyang@dlut.edu.cn, School of Life Science and Biotechnology, Dalian University of Technology, Dalian, China.

	**Page No.**
Synthesis of compound **10**	S-4
Characterization of compound **11a-11d** and **16**	S4-S5
General synthetic procedure for compounds **13a-13c**	S5-S6
Characterization of compound **14a-14l**	S6-S9
Characterization of compound **15b-15l**	S9-S12
General synthetic procedure for compounds **19a-19e**	S12-S13
Characterization of compound **20a-20e**	S13-S14
Characterization of compound **21b-21e**	S15-S16
^{1}H NMR spectrum of compound **10**	S16
^{1}H NMR spectrum of compound **11a**	S16
^{1}H and ^{13}C NMR spectrum of compound **11b**	S17
^{1}H and ^{13}C NMR spectrum of compound **11c**	S18
^{1}H and ^{13}C NMR spectrum of compound **11d**	S19
^{1}H NMR spectrum of compound **11e**	S20

^{1}H NMR spectrum of compound **16**	S21
^{13}C NMR spectrum of compound **16**	S21
^{1}H NMR spectrum of compound **13a**	S22
^{1}H NMR spectrum of compound **13b**	S22
^{1}H and ^{13}C NMR spectrum of compound **13c**	S23
^{1}H and ^{13}C NMR spectrum of compound **14a**	S24
^{1}H and ^{13}C NMR spectrum of compound **14b**	S25
^{1}H and ^{13}C NMR spectrum of compound **14c**	S26
^{1}H and ^{13}C NMR spectrum of compound **14d**	S27
^{1}H and ^{13}C NMR spectrum of compound **14e**	S28
^{1}H and ^{13}C NMR spectrum of compound **14f**	S29
^{1}H and ^{13}C NMR spectrum of compound **14g**	S30
^{1}H and ^{13}C NMR spectrum of compound **14h**	S31
^{1}H and ^{13}C NMR spectrum of compound **14i**	S32
^{1}H and ^{13}C NMR spectrum of compound **14g**	S33
^{1}H and ^{13}C NMR spectrum of compound **14k**	S34
^{1}H and ^{13}C NMR spectrum of compound **14l**	S35
^{1}H and ^{13}C NMR spectrum of compound **15a**	S36
^{1}H and ^{13}C NMR spectrum of compound **15b**	S37
^{1}H and ^{13}C NMR spectrum of compound **15c**	S38
^{1}H and ^{13}C NMR spectrum of compound **15d**	S39
^{1}H and ^{13}C NMR spectrum of compound **15e**	S40
^{1}H and ^{13}C NMR spectrum of compound **15f**	S41
^{1}H and ^{13}C NMR spectrum of compound **15g**	S42
^{1}H and ^{13}C NMR spectrum of compound **15h**	S43
^{1}H and ^{13}C NMR spectrum of compound **15i**	S44
^{1}H and ^{13}C NMR spectrum of compound **15j**	S45

^{1}H and ^{13}C NMR spectrum of compound **15k**	S46
^{1}H and ^{13}C NMR spectrum of compound **15l**	S47
^{1}H and ^{13}C NMR spectrum of compound **17**	S48
^{1}H and ^{13}C NMR spectrum of compound **20a**	S49
^{1}H and ^{13}C NMR spectrum of compound **20b**	S50
^{1}H and ^{13}C NMR spectrum of compound **20c**	S51
^{1}H and ^{13}C NMR spectrum of compound **20d**	S52
^{1}H and ^{13}C NMR spectrum of compound **20e**	S53
^{1}H and ^{13}C NMR spectrum of compound **21a**	S54
^{1}H and ^{13}C NMR spectrum of compound **21b**	S55
^{1}H and ^{13}C NMR spectrum of compound **21c**	S56
^{1}H and ^{13}C NMR spectrum of compound **21d**	S57
^{1}H and ^{13}C NMR spectrum of compound **21e**	S58
Linewaver-Burk plots of **15j** and **15b** (Fig. S1)	S59
References	S59

Synthesis of compound 10

N-Acetyl-D-glucosamine (20 g, 90.5 mmol) was added into acetyl chloride (80 mL, 373.6 mmol) and stirred for 48 h at room temperature, until TLC (EtOAc) indicated that the reaction was completed. The reaction mixture was poured into ice water (500 mL) and DCM (300 mL) was then added. Following phase separation, the organic layer was washed with saturated $NaHCO_3$ aqueous solution (2×100 mL), water (2×100 mL), dried over Na_2SO_4, filtered, and concentrated *in vacuo* to obtain 2-acetamido-3,4,6-tri-O-acetyl-2-deoxy-α-D-glucopyranosyl chloride, which was used without further purification.

2-acetamido-3,4,6-tri-O-acetyl-2-deoxy-α-D-glucopyranosyl chloride (10 g, 28.3 mmol) and thiourea (3.9 g, 51.2 mmol) were mixed in acetone (120 mL). The mixture was heated to reflux for 4 h until a white precipitate was observed. The solid precipitate was then filtered and washed with acetone (2×20 mL). As a result, 2-acetamido-3,4,6-tri-O-acetyl-β-D-glucopyranosyl-1-isothiouronium chloride was obtained. (10.5g, 86.9%) yield; $[\alpha]_D^{25}$ -25.1 (c=1.0, H_2O) [Lit. $[\alpha]_D^{25}$ -22.8 (c=1.0, H_2O)][1]; mp175-177°C; ^{1}H NMR (300 MHz, DMSO-d_6) δ ppm 9.45(s, 2H, NH_2), 9.19(s, 2H, NH_2), 8.46 (d, J = 9.3 Hz, 1H, NH), 5.65 (d, J = 9.7 Hz, 1H, H-1), 5.14 (t, J = 9.8 Hz, 1H, H-3), 4.94 (t, J = 9.7 Hz, 1H, H-4), 4.17–4.27 (m, 2H, H-5/H-6a), 3.92–4.13 (m, 2H, H-2/ H-6b), 1.91, 1.97, 2.03 (3s, 9H, 3 OAc), 1.79 (s, 3H, NAc).

A mixture of 2-acetamido-3,4,6-tri-O-acetyl-β-D-glucopyranosyl-1-isothiouronium chloride (5 g, 11.3 mmol) and sodium metabisulfite (4.3 g, 22.6 mmol) in DCM (60 mL) and H_2O (40 mL) was

heated to reflux for 3 h, until TLC (EtOAc) indicated that the reaction was completed. Prior to phase separation, the reaction mixture was cooled down to room temperature. After the aqueous layer underwent another round of extraction with DCM (60mL), the resulting organic layers (from both rounds) were combined and washed with H_2O (50 mL), and further dried over N15bSO_4. The resulting white solid was harvested by filtration, concentration, and recrystallized from acetate/petrol; and the final 2-acetamido-3,4,6-tri-O-acetyl-2-deoxy-1-thio-β- D-glucopyranose (**10**) was obtained. (3.7 g, 90.2 %) yield; $[\alpha]_D^{25}$ -15.1 (c=1.0,$CHCl_3$) [Lit. $[\alpha]_D^{23}$ -16.0 (c=1.08,$CHCl_3$)][2]; mp165-167°C; ^{1}H NMR (300 MHz, $CDCl_3$) δ 5.63 (d, *J* = 9.1 Hz, 1H, NH), 5.26–5.10 (m, 2H, H-3, H-4), 4.89 (d, *J* = 9.1 Hz, 1H, SH), 4.68 (t, *J* = 9.0 Hz, 1H, H-1), 4.33–4.15 (m, 3H, H-6a, H-2, H-6b), 3.76 (m, 1H, H-5), 2.11, 2.06，2.05 (3 s, 9H, 3 OAc), 1.98 (s, 3H, NHAc).

Characterization of Compound 11a-11d and 16

*2-Bromoethyl 2-acetamido-3,4,6-tri-O-acetyl-2-deoxy-1-thio-β-D-glucopyranoside (**11a**)*

White solid; (1.9g, 73.6 %) yield; $[\alpha]_D^{25}$ -43.1 (c=0.5,$CHCl_3$) [Lit. $[\alpha]_D^{20}$ -42.5 (c=0.48,$CHCl_3$)][3]; mp186-188°C; ^{1}H NMR (300 MHz, $CDCl_3$) δ 5.55 (d, *J* = 9.6 Hz, 1H, NH), 5.17-5.07 (m, 2H, H-3, H-4), 4.67 (d, *J* = 10.3 Hz, 1H, H-1), 4.17 (d, *J* = 4.0 Hz, 2H, H-6a, H-6b), 4.08 (td, *J* = 10.1, 9.1 Hz, 1H, H-2), 3.72 (dt, *J* = 9.8, 4.0 Hz, 1H, H-5), 3.66-3.48 (m, 2H, CH_2Br), 3.25-2.94 (m, 2H, SCH_2), 2.12, 2.04, 2.03 (3 s, 9H, 3 OAc), 1.96 (s, 3H, NHAc).

*3-bromopropyl2-acetamido-3,4,6-tri-O-acetyl-2-deoxy-1-thio-β-Dglucopyranoside (**11b**)*

White solid; (2.0g, 75.2 %) yield; $[\alpha]_D^{25}$ -55.1 (c=0.41,$CHCl_3$); mp162-164°C; ^{1}H NMR (300 MHz, $CDCl_3$) δ 6.17 (d, *J* = 9.3 Hz, 1H, NH), 5.23 (t, *J* = 9.8 Hz, 1H, H-3), 5.08 (t, *J* = 9.7 Hz, 1H, H-4), 4.70 (d, *J* = 10.4 Hz, 1H, H-1), 4.27–4.02 (m, 3H, H-6a, H-6b, H-2), 3.75 (ddd, *J* = 9.9, 4.8, 2.4 Hz, 1H, H-5), 3.54 (t, *J* = 6.4 Hz, 2H, CH_2Br), 2.98 –2.74(m, 2H, SCH_2), 2.16 (m, 2H, CH_2), 2.09, 2.04, 2.03 (3 s, 9H, 3 OAc), 1.97 (s, 3H, NHAc); ^{13}C NMR (75 MHz, $CDCl_3$) δ 170.58, 170.30, 169.93, 168.96, 84.42, 75.50, 73.34, 68.21, 62.02, 52.98, 32.38, 31.82, 28.42, 22.88, 20.41, 20.33, 20.24; HRMS (ESI) calcd for $C_{17}H_{27}BrNO_8S$ (M+H^+) 484.0641, found 484.0647.

*5-bromopentyl2-acetamido-3,4,6-tri-O-acetyl-2-deoxy-1-thio-β-D-glucopyranoside (**11c**)*

White solid; (2.1g, 74.5 %) yield; $[\alpha]_D^{25}$ -63.2 (c=0.5,$CHCl_3$); mp148-150°C; ^{1}H NMR (300 MHz, $CDCl_3$) δ 6.26 (d, *J* = 9.3 Hz, 1H, NH), 5.23 (t, *J* = 9.8 Hz, 1H, H-3), 5.08 (t, *J* = 9.7 Hz, 1H, H-4), 4.67 (d, *J* = 10.4 Hz, 1H, H-1), 4.25 (dd, J = 12.3, 5.0 Hz, 1H, H-6b), 4.18–4.02 (m, 2H, H-6a, H-2), 3.75 (ddd, *J* = 9.9, 4.9, 2.3 Hz, 1H, H-5), 3.42 (t, *J* = 6.7 Hz, 2H, CH_2Br), 2.83–2.61 (m, 2H, SCH_2), 2.09, 2.04, 2.03 (3 s, 9H, 3 OAc), 1.97 (s, 3H, NHAc), 1.87 (m, 2H, CH_2), 1.71–1.60 (m, 2H, CH_2), 1.60–1.46 (m, 2H, CH_2); ^{13}C NMR (75 MHz, $CDCl_3$) δ 170.50, 170.27, 169.90, 168.95, 83.94, 75.43, 73.44, 68.28, 62.05, 52.86, 33.19, 31.81, 29.25, 28.34, 26.88, 22.86, 20.39, 20.32, 20.23; HRMS (ESI) calcd for $C_{19}H_{31}BrNO_8S$ (M+H^+) 512.0954, found 512.0949.

*6-bromohexyl 2-acetamido-3,4,6-tri-O-acetyl-2-deoxy-1-thio-β-D-glucopyranoside (**11d**)*

White solid; (2.0g, 69.0 %) yield; $[\alpha]_D^{25}$ -91.1 (c=0.58,$CHCl_3$); mp152-154°C; ^{1}H NMR (300 MHz, DMSO-d_6) δ 7.96 (d, *J* = 9.4 Hz, 1H, NH), 5.07 (t, *J* = 9.8 Hz, 1H, H-3), 4.83 (t, *J* = 9.7 Hz, 1H, H-4), 4.67 (d, *J* = 10.4 Hz, 1H, H-1), 4.14 (dd, *J* = 12.3, 5.1 Hz, 1H, H-6b), 4.01 (dd, *J* = 12.2, 2.2 Hz, 1H, H-6a), 3.91–3.76 (m, 2H, H-2, H-5), 3.51 (t, *J* = 6.7 Hz, 2H, CH_2Br), 2.69–2.52 (m, 2H, SCH_2), 2.00, 1.96, 1.91 (3 s, 9H, 3 OAc), 1.76 (s, 3H, NHAc), 1.85–1.72 (m, 2H, CH_2), 1.61–1.47 (m, 2H, CH_2), 1.43–1.27 (m, 4H, 2 CH_2); ^{13}C NMR (75 MHz, DMSO-d_6) δ 170.06, 169.71, 169.36, 169.15, 83.56, 74.72, 73.81, 68.73, 62.18, 52.30, 35.11, 32.24, 29.27, 29.20, 27.32, 27.16, 22.73, 20.61, 20.50, 20.43; HRMS (ESI) calcd for $C_{20}H_{33}BrNO_8S$ ($M+H^+$) 526.1110, found 526.1118.

*1,4-bis[(2-acetamido-3,4,6-tri-O-acetyl-2-deoxy-β-D-glucopyranosyl)thio]butane (**16**)*

White solid; (1.3g, 59.1%) yield; $[\alpha]_D^{25}$ -72.5 (c=0.98,$CHCl_3$); mp177-179°C; ^{1}H NMR (300 MHz, $CDCl_3$) δ 7.97 (d, *J* = 9.4 Hz, 2H, 2 NHAc), 5.06 (t, *J* = 9.8 Hz, 2H, 2 H-3), 4.83 (t, *J* = 9.7 Hz, 2H, 2 H), 4.67 (d, *J* = 10.4 Hz, 2H, 2 H-1), 4.15 (dd, *J* = 12.3, 5.0 Hz, 2H, 2 H-6b), 4.01 (dd, *J* = 12.2, 1.8 Hz, 2H, 2 H-6a), 3.92–3.71 (m, 4H, 2 H-2, 2 H-5), 2.72–2.52 (m, 4H, 2 CH_2), 2.01, 1.97, 1.91 (3 s, 18H, 6 OAc), 1.76(s, 6H, NAc), 1.67–1.52 (m, 4H, 2 CH_2); ^{13}C NMR (75 MHz, $CDCl_3$) δ 170.15, 169.76, 169.41, 169.25, 83.51, 74.72, 73.78, 68.70, 62.15, 52.31, 28.89, 28.39, 22.74, 20.63, 20.52, 20.44. HRMS (ESI) calcd for $C_{32}H_{49}N_2O_{16}$ S_2 ($M+H^+$) 781.2518, found 781.2518.

General synthetic procedure for compounds 13a-13c

α, ω- diamino-alkane (75.6 mmol, 3.0 eq) was added into a solution of 1,8-naphthalic anhydride **12** (25.2 mmol, 1 eq) in ethanol (150 mL), and the mixture was refluxed for 4 h, until the TLC (EtOAc/ MeOH=6/1) indicated that the reaction was completed. After that, the hot reaction mixture was filtered, and the filtrate was cooled down to room temperature so that a precipitate was observed. The precipitate was then filtered, dried, and recrystallized from ethanol, and as a result, the final compounds **13a-13c** were obtained.

*2-(2-aminoethyl)-1H-benzo[de]isoquinoline-1,3(2H)-dione (**13a**)*

Light yellow solid; (4.7g, 77.6 %) yield; mp140-142°C [Lit. mp 129-130°C][4]; ^{1}H NMR (300 MHz, DMSO-d_6) δ 8.50–8.43 (m, 4H, ArH), 7.87 (dd, *J* = 8.2, 7.3 Hz, 2H, ArH), 4.11–4.02 (m, 2H, CH_2), 2.86–2.75 (m, 2H, CH_2).

*2-(3-aminopropyl)-1H-benzo[de]isoquinoline-1,3(2H)-dione (**13b**)*

White solid; (4.1g, 64.1%) yield; mp141-143°C [Lit. mp 141-142°C][4]; ^{1}H NMR (300 MHz, $CDCl_3$) δ 8.63 (d, *J* = 7.3 Hz, 2H, ArH), 8.24 (d, *J* = 7.9 Hz, 2H, ArH), 7.77 (m, 2H, ArH), 4.31 (t, *J* = 6.9 Hz, 2H, CH_2), 2.79 (t, *J* = 6.6 Hz, 2H, CH_2), 1.99–1.87 (m, 2H, CH_2).

*2-(4-aminobutyl)-1H-benzo[de]isoquinoline-1,3(2H)-dione (**13c**)*

White solid; (4.5g, 66.7%) yield; mp131-133°C [Lit. mp 129-130°C][4]; ^{1}H NMR (300 MHz, DMSO-d_6) δ 8.46–8.32 (m, 4H, ArH), 7.86–7.74 (m, 2H, ArH), 4.05–3.94 (m, 2H, CH_2), 2.73–2.50 (m, 4H, CH_2), 1.71–1.55 (m, 2H, CH_2), 1.47–1.31 (m, 2H, CH_2). ^{13}C NMR (75 MHz, DMSO-d_6) δ 163.40, 134.30, 131.31, 130.74, 127.34, 127.23, 122.04, 41.57, 38.82, 30.95, 25.23.

Characterization of compound 14a-14l

*2-[2-[2-[(2-acetamido-3,4,6-tri-O-acetyl-β-D-glucopyranosyl)thio]ethylamino] ethyl]-1H-benzo [de]isoquinoline-1,3(2H)-dione (**14a**)*

White solid; (0.88g, 69.8%) yield; $[\alpha]_D^{25}$ -37.1 (c=0.5,$CHCl_3$); mp158-160°C; ^{1}H NMR (300 MHz, DMSO-d_6) δ 8.57–8.44 (m, 4H, ArH), 7.99 (d, J = 9.3 Hz, 1H, NH), 7.93–7.83 (m, 2H, ArH), 5.07 (t, J = 9.7 Hz, 1H, H-3), 4.86 (t, J = 9.8 Hz, 1H, H-4), 4.71 (d, J = 10.5 Hz, 1H, H-1), 4.22–4.09 (m, 3H, H-6b, CH_2NC=O), 4.00 (dd, J = 12.2, 2.2 Hz, 1H, H-6a), 3.92–3.81 (m, 2H, H-2, H-5), 2.92–2.78 (m, 4H, 2 CH_2), 2.77–2.57 (m, 2H, SCH_2), 1.98, 1.97, 1.92 (3 s, 9H, 3 OAc), 1.76 (s, 3H, NAc); ^{13}C NMR (75 MHz, DMSO-d_6) δ 170.13, 169.75, 169.39, 169.24, 163.65, 134.35, 131.35, 130.77, 127.47, 127.26, 122.19, 83.60, 74.76, 73.86, 68.67, 62.15, 52.23, 48.78, 46.14, 39.11, 29.17, 22.74, 20.56, 20.53, 20.45; HRMS (ESI) calcd for $C_{30}H_{36}N_3O_{10}$ S (M+H^+) 630.2116, found 630.2120.

*2-[3-[2-[(2-acetamido-3,4,6-tri-O-acetyl-β-D-glucopyranosyl)thio]ethylamino] propyl]-1H-benzo [de]isoquinoline-1,3(2H)-dione (**14b**)*

Light yellow solid; (0.91g, 70.7%) yield; $[\alpha]_D^{25}$ -32.3 (c=0.41,$CHCl_3$); mp144-146°C; ^{1}H NMR (300 MHz, DMSO-d_6) δ 8.42–8.30 (m, 4H, ArH), 8.02 (d, J = 9.4 Hz, 1H, NH), 7.82–7.73 (m, 2H, ArH), 5.09 (t, J = 9.7 Hz, 1H, H-3), 4.85 (t, J = 9.7 Hz, 1H, H-4), 4.74 (d, J = 10.4 Hz, 1H, H-1), 4.14 (dd, J = 12.3, 5.0 Hz, 1H, H-6b), 4.08–3.96 (m, 3H, H-6a, CH_2NC=O), 3.93–3.81 (m, 2H, H-2, H-5), 2.83–2.73 (m, 2H, CH_2), 2.73–2.56 (m, 4H, SCH_2, CH_2), 1.98, 1.97, 1.92 (3 s, 9H, 3 OAc), 1.77 (s, 3H, NAc), 1.84–1.69 (m, 2H, CH_2); ^{13}C NMR (75 MHz, DMSO-d_6) δ 169.95, 169.60, 169.23, 169.09, 163.29, 134.11, 131.11, 130.53, 127.17, 127.02, 121.87, 83.47, 74.59, 73.70, 68.54, 61.99, 52.17, 48.80, 46.23, 37.98, 29.40, 27.69, 22.59, 20.40, 20.35, 20.29; HRMS (ESI) calcd for $C_{31}H_{38}N_3O_{10}$ S (M+H^+) 644.2272, found 644.2268.

*2-[4-[2-[(2-acetamido-3,4,6-tri-O-acetyl-β-D-glucopyranosyl)thio]ethylamino] butyl]-1H-benzo [de]isoquinoline-1,3(2H)-dione (**14c**)*

Light yellow solid; (0.92g, 70.0%) yield; $[\alpha]_D^{25}$ -42.3 (c=0.31,$CHCl_3$); mp161-163°C; ^{1}H NMR (300 MHz, $CDCl_3$) δ 8.41 (d, J = 7.2 Hz, 2H, ArH), 8.07 (d, J = 7.9 Hz, 2H, ArH), 7.62 (t, J = 7.8 Hz, 2H, ArH), 7.51 (d, J= 9.6 Hz, 1H, NH), 5.18 (t, J = 9.7 Hz, 1H, H-3), 5.04 (t, J = 9.7 Hz, 1H, H-4), 4.95 (d, J = 10.4 Hz, 1H, H-1), 4.28–4.00 (m, 5H, H-6b, H-6a, H-2,CH_2NC=O), 3.84 (m, 1H, H-5), 3.25-2.91 (m, 6H, 2 CH_2, SCH_2), 2.00, 1.94, 1.88 (3 s, 9H, 3 OAc), 1.86 (s, 3H,

NAc), 1.86-1.72 (m, 4H, 2 CH_2); ^{13}C NMR (75 MHz, $CDCl_3$) δ 170.51, 170.40, 170.29, 169.05, 163.72, 133.65, 131.07, 130.83, 127.56, 126.55, 121.92, 84.12, 77.02, 75.44, 73.75, 68.08, 61.62, 52.50, 47.26, 39.05, 27.57, 24.96, 23.86, 22.79, 20.43, 20.32, 20.25; HRMS (ESI) calcd for $C_{32}H_{40}N_3O_{10}$ S ($M+H^+$) 658.2434, found 658.2441.

*2-[2-[3-[(2-acetamido-3,4,6-tri-O-acetyl-β-D-glucopyranosyl)thio]propylamino] ethyl]-1H-benzo [de]isoquinoline-1,3(2H)-dione (**14d**)*

White solid; (0.89g, 69.2%) yield; $[\alpha]_D^{25}$ -33.1 (c=0.5,$CHCl_3$); mp157-159°C; ^{1}H NMR (300 MHz, DMSO-*d₆*) δ 8.47 (m, 4H, ArH), 8.05 (d, *J* = 9.4 Hz, 1H, NH), 7.92–7.82 (m, 2H, ArH), 5.07 (t, *J* = 9.7 Hz, 1H, H-3), 4.84 (t, *J* = 9.7 Hz, 1H, H-4), 4.72 (d, *J* = 10.4 Hz, 1H, H-1), 4.26 (t, *J* = 6.2 Hz, 2H, CH_2NC=O), 4.15 (dd, *J* = 12.3, 4.9 Hz, 1H, H-6b), 4.01 (dd, *J* = 12.1, 1.9 Hz, 1H, H-6a), 3.92–3.79 (m, 2H, H-2, H-5), 3.05 (t, *J* = 6.1 Hz, 2H, CH_2), 2.83 (t, *J* = 6.9 Hz, 2H, CH_2), 2.76–2.55 (m, 2H, SCH_2), 2.00, 1.97, 1.91 (3 s, 9H, 3 OAc), 1.88–1.78 (m, 2H, CH_2), 1.76 (s, 3H, NAc); ^{13}C NMR (75 MHz, DMSO-d_6) δ 170.00, 169.57, 169.24, 169.11, 163.75, 134.31, 131.25, 130.65, 127.44, 127.15, 122.16, 83.25, 74.57, 73.66, 68.53, 61.96, 52.09, 46.62, 45.72, 37.64, 27.45, 26.73, 22.58, 20.46, 20.36, 20.29; HRMS (ESI) calcd for $C_{31}H_{38}N_3O_{10}$ S ($M+H^+$) 644.2272, found 644.2277.

*2-[3-[3-[(2-acetamido-3,4,6-tri-O-acetyl-β-D-glucopyranosyl)thio]propylamino]propyl]-1H-benzo [de]isoquinoline-1,3(2H)-dione (**14e**)*

White solid; (0.88g, 67.1%) yield; $[\alpha]_D^{25}$ -39.5 (c=0.48,$CHCl_3$); mp132-134°C; ^{1}H NMR (300 MHz, DMSO-d_6) δ 8.46–8.37 (m, 4H, ArH), 8.08 (d, *J* = 9.4 Hz, 1H, NH), 7.88–7.77 (m, 2H, ArH), 5.07 (t, *J* = 9.7 Hz, 1H, H-3), 4.84 (t, *J* = 9.7 Hz, 1H, H-4), 4.74 (d, *J* = 10.4 Hz, 1H, H-1), 4.21–3.94 (m, 4H, H-6b, H-6a, CH_2NC=O), 3.92–3.78 (m, 2H, H-2, H-5), 2.94–2.79 (m, 4H, 2 CH_2), 2.78–2.56 (m, 2H, SCH_2), 1.99, 1.97, 1.91 (3 s, 9H, 3 OAc), 1.99–1.96 (m, 2H, CH_2), 1.89–1.81 (m, 2H, CH_2), 1.75 (s, 3H, NAc); ^{13}C NMR (75 MHz, DMSO-d_6) δ 170.14, 169.71, 169.39, 169.29, 163.66, 134.41, 131.34, 130.78, 127.46, 127.25, 122.10, 83.35, 74.75, 73.83, 68.67, 62.10, 59.85, 52.22, 46.22, 45.45, 37.48, 26.77, 25.51, 22.71, 20.61, 20.51, 20.44; HRMS (ESI) calcd for $C_{32}H_{40}N_3O_{10}$ S ($M+H^+$) 658.2434, found 658.2429.

*2-[4-[3-[(2-acetamido-3,4,6-tri-O-acetyl-β-D-glucopyranosyl)thio]propylamino]butyl]-1H-benzo [de]isoquinoline-1,3(2H)-dione (**14f**)*

White solid; (0.92g, 68.5%) yield; $[\alpha]_D^{25}$ -49.7 (c=0.55,$CHCl_3$); mp142-144°C; ^{1}H NMR (300 MHz, $CDCl_3$) δ 8.43–8.35 (m, 2H, ArH), 8.05 (d, *J* = 8.2 Hz, 2H, ArH), 7.77 (d, *J* = 9.5 Hz, 1H, NH), 7.66–7.56 (m, 2H, ArH), 5.22 (t, *J* = 9.7 Hz, 1H, H-3), 5.04 (t, *J* = 9.7 Hz, 1H, H-4), 4.95 (d, *J* = 10.4 Hz, 1H, H-1), 4.28–4.00 (m, 5H, H-6b, H-6a, CH_2NC=O, H-2), 3.88–3.77 (m, 1H, H-5), 3.39–3.06 (m, 4H, 2 CH_2), 3.04–2.93 (m, 1H, SCH_2), 2.78–2.58 (m, 1H, SCH_2), 2.62-2.19 (m, 2H, CH_2), 2.01, 1.98, 1.95 (3 s, 9H, 3 OAc), 1.94(s, 3H, NAc), 1.99–1.97 (m, 2H, CH_2), 1.88–1.73 (m, 2H, CH_2). ^{13}C NMR (75 MHz, $CDCl_3$) δ 170.90, 170.43, 170.19, 169.07, 163.70, 133.67, 131.04, 130.84, 127.53, 126.55, 121.84, 82.95, 75.25, 73.79, 68.31, 61.88, 52.49, 47.33, 46.05, 38.94,

26.66, 24.85, 24.68, 23.14, 22.89, 20.43, 20.32, 20.23; HRMS (ESI) calcd for $C_{33}H_{42}N_3O_{10}$ S (M+H$^+$) 672.2591, found 672.2593.

*2-[2-[5-[(2-acetamido-3,4,6-tri-O-acetyl-β-D-glucopyranosyl)thio]pentylamino] ethyl]-1H-benzo [de]isoquinoline-1,3(2H)-dione (**14g**)*

White solid; (0.96g, 71.7%) yield; $[\alpha]_D^{25}$ -34.8 (c=0.56,$CHCl_3$); mp151-153°C; ^{1}H NMR (300 MHz, DMSO-d_6) δ 8.46–8.33 (m, 4H, ArH), 7.98 (d, *J* = 9.4 Hz, 1H, NH), 7.87–7.75 (m, 2H, ArH), 5.06 (t, *J* = 9.8 Hz, 1H, H-3), 4.83 (t, *J* = 9.7 Hz, 1H, H-4), 4.67 (d, *J* = 10.4 Hz, 1H, H-1), 4.21–3.98 (m, 4H, H-6b, H-6a, CH_2NC=O), 3.93–3.76 (m, 2H, H-2, H-5), 3.37–3.03 (m, 2H, CH_2), 2.77 (t, *J* = 6.8 Hz, 2H, CH_2), 2.66–2.53 (m, 2H, SCH_2), 1.99, 1.97, 1.96 (3 s, 9H, 3 OAc), 1.77(s, 3H, NAc), 1.61–1.42 (m, 2H, CH_2), 1.42–1.22 (m, 4H, 2 CH_2); ^{13}C NMR (75 MHz, DMSO-d_6) δ 170.10, 169.75, 169.39, 169.20, 163.55, 134.24, 131.31, 130.70, 127.41, 127.20, 122.17, 83.57, 74.72, 73.84, 68.75, 62.18, 52.35, 48.90, 46.88, 39.47, 29.36, 29.16, 27.32, 26.11, 22.74, 20.57, 20.51, 20.44; HRMS (ESI) calcd for $C_{33}H_{42}N_3O_{10}$ S (M+H$^+$) 672.2591, found 672.2599.

*2-[3-[5-[(2-acetamido-3,4,6-tri-O-acetyl-β-D-glucopyranosyl)thio]pentylamino] propyl]-1H-benzo [de]isoquinoline-1,3(2H)-dione (**14h**)*

White solid; (0.97g, 70.6%) yield; $[\alpha]_D^{25}$ -37.2 (c=0.44,$CHCl_3$); mp127-129°C; ^{1}H NMR (300 MHz, DMSO-d_6) δ 8.40–8.34 (m, 4H, ArH), 8.21 (d, *J* = 9.4 Hz, 1H, NH), 7.81–7.74 (m, 2H, ArH), 5.06 (t, *J* = 9.7 Hz, 1H, H-3), 4.86–4.66 (m, 2H, H-4, H-1), 4.15–3.93 (m, 4H, H-6b, H-6a, CH_2NC=O), 3.87–3.73 (m, 2H, H-2, H-5), 2.94–2.70 (m, 4H, 2 CH_2), 2.63–2.50 (m, 2H, SCH_2), 2.12–1.98 (m, 2H, CH_2), 1.96, 1.93, 1.87 (3 s, 9H, 3 OAc), 1.74(s, 3H, NAc), 1.67–1.54 (m, 2H, CH_2), 1.54–1.39 (m, 2H, CH_2), 1.38–1.24 (m, 2H, CH_2); ^{13}C NMR (75 MHz, DMSO-d_6) δ 170.16, 169.68, 169.44, 169.38, 163.60, 134.45, 131.27, 130.77, 127.35, 127.23, 121.91, 83.57, 74.61, 73.89, 68.72, 62.09, 52.37, 46.72, 45.04, 29.30, 28.88, 26.15, 25.29, 25.21, 24.76, 22.75, 20.65, 20.52, 20.47; HRMS (ESI) calcd for $C_{34}H_{44}N_3O_{10}$ S (M+H$^+$) 686.2747, found 686.2739.

*2-[4-[5-[(2-acetamido-3,4,6-tri-O-acetyl-β-D-glucopyranosyl)thio]pentylamino] butyl]-1H-benzo [de]isoquinoline-1,3(2H)-dione (**14i**)*

Light yellow solid; (0.97g, 70.6%) yield; $[\alpha]_D^{25}$ -45.7 (c=0.47,$CHCl_3$); mp142-144°C; ^{1}H NMR (300 MHz, DMSO-d_6) δ 8.47–8.36 (m, 4H, ArH), 8.09 (d, *J* = 9.4 Hz, 1H, NH), 7.88–7.77 (m, 2H, ArH), 5.08 (t, *J* = 9.7 Hz, 1H, H-3), 4.83 (t, *J* = 9.7 Hz, 1H, H-4), 4.72 (d, *J* = 10.4 Hz, 1H, H-1), 4.14 (dd, *J* = 12.3, 5.0 Hz, 1H, H-6b), 4.12–3.95 (m, 3H, H-6a, CH_2NC=O), 3.90–3.77 (m, 2H, H-2, H-5), 2.97–2.74 (m, 4H, 2 CH_2), 2.68–2.52 (m, 2H, SCH_2), 2.00, 1.96, 1.90 (3 s, 9H, 3 OAc), 1.76(s, 3H, NAc), 1.73–1.65 (m, 2H, CH_2), 1.65–1.43 (m, 4H, 2 CH_2), 1.42–1.30 (m, 2H, CH_2), 1.30–1.10 (m, 2H, CH_2); ^{13}C NMR (75 MHz, DMSO-d_6) δ 170.12, 169.69, 169.39, 169.24, 163.51, 134.43, 131.36, 130.82, 127.41, 127.28, 122.03, 83.57, 74.70, 73.87, 68.74, 62.15, 52.33, 46.73, 46.63, 29.18, 28.88, 27.32, 25.30, 25.13, 25.02, 23.38, 22.77, 20.66, 20.52, 20.45; HRMS (ESI) calcd for $C_{35}H_{46}N_3O_{10}$ S (M+H$^+$) 700.2904, found 700.2911.

*2-[2-[6-[(2-acetamido-3,4,6-tri-O-acetyl-β-D-glucopyranosyl)thio]hexylamino] ethyl]-1H-benzo [de]isoquinoline-1,3(2H)-dione (**14j**)*

Light yellow solid; (0.92g, 67.1%) yield; $[\alpha]_D^{25}$ -44.7 (c=0.5,$CHCl_3$); mp158-160°C; ^1H NMR (300 MHz, DMSO-*d₆*) δ 8.56–8.41 (m, 4H, ArH), 7.98 (d, *J* = 9.5 Hz, 1H, NH), 7.92–7.81 (m, 2H, ArH), 5.05 (d, *J* = 9.8 Hz, 1H, H-3), 4.84 (d, *J* = 9.9 Hz, 1H, H-4), 4.66 (d, *J* = 10.4 Hz, 1H, H-1), 4.24–4.09 (m, 3H, H-6b, H-6a, CH_2NC=O), 4.05–3.98 (m, 1H, CH_2NC=O), 3.91–3.77 (m, 2H, H-2, H-5), 2.88 (t, *J* = 6.5 Hz, 2H, CH_2), 2.67–2.51 (m, 4H, CH_2, SCH_2), 2.00, 1.96, 1.91 (3 s, 9H, 3 OAc), 1.76(s, 3H, NAc), 1.53–1.32 (m, 4H, 2 CH_2), 1.31–1.19 (m, 4H, 2 CH_2); ^{13}C NMR (75 MHz, DMSO-d_6) δ 170.10, 169.74, 169.41, 169.21, 163.81, 134.23, 131.28, 130.62, 127.16, 122.26, 122.15, 83.56, 74.71, 73.85, 68.76, 62.20, 52.33, 48.61, 46.54, 38.71, 29.32, 29.01, 28.19, 26.33, 22.75, 20.59, 20.52, 20.45; HRMS (ESI) calcd for $C_{34}H_{44}N_3O_{10}$ S ($M+H^+$) 686.2747, found 686.2755.

*2-[3-[6-[(2-acetamido-3,4,6-tri-O-acetyl-β-D-glucopyranosyl)thio]hexylamino] propyl]-1H-benzo [de]isoquinoline-1,3(2H)-dione (**14k**)*

Light yellow solid; (0.93g, 69.5%) yield; $[\alpha]_D^{25}$ -38.4 (c=0.42,$CHCl_3$); mp164-166°C; ^1H NMR (300 MHz, DMSO-d_6) δ 8.43 (m, 4H, ArH), 8.05 (d, *J* = 9.4 Hz, 1H, NH), 7.90–7.79 (m, 2H, ArH), 5.07 (t, *J* = 9.8 Hz, 2H, H-3), 4.82 (t, *J* = 9.7 Hz, 1H, H-4), 4.69 (d, *J* = 10.4 Hz, 1H, H-1), 4.19–3.96 (m, 4H, H-6b, H-6a, CH_2NC=O), 3.90–3.78 (m, 2H, H-2, H-5), 2.87–2.74 (m, 2H, CH_2), 2.72–2.61 (m, 2H, CH_2), 2.61–2.51 (m, 2H, SCH_2), 1.99, 1.97, 1.91 (3 s, 9H, 3 OAc), 2.04–1.86 (m, 2H, CH_2), 1.76(s, 3H, NAc), 1.59 –1.40 (m, 4H, 2 CH_2), 1.36–1.20 (m, 4H, 2 CH_2); ^{13}C NMR (75 MHz, DMSO-d_6) δ 170.10, 169.72, 169.40, 169.21, 163.66, 134.41, 131.38, 130.79, 127.48, 127.28, 122.15, 83.52, 74.70, 73.84, 68.74, 62.17, 52.31, 47.64, 45.70, 37.68, 29.24, 29.16, 27.91, 27.06, 25.94, 22.75, 22.08, 20.61, 20.52, 20.45; HRMS (ESI) calcd for $C_{35}H_{46}N_3O_{10}$ S ($M+H^+$) 700.2904, found 700.2900.

*2-[4-[6-[(2-acetamido-3,4,6-tri-O-acetyl-β-D-glucopyranosyl)thio]hexylamino] butyl]-1H-benzo [de]isoquinoline-1,3(2H)-dione (**14l**)*

Light yellow solid; (0.94g, 65.6%) yield; $[\alpha]_D^{25}$ -31.1 (c=0.37,$CHCl_3$); mp186-188°C; ^1H NMR (300 MHz, DMSO-d_6) δ 8.49–8.35 (m, 4H, ArH), 8.08 (d, *J* = 9.5 Hz, 1H, NH), 7.86–7.77 (m, 2H, ArH), 5.08 (t, *J* = 9.8 Hz, 1H, H-3), 4.83 (t, *J* = 9.7 Hz, 1H, H-4), 4.70 (d, *J* = 10.4 Hz, 1H, H-1), 4.14 (dd, *J* = 12.3, 5.0 Hz, 1H, H-6b), 4.06–3.96 (m, 3H, CH_2NC=O, H-6a), 3.91–3.79 (m, 2H, H-2, H-5), 2.89–2.67 (m, 4H, 2 CH_2), 2.65–2.51 (m, 2H, CH_2), 1.99, 1.97, 1.91 (3 s, 9H, 3 OAc), 1.76(s, 3H, NHAc), 1.72–1.60 (m, 4H, 2 CH_2), 1.58–1.44 (m, 4H, 2 CH_2), 1.36–1.20 (m, 4H, 2 CH_2). ^{13}C NMR (75 MHz, DMSO-d_6) δ 170.12, 169.69, 169.39, 169.24, 163.51, 134.43, 131.36, 130.82, 127.41, 127.28, 122.03, 83.57, 74.70, 73.87, 68.74, 62.15, 52.33, 46.73, 46.63, 37.68, 29.18, 28.88, 27.32, 25.30, 25.13, 25.02, 23.38, 22.77, 20.66, 20.52, 20.45; HRMS (ESI) calcd for $C_{36}H_{48}N_3O_{10}$ S ($M+H^+$) 714.3060, found 714.3067.

Characterization of compound 15b-15l

*2-[3-[2-[(2-acetamido-β-D-glucopyranosyl)thio]ethylamino]propyl]-1H-benzo[de]isoquinoline-1,3(2H)-dione (**15b**)*

White solid; (0.48g, 92.3%) yield; $[\alpha]_D^{25}$ -15.2 (c=0.10, DMF); mp 218-220°C; ^{1}H NMR (300 MHz, DMSO-d_6) δ 8.52–8.37 (m, 4H, ArH), 7.88–7.79 (m, 2H, ArH), 7.71 (d, *J* = 9.3 Hz, 1H, NHAc), 5.08–4.93 (m, 2H, 2 OH), 4.52 (br s, 1H, OH), 4.36 (d, *J* = 10.3 Hz, 1H, H-1), 4.12–4.01 (m, 2H, H-3, H-4), 3.67 (d, *J* = 11.7 Hz, 1H, H-6b), 3.57–3.40 (m, 2H, H-2, H-6a), 3.31–3.19 (m, 1H, H-5), 3.16–3.02 (m, 2H, CH_2NC=O), 2.79–2.60 (m, 4H, 2 CH_2), 2.57 (t, *J* = 6.8 Hz, 2H, CH_2), 1.82–1.69 (m, 5H, NAc, CH_2). ^{13}C NMR (75 MHz, DMSO-d_6) δ 168.91, 163.38, 134.20, 131.22, 130.64, 127.28, 127.14, 122.01, 84.17, 81.12, 75.55, 70.47, 61.19, 54.59, 48.93, 46.46, 38.14, 29.70, 28.00, 23.03; HRMS (ESI) calcd for $C_{25}H_{32}N_3O_7S$ (M+H$^+$) 518.1961, found 518.1961.

*2-[4-[2-[(2-acetamido-β-D-glucopyranosyl)thio]ethylamino]butyl]-1H-benzo[de]isoquinoline-1,3(2H)-dione (**15c**)*

White solid; (0.49g, 91.7%) yield; $[\alpha]_D^{25}$ -25.9 (c=0.15, DMF); mp 236-237°C; ^{1}H NMR (300 MHz, DMSO-d_6) δ 8.44–8.30 (m, 4H, ArH), 7.86–7.68 (m, 3H, 2 ArH, NHAc), 5.17–4.92 (2 br s, 2H, 2 OH), 4.35 (d, *J* = 10.3 Hz, 1H, H-1), 4.05–3.91 (m, 2H, H-3, H-4), 3.67 (d, *J* = 11.6 Hz, 2H, H-6b), 3.60–3.50 (m, 3H, H-2, H-6a, OH), 3.28–3.20 (m, 1H, H-5), 3.15–3.02 (m, 2H, CH_2NC=O), 2.79–2.55 (m, 4H, 2 CH_2), 2.55–2.44 (m, 2H, CH_2), 1.77 (s, 3H, NAc), 1.69–1.53 (m, 2H, CH_2), 1.50–1.35 (m, 2H, CH_2); ^{13}C NMR (75 MHz, DMSO-d_6) δ 169.10, 163.50, 134.39, 131.39, 130.83, 127.44, 127.32, 122.13, 84.33, 81.26, 75.62, 70.62, 61.33, 54.67, 48.94, 48.42, 29.35, 26.83, 25.62, 24.98, 23.13; HRMS (ESI) calcd for $C_{26}H_{34}N_3O_7S$ (M+H$^+$) 532.2117, found 532.2118.

*2-[2-[3-[(2-acetamido-β-D-glucopyranosyl)thio]propylamino]ethyl]-1H-benzo[de]isoquinoline-1,3(2H)-dione (**15d**)*

White solid; (0.46g, 89.6%) yield; $[\alpha]_D^{25}$ -13.4 (c=0.10, DMF); mp 230-232°C; ^{1}H NMR (300 MHz, DMSO-d_6) δ 8.53–8.40 (m, 4H, ArH), 7.91–7.82 (m, 2H, ArH), 7.70 (d, *J* = 9.3 Hz, 1H, NHAc), 5.05–4.93 (m, 2H, 2 OH), 4.33 (d, *J* = 10.3 Hz, 1H, H-1), 4.18–4.08 (m, 2H, H-3, H-4), 3.65 (d, *J* = 11.6 Hz, 1H, H-6b), 3.52 (dd, *J* = 19.6, 9.8 Hz, 1H, H-2), 3.42 (dd, *J* = 11.6, 4.8 Hz, 1H, H-6a), 3.30–3.21 (m, 1H, H-5), 3.16–3.03 (m, 2H, CH_2NC=O), 2.77 (t, *J* = 6.9 Hz, 2H, CH_2), 2.70–2.56 (m, 4H, 2 CH_2), 1.80 (s, 3H, NAc), 1.71–1.57 (m, 2H, CH_2); ^{13}C NMR (75 MHz, DMSO-d_6) δ 169.04, 163.63, 134.32, 131.40, 130.77, 127.52, 127.30, 122.29, 84.35, 81.32, 75.70, 70.62, 61.32, 54.63, 47.85, 46.81, 39.40, 29.59, 27.39, 23.18; HRMS (ESI) calcd for $C_{25}H_{32}N_3O_7S$ (M+H$^+$) 518.1961, found 518.1960.

*2-[3-[3-[(2-acetamido-β-D-glucopyranosyl)thio]propylamino]propyl]-1H-benzo[de]isoquinoline-1,3(2H)-dione (**15e**)*

White solid; (0.46g, 86.8%) yield; $[\alpha]_D^{25}$ -43.2 (c=0.20, DMF); mp 212-214°C; ^{1}H NMR (300 MHz, DMSO-d_6) δ 8.42–8.28 (m, 4H, ArH), 7.84–7.68 (m, 3H, 2 ArH, NHAc), 5.03 (br s, 2H, 2 OH), 4.34 (d, J = 10.3 Hz, 1H, H-1), 4.08–3.96 (m, 2H, H-3, H-4), 3.68 (d, J = 11.6 Hz, 1H, H-6b), 3.54 (dd, J = 19.6, 9.8 Hz, 1H, H-2), 3.44 (dd, J = 11.2, 4.0 Hz, 1H, H-6a), 3.34–3.22 (m, 1H, H-5), 3.16–3.05 (m, 2H, CH_2NC=O), 2.70–2.58 (m, 2H, CH_2), 2.58–2.50 (m, 4H, 2 CH_2), 1.79 (s, 3H, NAc), 1.77–1.69 (m, 2H, CH_2), 1.67–1.55 (m, 2H, CH_2); ^{13}C NMR (75 MHz, DMSO-d_6) δ 169.14, 163.42, 134.26, 131.25, 130.69, 127.28, 127.18, 121.98, 84.38, 81.32, 75.70, 70.65, 61.37, 54.65, 48.12, 47.07, 38.24, 29.49, 28.09, 27.47, 23.17; HRMS (ESI) calcd for $C_{26}H_{34}N_3O_7S$ (M+H$^+$) 532.2117, found 532.2116.

*2-[4-[3-[(2-acetamido-β-D-glucopyranosyl)thio]propylamino]butyl]-1H-benzo[de]isoquinoline-1,3(2H)-dione (**15f**)*

White solid; (0.51g, 93.4%) yield; $[\alpha]_D^{25}$ -36.5 (c=0.10, DMF); mp196-198°C; ^{1}H NMR (300 MHz, DMSO-d_6) δ 8.45–8.31 (m, 4H, ArH), 7.86–7.69 (m, 3H, 2 ArH, NHAc), 5.31 (br s, 1H, OH), 5.07 (br s, 1H, OH), 4.37 (d, J = 10.3 Hz, 1H, H-1), 4.02–3.93 (m, 2H, H-3, H-4), 3.57–3.35 (m, 3H, H-6b, H-2, H-6a), 3.21–3.15 (m, 1H, H-5), 3.15–3.05 (m, 2H, CH_2NC=O), 2.68–2.48 (m, 6H, 3 CH_2), 1.78 (s, 3H, NAc), 1.70–1.55 (m, 4H, 2 CH_2), 1.51–1.33 (m, 2H, CH_2); ^{13}C NMR (75 MHz, DMSO-d_6) δ 169.19, 163.42, 134.31, 131.31, 130.75, 127.35, 127.23, 122.03, 84.47, 77.69, 75.34, 70.63, 63.88, 54.55, 49.10, 48.31, 39.69, 29.70, 27.58, 27.17, 25.66, 23.13; HRMS (ESI) calcd for $C_{27}H_{36}N_3O_7S$ (M+H$^+$) 546.2274, found 546.2264.

*2-[2-[5-[(2-acetamido-β-D-glucopyranosyl)thio]pentylamino]ethyl]-1H-benzo[de]isoquinoline-1,3(2H)-dione (**15g**)*

White solid; (0.50g, 91.6%) yield; $[\alpha]_D^{25}$ -23.2 (c=0.10, DMF); mp179-181°C; ^{1}H NMR (300 MHz, DMSO-d_6) δ 8.51–8.38 (m, 4H, ArH), 7.92–7.80 (m, 2H, ArH), 7.74 (d, J = 9.4 Hz, 1H, NHAc), 4.34 (d, J = 10.2 Hz, 1H, H-1), 4.18–4.07 (m, 2H, H-3, H-4), 3.70 (d, J = 11.5 Hz, 1H, H-6b), 3.63–3.53 (m, 2H, H-2, H-6a), 3.32–3.26 (m, 1H, H-5), 3.16–3.04 (m, 2H, CH_2NC=O), 2.79 (t, J = 6.8 Hz, 2H, CH_2), 2.65–2.53 (m, 4H, 2 CH_2), 1.82 (s, 3H, NAc), 1.59–1.42 (m, 2H, CH_2), 1.41–1.20 (m, 4H, 2 CH_2); ^{13}C NMR (75 MHz, DMSO-d_6) δ 169.15, 163.60, 134.28, 131.33, 130.74, 127.43, 127.25, 122.18, 84.26, 81.26, 75.66, 70.66, 61.34, 54.68, 48.90, 46.88, 39.89, 39.48, 29.19, 29.14, 26.22, 23.16; HRMS (ESI) calcd for $C_{27}H_{36}N_3O_7S$ (M+H$^+$) 546.2274, found 546.2272.

*2-[3-[5-[(2-acetamido-β-D-glucopyranosyl)thio]pentylamino]propyl]-1H-benzo[de]isoquinoline-1,3(2H)-dione (**15h**)*

White solid; (0.51g, 91.1%) yield; $[\alpha]_D^{25}$ -41.4 (c=0.15, DMF); mp172-174°C; ^{1}H NMR (300 MHz, DMSO-d_6) δ 8.46–8.37 (m, 4H, ArH), 7.91–7.77 (m, 3H, 2 ArH, NHAc), 5.12 (br s, 1H, 2 OH), 4.33 (d, J = 10.2 Hz, 1H, H-1), 4.13–4.00 (m, 2H, H-3, H-4), 3.64 (d, J = 11.6 Hz, 1H, H-6b), 3.58–3.48 (m, 2H, H-2, H-6a), 3.34–3.27 (m, 1H, H-5), 3.12–3.04 (m, 2H, CH_2NC=O), 3.01–2.87 (m, 2H, CH_2), 2.85–2.71 (m, 2H, CH_2), 2.65–2.52 (m, 2H, CH_2), 2.15–1.96 (m, 2H, CH_2), 1.78 (s, 3H, NAc), 1.69–1.55 (m, 2H, CH_2), 1.55–1.42 (m, 2H, CH_2), 1.40–1.25 (m, 2H, CH_2); ^{13}C NMR

(75 MHz, DMSO-d_6) δ 169.20, 163.70, 134.49, 131.36, 130.84, 127.48, 127.30, 122.07, 84.34, 81.18, 75.53, 70.67, 61.24, 54.54, 46.65, 44.93, 37.32, 29.08, 28.69, 25.34, 24.64, 24.21, 23.17; HRMS (ESI) calcd for $C_{28}H_{38}N_3O_7S$ (M+H$^+$) 560.2430, found 560.2433.

*2-[4-[5-[(2-acetamido-β-D-glucopyranosyl)thio]pentylamino]butyl]-1H-benzo[de]isoquinoline-1,3(2H)-dione (**15i**)*

White solid; (0.51g, 88.9%) yield; $[\alpha]_D^{25}$ -20.8 (c=0.15, DMF); mp177-179°C; ^{1}H NMR (300 MHz, DMSO-d_6) δ 8.49–8.37 (m, 4H, ArH), 7.88–7.79 (m, 2H, ArH), 7.71 (d, *J* = 9.3 Hz, 1H, NHAc), 4.99 (br s, 2H, 2 OH), 4.31 (d, *J* = 10.3 Hz, 1H, H-1), 4.09–3.95 (m, 2H, H-3, H-4), 3.67 (d, *J* = 11.5 Hz, 1H, H-6b), 3.57–3.46 (m, 2H, H-2, H-6a), 3.28–3.24 (m, 1H, H-5), 3.11–3.03 (m, 2H, CH_2NC=O), 2.62–2.50 (m, 4H, 2 CH_2), 2.44 (t, *J* = 6.6 Hz, 2H, CH_2), 1.78 (s, 3H, NAc), 1.73–1.57 (m, 2H, CH_2), 1.55–1.40 (m, 4H, 2 CH_2), 1.39–1.21 (m, 4H, 2 CH_2); ^{13}C NMR (75 MHz, DMSO-d_6) δ 169.10, 163.47, 134.36, 131.37, 130.80, 127.42, 127.28, 122.10, 84.25, 81.28, 79.27, 75.65, 70.65, 61.32, 54.66, 49.26, 49.12, 29.16, 29.13, 28.99, 27.04, 26.22, 25.64, 23.16; HRMS (ESI) calcd for $C_{29}H_{40}N_3O_7S$ (M+H$^+$) 574.2587, found 574.2584.

*2-[2-[6-[(2-acetamido-β-D-glucopyranosyl)thio]hexylamino]ethyl]-1H-benzo[de]isoquinoline-1,3(2H)-dione (**15j**)*

Light yellow solid; (0.53g, 94.7%) yield; $[\alpha]_D^{25}$ -17.9 (c=0.10, DMF); mp196-198°C; ^{1}H NMR (300 MHz, DMSO-d_6) δ 8.43–8.31 (m, 4H, ArH), 7.84–7.75 (m, 2H, ArH), 7.71 (d, *J* = 9.3 Hz, 1H, NHAc), 4.32 (d, *J* = 10.3 Hz, 1H, H-1), 4.13–4.01 (m, 2H, H-3, H-4), 3.68 (d, *J* = 11.5 Hz, 1H, H-6b), 3.49 (dd, *J* = 17.0, 7.3 Hz, 1H, H-2), 3.44 (dd, *J* = 11.8, 4.4 Hz, 1H, H-6a), 3.34–3.23 (m, 1H, H-5), 3.13–3.04 (m, 2H, CH_2NC=O), 2.75 (t, *J* = 6.8 Hz, 2H, CH_2), 2.60–2.44 (m, 4H, 2 CH_2), 1.78 (s, 3H, NAc), 1.54–1.38 (m, 2H, CH_2), 1.38–1.29 (m, 2H, CH_2), 1.29–1.08 (m, 4H, 2 CH_2); ^{13}C NMR (75 MHz, DMSO-d_6) δ 169.05, 163.52, 134.24, 131.29, 130.68, 127.38, 127.20, 122.13, 84.27, 81.31, 75.69, 70.68, 61.35, 54.68, 49.02, 46.90, 39.54, 29.64, 29.17, 28.43, 26.51, 23.19; HRMS (ESI) calcd for $C_{28}H_{38}N_3O_7S$ (M+H$^+$) 560.2430, found 560.2436.

*2-[3-[6-[(2-acetamidoβ-D-glucopyranosyl)thio]hexylamino]propyl]-1H-benzo[de]isoquinoline-1,3(2H)-dione (**15k**)*

White solid; (0.50g, 87.1%) yield; $[\alpha]_D^{25}$ -33.5 (c=0.15, DMF); mp200-202°C; ^{1}H NMR (300 MHz, DMSO-d_6) δ 8.42 (ddd, *J* = 9.3, 7.8, 1.0 Hz, 4H, ArH), 7.82 (dd, *J* = 8.2, 7.4 Hz, 2H, ArH), 7.71 (d, *J* = 9.4 Hz, 1H, NHAc), 5.03 (br s, 2H, 2 OH), 4.32 (d, *J* = 10.3 Hz, 1H, H-1), 4.11–4.00 (m, 2H, H-3, H-4), 3.68 (d, *J* = 11.5 Hz, 1H, H-6b), 3.48 (dd, *J* = 17.8, 7.8 Hz, 1H, H-2), 3.43 (dd, *J* = 12.1, 5.0 Hz, 1H, H-6a), 3.31–3.21 (m, 1H, H-5), 3.13–3.04 (m, 2H, CH_2NC=O), 2.62–2.50 (m, 4H, 2 CH_2), 2.42 (t, *J* = 6.8 Hz, 2H, CH_2), 1.83–1.69 (m, 5H, NAc, CH_2), 1.53–1.38 (m, 2H, CH_2), 1.36–1.15 (m, 6H, 3 CH_2); ^{13}C NMR (75 MHz, DMSO-d_6) δ 169.03, 163.52, 134.33, 131.36, 130.76, 127.41, 127.27, 122.13, 84.25, 81.32, 75.69, 70.66, 61.34, 54.67, 49.35, 47.21, 38.31, 29.61, 29.16, 28.43, 28.05, 26.55, 23.19; HRMS (ESI) calcd for $C_{29}H_{40}N_3O_7S$ (M+H$^+$) 574.2587, found 574.2584.

*2-[4-[6-[(2-acetamido-β-D-glucopyranosyl)thio]hexylamino]butyl]-1H-benzo[de]isoquinoline-1,3(2H)-dione (**15l**)*

Light yellow solid; (0.52g, 88.5%) yield; $[\alpha]_D^{25}$ -41.2 (c=0.15, DMF); mp143-145°C; ^{1}H NMR (300 MHz, DMSO-d_6) δ 8.47–8.31 (m, 4H, ArH), 7.88–7.77 (m, 2H, ArH), 7.73 (d, *J* = 9.3 Hz, 1H, NHAc), 4.31 (d, *J* = 10.3 Hz, 1H, H-1), 4.05–3.94 (m, 2H, H-3, H-4), 3.67 (d, *J* = 11.6 Hz, 1H, H-6b), 3.57–3.41 (m, 2H, H-2, H-6a), 3.33–3.21 (m, 1H, H-5), 3.12–3.01 (m, 2H, CH_2NC=O), 2.62–2.49 (m, 4H, 2 CH_2), 2.41 (t, *J* = 6.8 Hz, 2H, CH_2), 1.79 (s, 3H, NAc), 1.69–1.54 (m, 2H, CH_2), 1.52–1.37 (m, 4H, 2 CH_2), 1.36–1.15 (m, 6H, 3 CH_2); ^{13}C NMR (75 MHz, DMSO-d_6) δ 169.06, 163.43, 134.33, 131.34, 130.76, 127.38, 127.25, 122.07, 84.27, 81.31, 75.68, 70.67, 61.34, 54.67, 49.42, 49.21, 48.72, 39.71, 29.49, 29.16, 28.40, 27.15, 26.54, 25.65, 23.16; HRMS (ESI) calcd for $C_{30}H_{42}N_3O_7S$ (M+H$^+$) 588.2743, found 588.2744.

General synthetic procedure for compounds 19a-19e

Compound **18** (10 mmol, 1.0 eq) was first dissolved in acetonitrile (20 mL). Anhydrous potassium carbonate (12 mmol, 1.2 eq) and α, ω- dibromoalkane (40 mmol, 4.0 eq) were then added. The mixture was refluxed for 12 h, until TLC (P/E=3:1) indicated that the reaction was complete. After undissolved substance was removed by filtration, the remaining solvent was evaporated under reduced pressure to get crude product, which was further purified by silica gel column chromatography using P/E (10:1) and compounds **19a-19e** were obtained.

*2-(2-Bromoethyl)-1H-benzo[de]isoquinoline-1,3(2H)-dione (***19a***)*

White solid; (2.6g, 85.6%) yield; mp 99-101°C [Lit. mp 98-100°C][5]; ^{1}H NMR (300 MHz, $CDCl_3$) δ 8.62 (dd, *J* = 7.3, 1.1 Hz, 2H, Ar-H), 8.24 (dd, *J* = 8.4, 1.0 Hz, 2H, Ar-H), 7.77 (dd, *J* = 8.2, 7.3 Hz, 2H, Ar-H), 4.62 (t, *J* = 7.2 Hz, 2H, CH_2), 3.68 (t, *J* = 7.2 Hz, 2H, CH_2).

2-(3-bromopropyl)-1H-benzo[de]isoquinoline-1,3(2H)-dione (**19b**)

White solid; (2.6g, 81.7%) yield; mp101-103°C [Lit. mp 99-101°C][5]; ^{1}H NMR (300 MHz, DMSO-d6) δ 8.36-8.31 (m, 4H, Ar-H), 7.75 (t, *J* = 7.8 Hz, 2H, Ar-H), 4.09 (t, *J* = 7.0 Hz, 2H, CH_2), 3.59 (t, *J* = 6.7 Hz, 2H, CH_2), 2.24–2.08 (m, 2H, CH_2).

2-(4-bromobutyl)-1H-benzo[de]isoquinoline-1,3(2H)-dione (**19c**)

White solid; (2.8g, 84.5%) yield; mp102-104°C [Lit. mp 100-103°C][5]; ^{1}HNMR (300MHz, DMSO-d_6) δ 8.49–8.39 (m, 4H, Ar-H), 7.82 (dd, *J* = 8.1, 7.5 Hz, 2H, Ar-H), 4.21 (t, *J* = 7.0 Hz, 2H, CH_2), 3.48 (t, *J* = 6.8 Hz, 2H, CH_2), 2.01–1.85 (m, 4H, 2 CH_2).

2-(5-bromopentyl)-1H-benzo[de]isoquinoline-1,3(2H)-dione (***19d***)

White solid; (2.9g, 83.8%) yield; mp119-120ºC [Lit. mp 118-120ºC][5]; ^{1}H NMR (300 MHz, DMSO-d_6) δ 8.52–8.40 (m, 4H, Ar-H), 7.84 (dd, J = 8.1, 7.5 Hz, 2H, Ar-H), 4.08–3.97 (m, 2H, CH_2), 3.53 (t, J = 6.7 Hz, 2H, CH_2), 1.91–1.79 (m, 2H, CH_2), 1.70–1.60 (m, 2H, CH_2), 1.49–1.39 (m, 2H, CH_2); ^{13}C NMR (75 MHz, DMSO-d_6) δ 163.51, 134.40, 131.40, 130.83, 127.46, 127.32, 122.14, 39.50, 35.02, 32.04, 26.74, 25.16.

2-(6-bromohexyl)-1H-benzo[de]isoquinoline-1,3(2H)-dione (***19e***)

White solid; (2.9g, 80.5%) yield; mp 93-95ºC [Lit. mp 90-94ºC][5]; ^{1}H NMR (300 MHz, DMSO-d6) δ 8.48–8.32 (m, 4H, Ar-H), 7.81 (dd, J = 8.1, 7.4 Hz, 2H, Ar-H), 4.08–3.92 (m, 2H, CH_2), 3.50 (t, J = 6.7 Hz, 2H, CH_2), 1.85–1.72 (m, 2H, CH_2), 1.68–1.52 (m, 2H, CH_2), 1.50–1.27 (m, 4H, 2 CH_2);^{13}C NMR (75 MHz, DMSO-d6) δ 163.43, 134.32, 131.34, 130.75, 127.40, 127.24, 122.10, 39.63, 35.15, 32.25, 27.43, 27.39, 25.77.

Characterization of compound 20a-20e

*2-[2-[(2-acetamido-3,4,6-tri-O-acetyl-β-D-glucopyranosyl)thio]ethyl]-1H-benzo[de]isoquinoline-1,3(2H)-dione (***20a***)*

White solid; (1.35g, 82.3%) yield; $[\alpha]_D^{25}$ -62.1(c=1.0, DMF); mp200-202ºC; ^{1}H NMR (300 MHz, $CDCl_3$) δ 8.60 (d, J = 7.3 Hz, 2H, Ar–H), 8.25 (d, J = 8.2 Hz, 2H, Ar–H), 7.78 (t, J = 7.8 Hz, 2H, Ar–H), 5.80 (d, J = 9.5 Hz, 1H, NH), 5.27–5.12 (m, 2H, H-3, H-4), 4.90 (d, J = 10.5 Hz, 1H, H-1), 4.60 (ddd, J = 13.3, 9.1, 6.8 Hz, 1H, CH_2N), 4.40–4.11 (m, 4H, CH_2N, H-6b, H-6a, H-2), 3.83 (ddd, J = 9.3, 4.4, 2.2 Hz, 1H, H-5), 3.25–2.96 (m, 2H, SCH_2), 2.07, 2.05, 2.04 (3 s, 9H, 3 OAc), 1.78 (s, 3H, NAc);^{13}C NMR (75 MHz, DMSO-d_6) δ 170.12, 169.76, 169.40, 169.22, 163.38, 134.51, 131.44, 130.88, 127.55, 127.33, 122.16, 83.96, 75.02, 73.75, 68.69, 62.05, 52.17, 39.97, 27.55, 22.71, 20.55, 20.51, 20.47; HRMS (ESI) calcd for $C_{28}H_{31}N_2O_{10}S$ ($M+H^+$) 587.1699, found 587.1709.

*2-[3-[(2-acetamido-3,4,6-tri-O-acetyl-β-D-glucopyranosyl)thio]propyl]-1H-benzo[de]isoquinoline-1,3(2H)-dione (***20b***)*

White solid; (1.40g, 83.5%) yield; $[\alpha]_D^{25}$ -57.8(c=1.0, DMF); mp260-262ºC; ^{1}H NMR (300 MHz, DMSO-d_6) δ 8.46–8.34 (m, 4H, ArH), 7.96 (d, J = 9.4 Hz, 1H, NH), 7.81 (t, J = 7.8 Hz, 2H, ArH), 5.08 (t, J = 9.7 Hz, 1H, H-3), 4.84 (t, J = 9.7 Hz, 1H, H-4), 4.73 (d, J = 10.4 Hz, 1H, H-1), 4.16–4.04 (m, 3H, H-6b, H-2, H-6a), 4.03–3.95 (m, 1H, CH_2N), 3.92–3.78 (m, 2H, CH_2N, H-5), 2.84–2.57 (m, 2H, SCH_2), 1.97, 1.95, 1.91 (3 s, 9H, 3 OAc), 1.95–1.86 (m, 2H, CH_2), 1.76 (s, 3H, NAc); ^{13}C NMR (75 MHz, DMSO-d_6) δ 170.11, 169.76, 169.41, 169.27, 163.53, 134.33, 131.33, 130.75, 127.42, 127.24, 122.11, 83.69, 74.72, 73.79, 68.72, 62.14, 52.31, 39.10, 28.37, 27.45, 22.73, 20.51, 20.44; HRMS (ESI) calcd for $C_{29}H_{33}N_2O_{10}S$ ($M+H^+$) 601.1856, found 601.1850.

*2-[4-[(2-acetamido-3,4,6-tri-O-acetyl-β-D-glucopyranosyl)thio]butyl]-1H-benzo[de]isoquinoline-1,3(2H)-dione (***20c***)*

White solid; (1.39g, 80.9%) yield; $[\alpha]_D^{25}$ -55.2(c=1.0, DMF); mp211-213°C; ^{1}H NMR (300 MHz, DMSO-d_6) δ 8.42–8.31 (m, 4H, ArH), 7.98 (d, *J* = 9.5 Hz, 1H, NH), 7.83–7.74 (m, 2H, ArH), 5.08 (t, *J* = 9.7 Hz, 1H, H-3), 4.83 (t, *J* = 9.7 Hz, 1H, H-4), 4.70 (d, *J* = 10.4 Hz, 1H, H-1), 4.12 (dd, *J* = 12.3, 5.0 Hz, 1H, H-6b), 4.05–3.92 (m, 3H, H-6a, H-2, CH_2N), 3.92–3.78 (m, 2H, CH_2N, H-5), 2.76–2.57 (m, 2H, SCH_2), 1.97, 1.96, 1.91 (3 s, 9H, 3 OAc), 1.76 (s, 3H, NAc), 1.72–1.55 (m, 4H, 2 CH_2); ^{13}C NMR (75 MHz, DMSO-d_6) δ 170.10, 169.76, 169.40, 169.26, 163.42, 134.29, 131.29, 130.72, 127.34, 127.19, 122.02, 83.81, 74.70, 73.83, 68.72, 62.12, 52.34, 39.25, 29.40, 27.33, 26.93, 22.72, 20.54, 20.50, 20.43; HRMS (ESI) calcd for $C_{30}H_{35}N_2O_{10}S$ (M+H$^+$) 615.2012, found 615.2021.

*2-[5-[(2-acetamido-3,4,6-tri-O-acetyl-β-D-glucopyranosyl)thio]pentyl]-1H-benzo[de]isoquinoline-1,3(2H)-dione (***20d***)*

White solid; (1.50g, 85.2%) yield; $[\alpha]_D^{25}$ -44.9(c=1.0, DMF); mp179-181°C; ^{1}H NMR (300 MHz, DMSO-d_6) δ 8.38 (m, 4H, Ar-H), 7.98 (d, *J* = 9.4 Hz, 1H, NH), 7.85–7.75 (m, 2H, Ar-H), 5.08 (t, *J* = 9.8 Hz, 1H, H-3), 4.84 (t, *J* = 9.7 Hz, 1H, H-4), 4.69 (d, *J* = 10.4 Hz, 1H, H-1), 4.13 (dd, *J* = 12.3, 5.1 Hz, 1H, H-6b), 4.05–3.95 (m, 3H, H-6a, H-2, CH_2N), 3.92–3.78 (m, 2H, CH_2N, H-5), 2.78–2.52 (m, 2H, SCH_2), 1.97, 1.95, 1.91 (3 s, 9H, 3 OAc), 1.75 (s, 3H, NAc), 1.69–1.51 (m, 4H, 2 CH_2), 1.46–1.31 (m, 2H, CH_2); ^{13}C NMR (75 MHz, DMSO-d_6) δ 170.07, 169.75, 169.38, 169.19, 163.39, 134.30, 131.31, 130.73, 127.35, 127.22, 122.04, 83.54, 74.73, 73.84, 68.74, 62.18, 52.32, 39.95, 29.24, 29.16, 27.14, 25.78, 22.71, 20.52, 20.45; HRMS (ESI) calcd for $C_{31}H_{37}N_2O_{10}S$ (M+H$^+$) 629.2169, found 629.2174.

*2-[6-[(2-acetamido-3,4,6-tri-O-acetyl-β-D-glucopyranosyl)thio]hexyl]-1H-benzo[de]isoquinoline-1,3(2H)-dione (***20e***)*

White solid; (1.51g, 83.9%) yield; $[\alpha]_D^{25}$ -41.8(c=1.0, DMF); mp222-224°C; ^{1}H NMR (300 MHz, DMSO-d_6) δ 8.52–8.42 (m, 4H, ArH), 7.96 (d, *J* = 9.5 Hz, 1H, NH), 7.90–7.82 (m, 2H, ArH), 5.06 (t, J = 9.7 Hz, 1H, H-3), 4.82 (t, J = 9.7 Hz, 1H, H-4), 4.68 (d, J = 10.4 Hz, 1H, H-1), 4.13 (dd, J = 12.3, 5.1 Hz, 1H, H-6b), 4.07–3.95 (m, 3H, H-2, CH_2N, H-6a), 3.90–3.76 (m, 2H, CH_2N, H-5), 2.72–2.52 (m, 2H, SCH_2), 1.98, 1.97, 1.96 (3 s, 9H, 3 OAc), 1.91 (s, 3H, NAc), 1.69–1.48 (m, 4H, 2 CH_2), 1.45–1.27 (m, 4H, 2 CH_2); ^{13}C NMR (75 MHz, DMSO-d_6) δ 170.08, 169.75, 169.39, 169.17, 163.51, 134.41, 131.42, 130.83, 127.48, 127.33, 122.19, 83.53, 74.69, 73.83, 68.72, 62.15, 52.31, 39.94, 29.37, 29.19, 27.95, 27.48, 26.13, 22.73, 20.57, 20.53, 20.45; HRMS (ESI) calcd for $C_{32}H_{39}N_2O_{10}S$ (M+H$^+$) 643.2325, found 643.2317.

Characterization of compound 21b-21e

*2-[3-[(2-acetamido-β-D-glucopyranosyl)thio]propyl]-1H-benzo[de]isoquinoline-1,3(2H)-dione (**21b**)*

White solid; (0.42g, 89.4%) yield; $[\alpha]_D^{25}$ -8.6(c=0.2, DMF); mp280-282°C; ^{1}H NMR (300 MHz, DMSO-d_6) δ 8.39 (t, *J* = 8.1 Hz, 4H, ArH), 7.80 (t, *J* = 7.8 Hz, 2H, ArH), 7.69 (d, *J* = 9.3 Hz, 1H, NHAc), 4.99 (d, *J* = 4.1 Hz, 1H, OH), 4.95 (d, *J* = 5.3 Hz, 1H, OH), 4.46 (t, *J* = 5.8 Hz, 1H, OH), 4.38 (d, *J* = 10.3 Hz, 1H, H-1), 4.13–3.98 (m, 2H, H-3, H-4), 3.64 (dd, *J* = 11.4, 6.0 Hz, 1H, H-6b), 3.54 (dd, *J* = 19.5, 9.8 Hz, 1H, H-2), 3.42 (dd, *J* = 11.6, 5.6 Hz, 1H, H-6a), 3.33–3.22 (m, 1H, H-5), 3.17–3.04 (m, 2H, CH_2N), 2.79–2.61 (m, 2H, SCH_2), 1.99–1.82 (m, 2H, CH_2), 1.79 (s, 3H, NAc); ^{13}C NMR (75 MHz, DMSO-d_6) δ 169.13, 163.49, 134.32, 131.30, 130.73, 127.38, 127.22, 122.07, 84.35, 81.26, 75.66, 70.57, 61.26, 54.63, 39.27, 28.03, 27.12, 23.16; HRMS (ESI) calcd for $C_{23}H_{27}N_2O_7S$ (M+H$^+$) 475.1539, found 475.1534.

*2-[4-[(2-acetamido-β-D-glucopyranosyl)thio]butyl]-1H-benzo[de]isoquinoline-1,3(2H)-dione (**21c**)*

White solid; (0.45g, 91.8%) yield; $[\alpha]_D^{25}$ -21.8(c=0.2, DMF); mp256-258°C; ^{1}H NMR (300 MHz, DMSO-d_6) δ 8.43–8.33 (m, 4H, ArH), 7.84–7.76 (m, 2H, ArH), 7.70 (d, *J* = 9.3 Hz, 1H, NAc), 5.01 (d, *J* = 4.7 Hz, 1H, OH), 4.96 (d, *J* = 5.4 Hz, 1H, OH), 4.49 (t, *J* = 5.8 Hz, 1H, OH), 4.35 (d, *J* = 10.3 Hz, 1H, H-1), 4.05–3.95 (m, 2H, H-3, H-4), 3.66 (dd, *J* = 11.5, 5.9 Hz, 1H, H-6b), 3.52 (dd, *J* = 19.6, 9.8 Hz, 1H, H-2), 3.46–3.39 (m, 1H, H-6a), 3.32–3.22 (m, 1H, H-5), 3.16–3.05 (m, 2H, NCH_2), 2.74–2.60 (m, 2H, SCH_2), 1.78 (s, 3H, NAc), 1.74–1.49 (m, 4H, 2 CH_2); ^{13}C NMR (75 MHz, DMSO-d_6) δ 169.10, 163.44, 134.33, 131.32, 130.75, 127.37, 127.24, 122.04, 84.38, 81.25, 75.68, 70.62, 61.32, 54.66, 39.38, 29.06, 27.08, 23.16; HRMS (ESI) calcd for $C_{24}H_{29}N_2O_7S$ (M+H$^+$) 489.1695, found 489.1698.

*2-[5-[(2-acetamido-β-D-glucopyranosyl)thio]pentyl]-1H-benzo[de]isoquinoline-1,3(2H)-dione (**21d**)*

White solid; (0.45g, 90.0%) yield; $[\alpha]_D^{25}$ -17.4(c=0.2, DMF); mp228-230°C; ^{1}H NMR (300 MHz, DMSO-d_6) δ 8.43–8.31 (m, 4H, ArH), 7.83–7.75 (m, 2H, ArH), 7.72 (d, *J* = 9.2 Hz, 1H, NHAc), 5.12–4.92 (m, 2H, 2 OH), 4.51 (br s, 1H, OH), 4.34 (d, *J* = 10.3 Hz, 1H, H-1), 4.04–3.89 (m, 2H, H-3, H-4), 3.68 (d, *J* = 11.2 Hz, 1H, H-6b), 3.59–3.43 (m, 2H, H-2, H-6a), 3.33–3.21 (m, 1H, H-5), 3.13–3.00 (m, 2H, NCH_2), 2.68–2.54 (m, 2H, SCH_2), 1.78 (s, 3H, NAc), 1.68–1.47 (m, 4H, 2 CH_2), 1.45–1.28 (m, 2H, CH_2); ^{13}C NMR (75 MHz, DMSO-d_6) δ 169.11, 163.37, 134.30, 131.28, 130.73, 127.31, 127.21, 121.99, 84.25, 81.28, 75.70, 70.65, 61.34, 54.67, 48.74, 29.12, 28.95, 27.24, 26.00, 23.13; HRMS (ESI) calcd for $C_{25}H_{31}N_2O_7S$ (M+H$^+$) 503.1852, found 503.1852.

*2-[6-[(2-acetamido-β-D-glucopyranosyl)thio]hexyl]-1H-benzo[de]isoquinoline-1,3(2H)-dione (**21e**)*

White solid; (0.46g, 88.5%) yield; $[\alpha]_D^{25}$ -25.6(c=0.2, DMF); mp241-243°C; ^{1}H NMR (300 MHz, DMSO-d_6) δ 8.50–8.39 (m, 4H, ArH), 7.89–7.80 (m, 2H, ArH), 7.69 (d, *J* = 9.4 Hz, 1H,

NHAc), 5.00 (d, *J* = 4.6 Hz, 1H, OH), 4.95 (d, *J* = 5.4 Hz, 1H, OH), 4.50 (t, *J* = 5.8 Hz, 1H, OH), 4.32 (d, *J* = 10.3 Hz, 1H, H-1), 4.07–3.92 (m, 2H, H-3, H-4), 3.66 (dd, *J* = 11.4, 5.9 Hz, 1H, H-6b), 3.51 (dd, *J* = 19.6, 9.8 Hz, 1H, H-2), 3.42 (dd, *J* = 11.4, 5.5 Hz, 1H, H-6a), 3.31–3.20 (m, 1H, H-5), 3.12–3.01 (m, 2H, NCH_2), 2.69–2.53 (m, 2H, SCH_2), 1.78 (s, 3H, NAc), 1.73–1.44 (m, 4H, 2 CH_2), 1.43–1.23 (m, 4H, 2 CH_2); ^{13}C NMR (75 MHz, DMSO-d_6) δ 169.04, 163.39, 134.31, 131.32, 130.74, 127.36, 127.23, 122.06, 84.22, 81.30, 75.71, 70.65, 61.34, 54.69, 39.95, 29.17, 29.04, 28.17, 27.51, 26.26, 23.18; HRMS (ESI) calcd for $C_{26}H_{33}N_2O_7S$ $(M+H^+)$ 517.2008, found 517.2009.

^1H NMR spectrum of compound 10

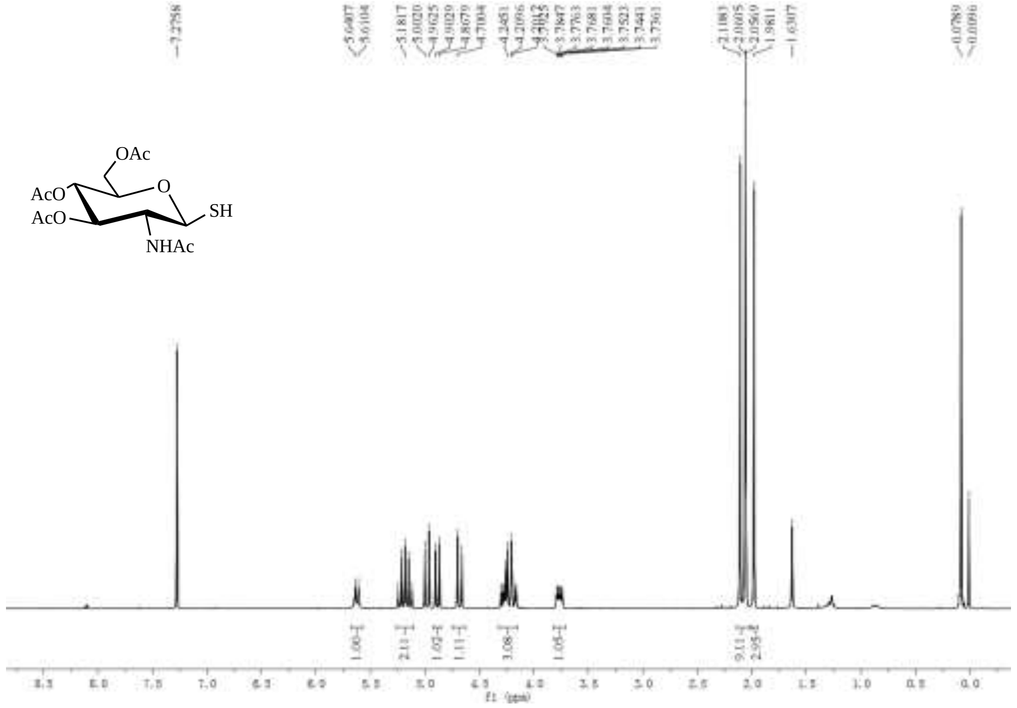

^1H NMR spectrum of compound 11a

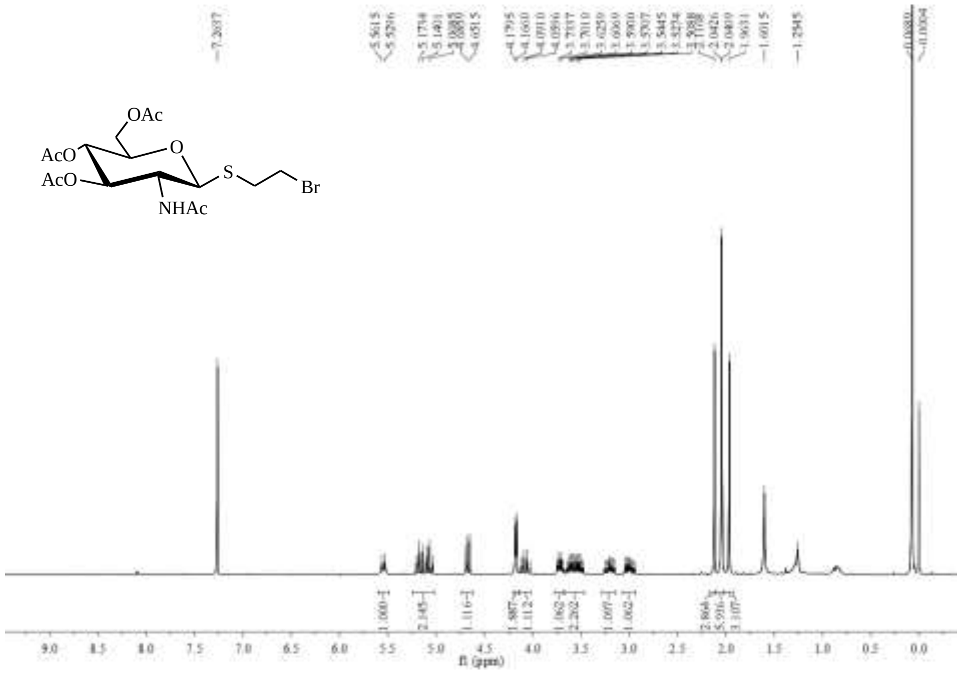

^{1}H NMR spectrum of compound 11b

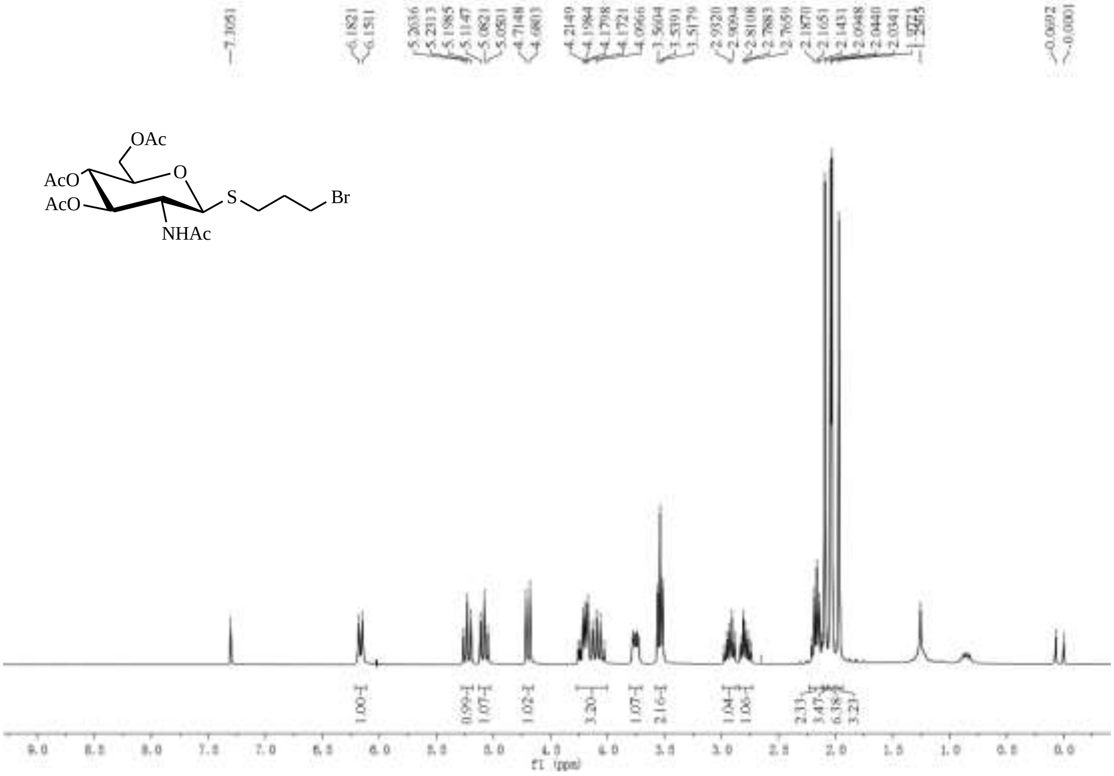

^{13}C NMR spectrum of compound 11b

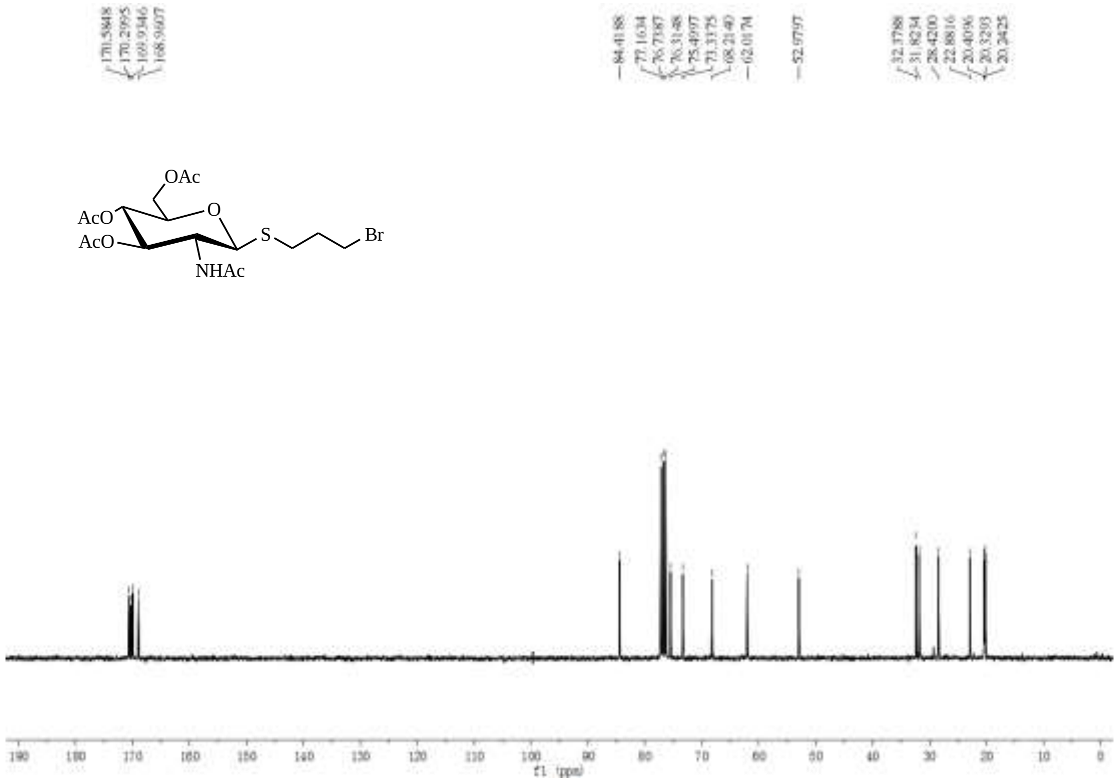

^{1}H NMR spectrum of compound 11c

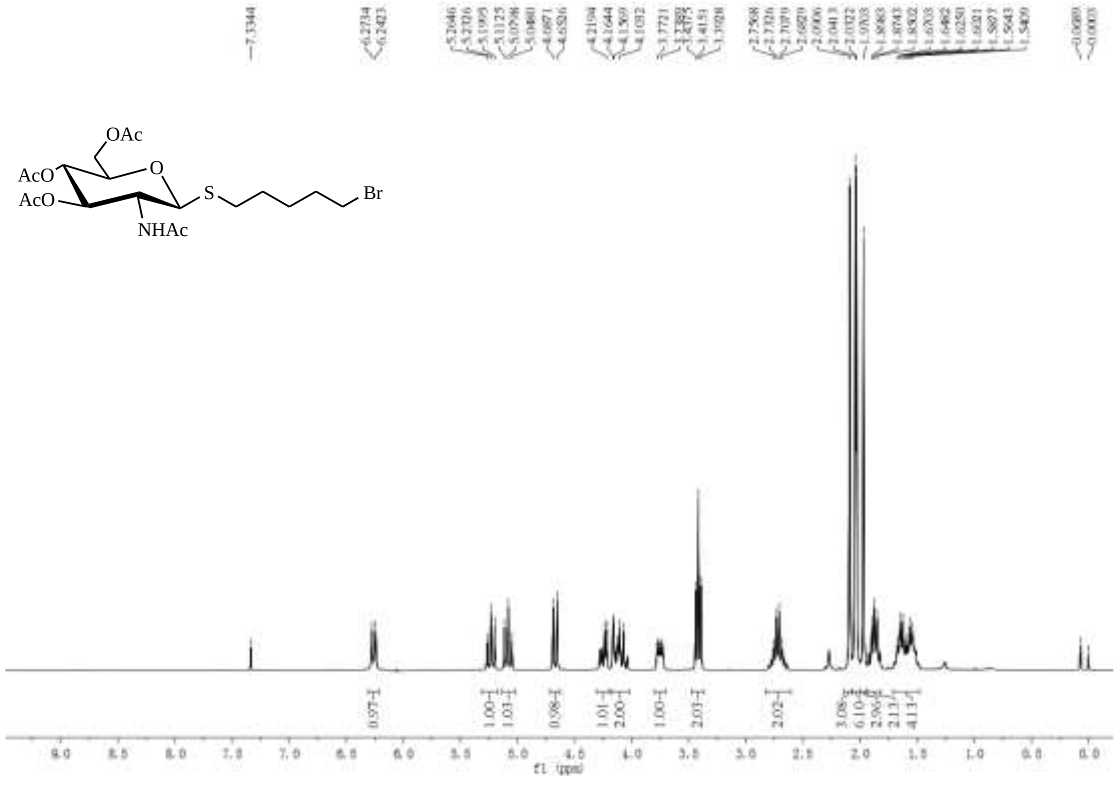

^{13}C NMR spectrum of compound 11c

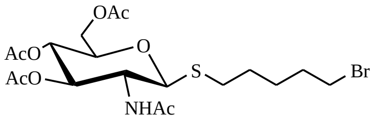

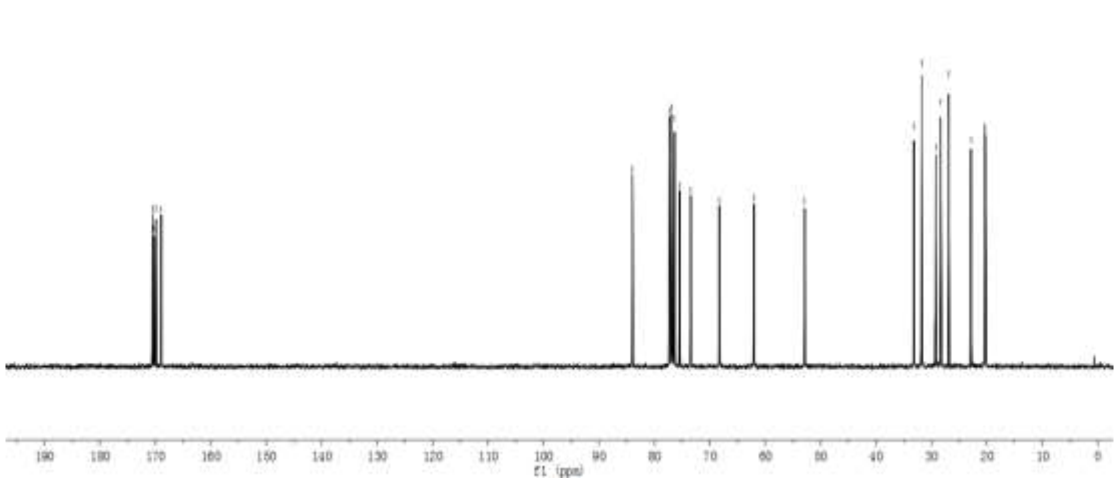

^{1}H NMR spectrum of compound 11d

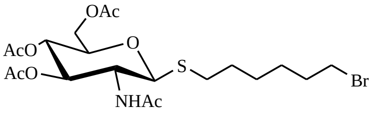

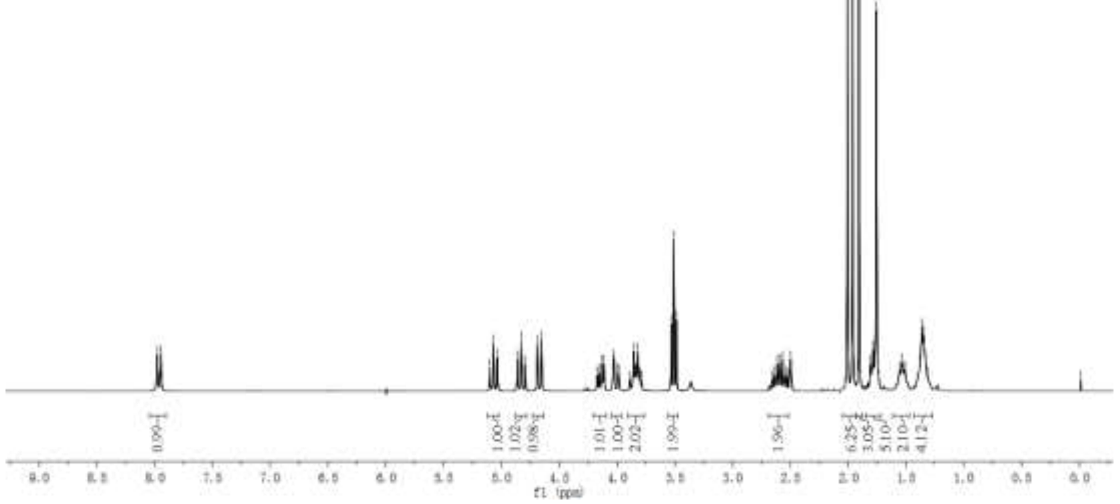

^{13}C NMR spectrum of compound 11d

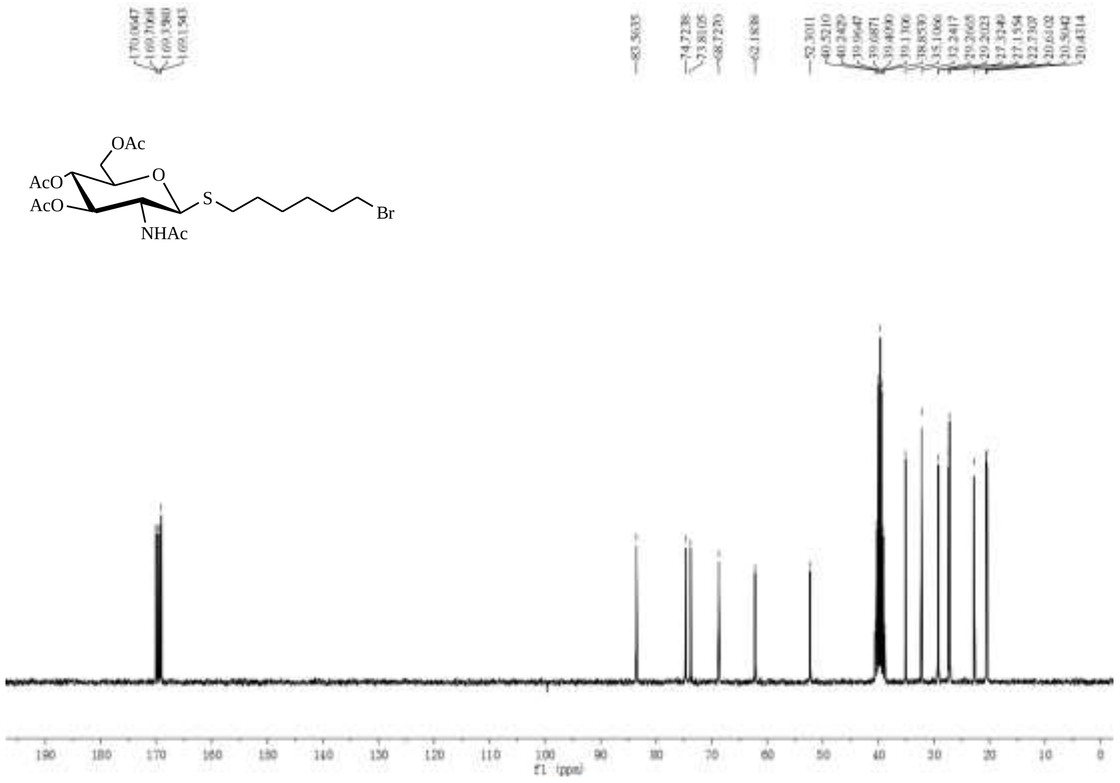

^{1}H NMR spectrum of compound 11e

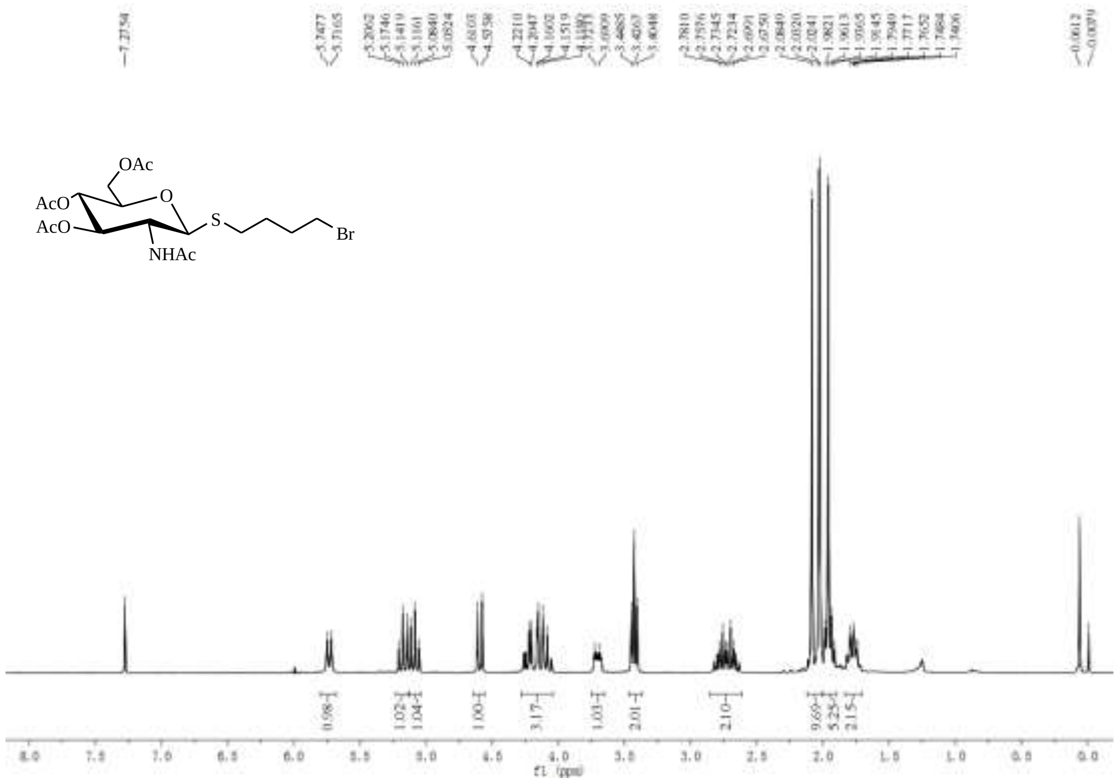

^{1}H NMR spectrum of compound 16

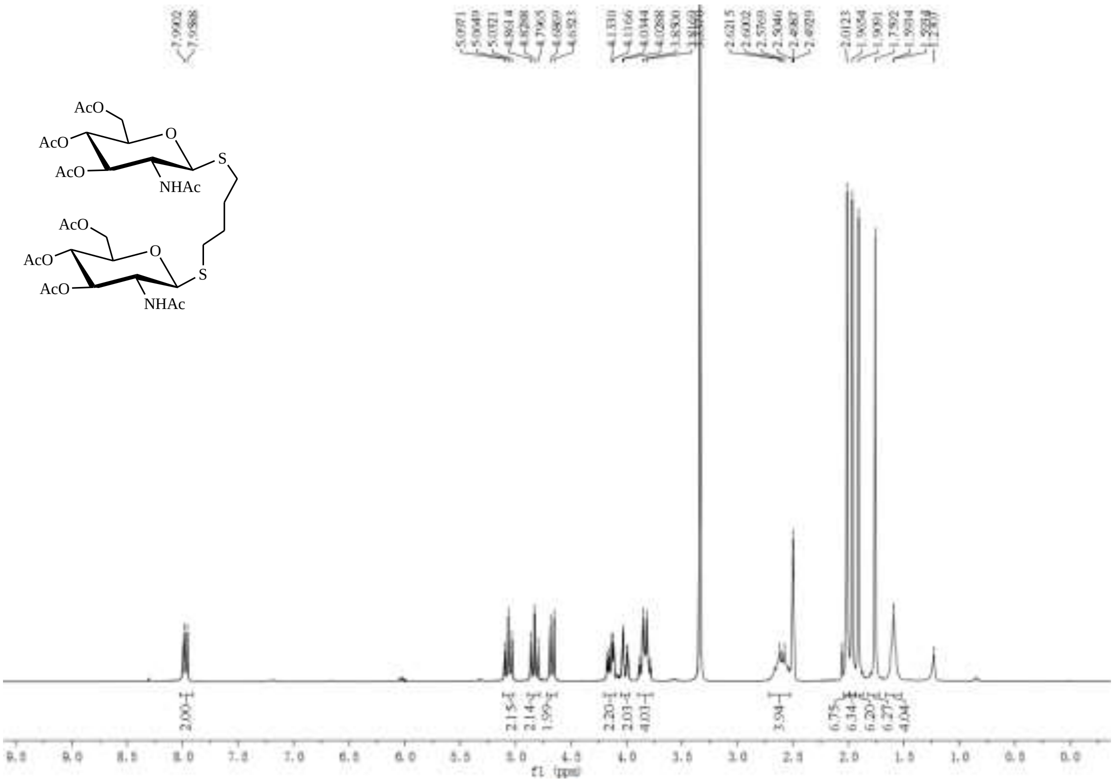

^{13}C NMR spectrum of compound 16

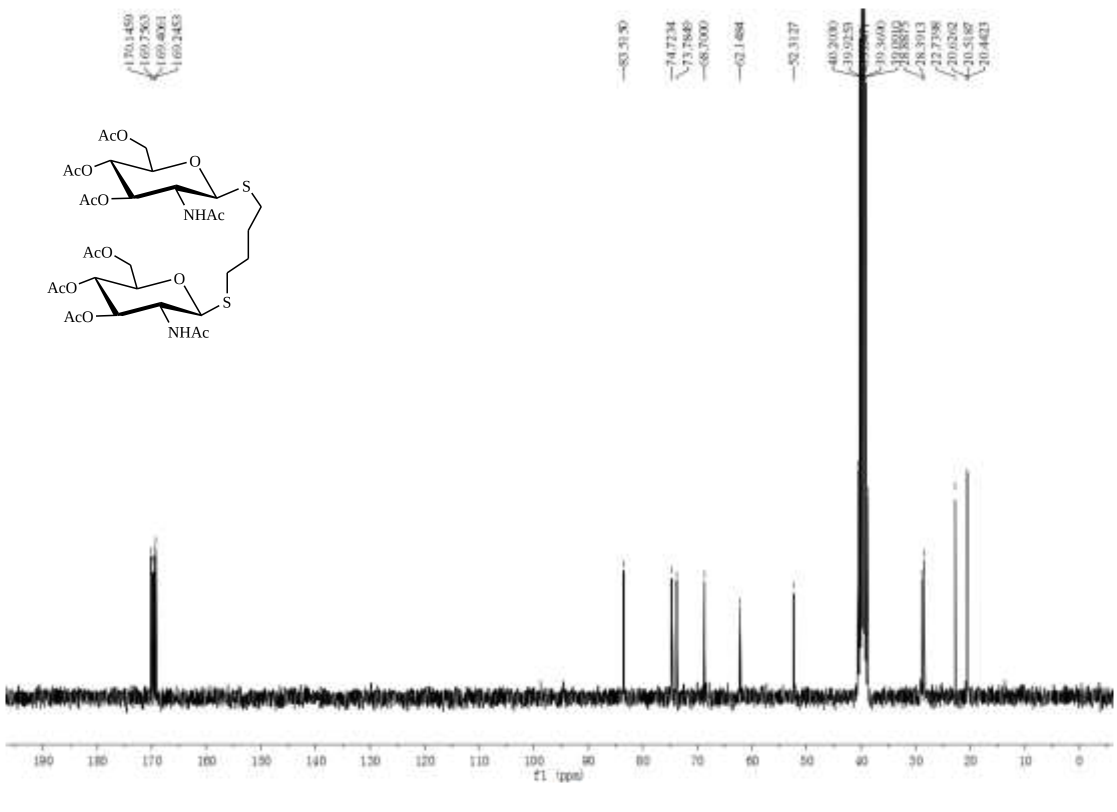

^{1}H NMR spectrum of compound 13a

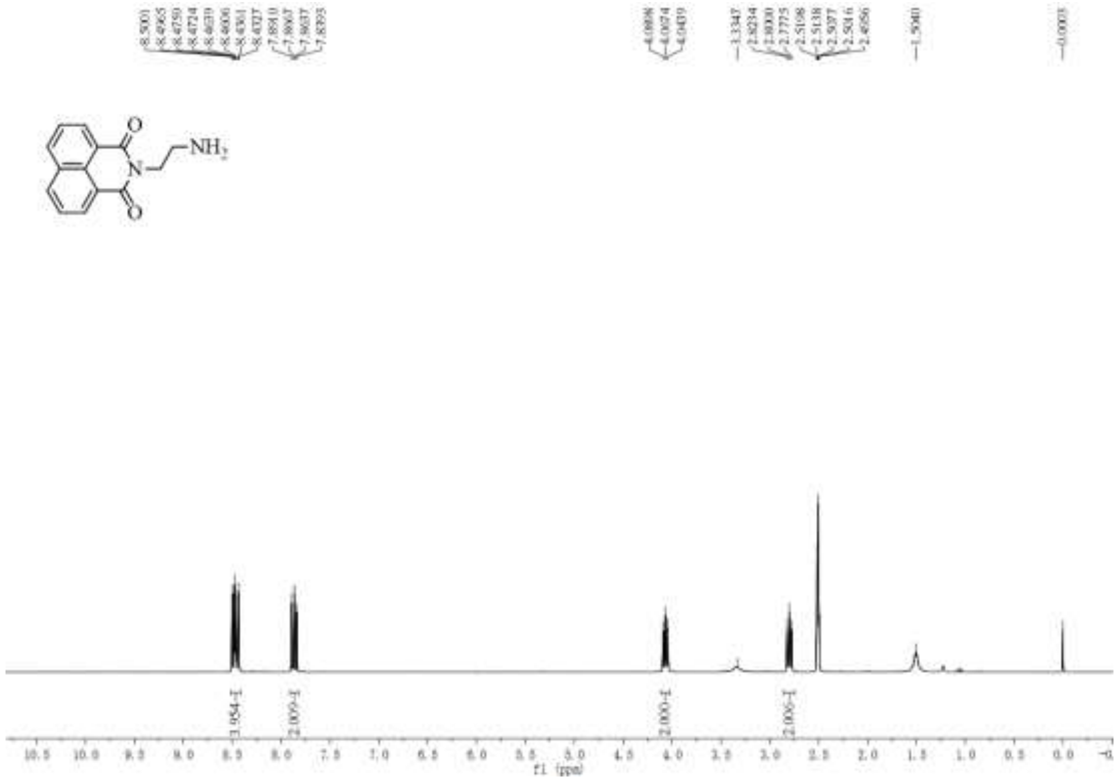

^{1}H NMR spectrum of compound 13b

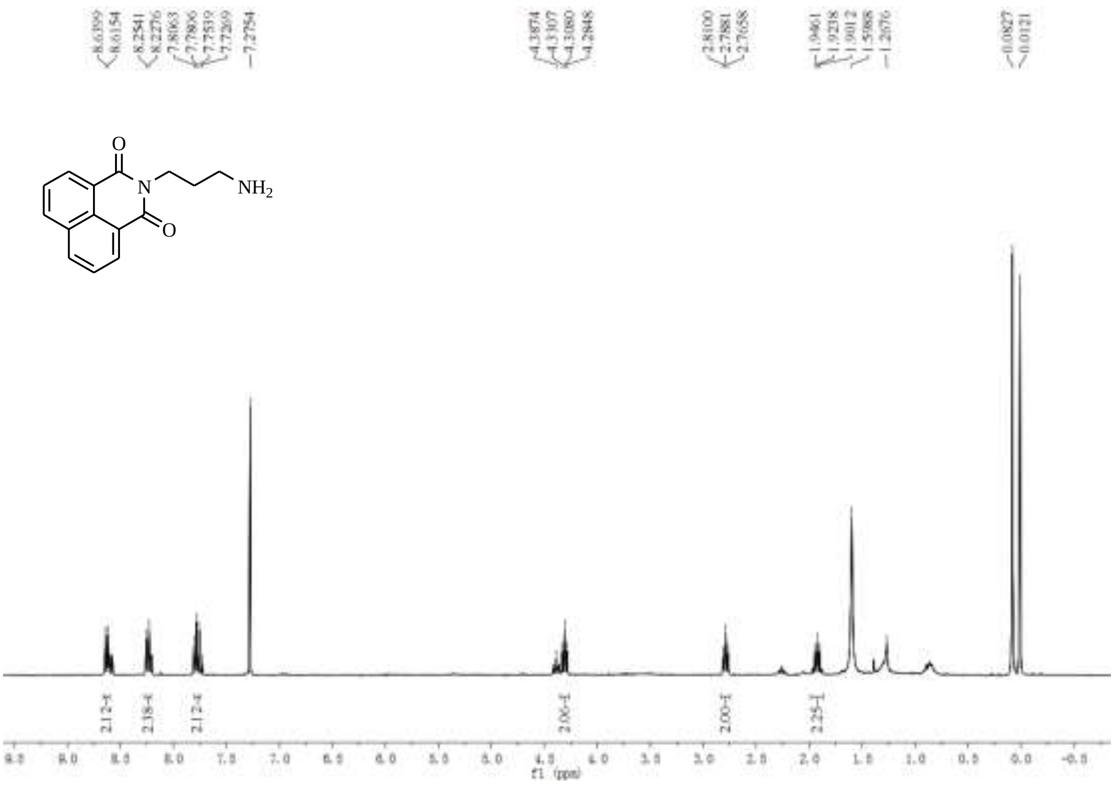

^{1}H NMR spectrum of compound 13c

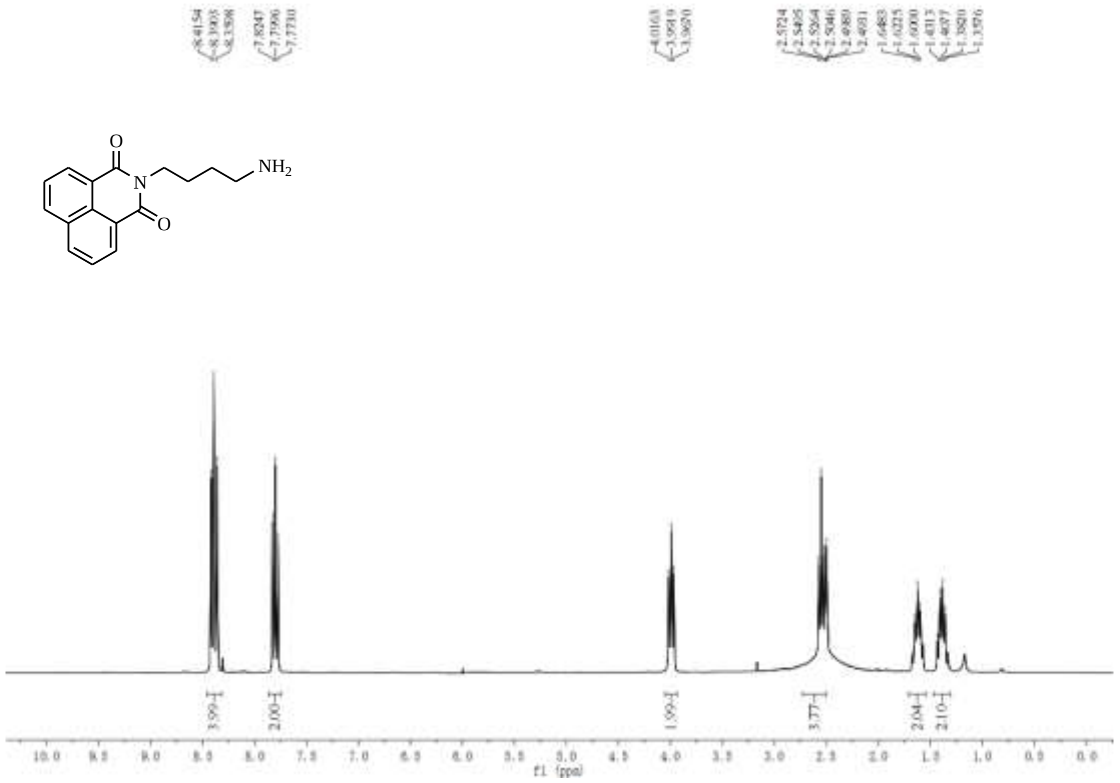

^{13}C NMR spectrum of compound 13c

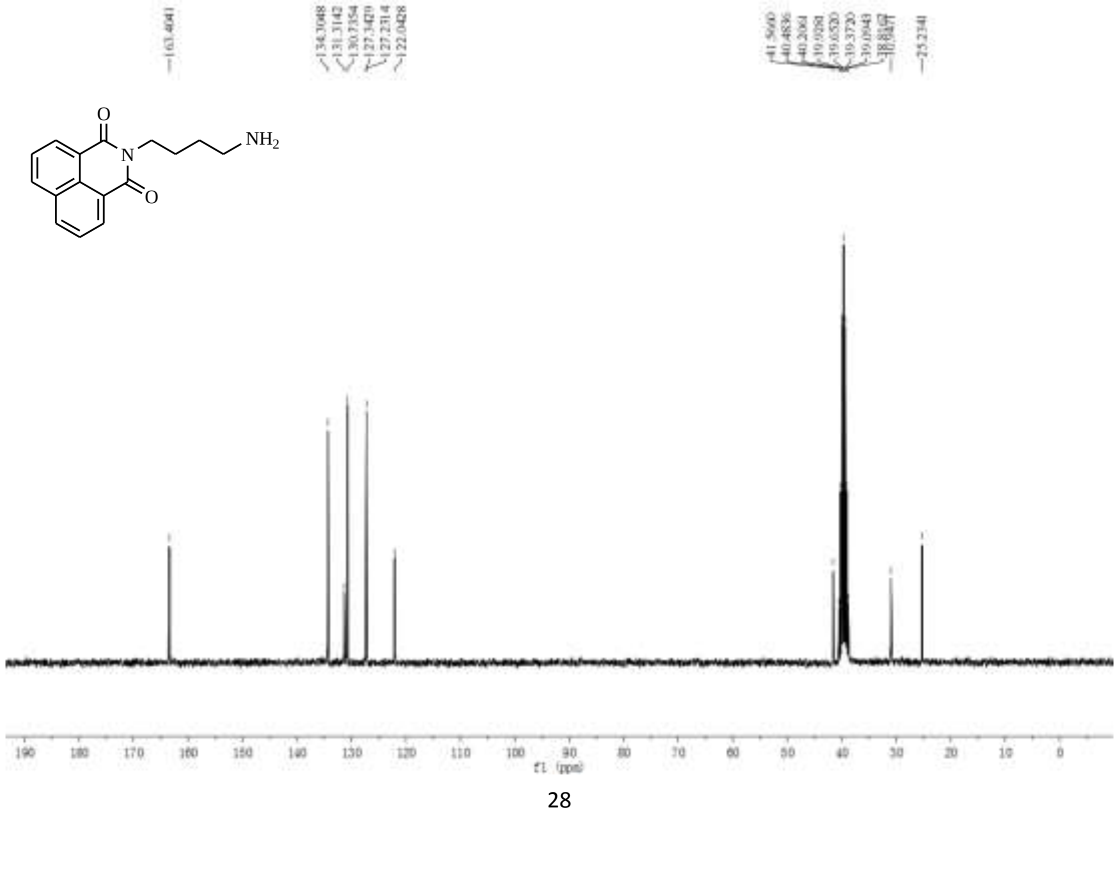

^{1}H NMR spectrum of compound 14a

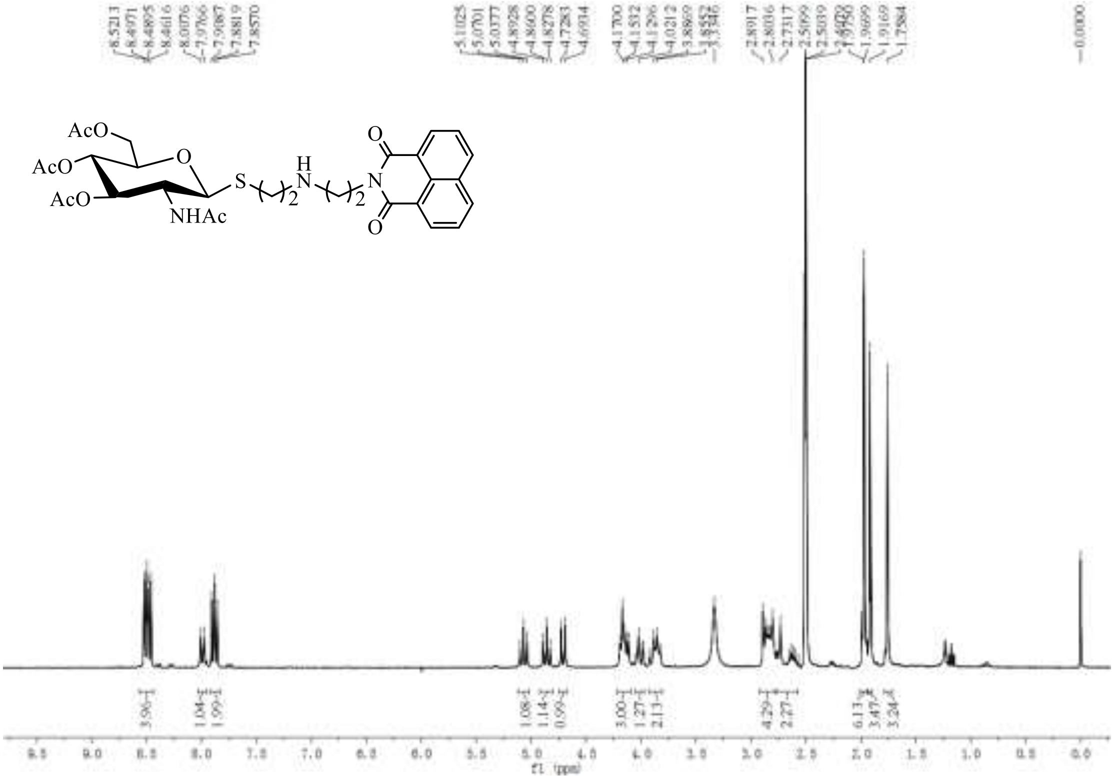

^{13}C NMR spectrum of compound 14a

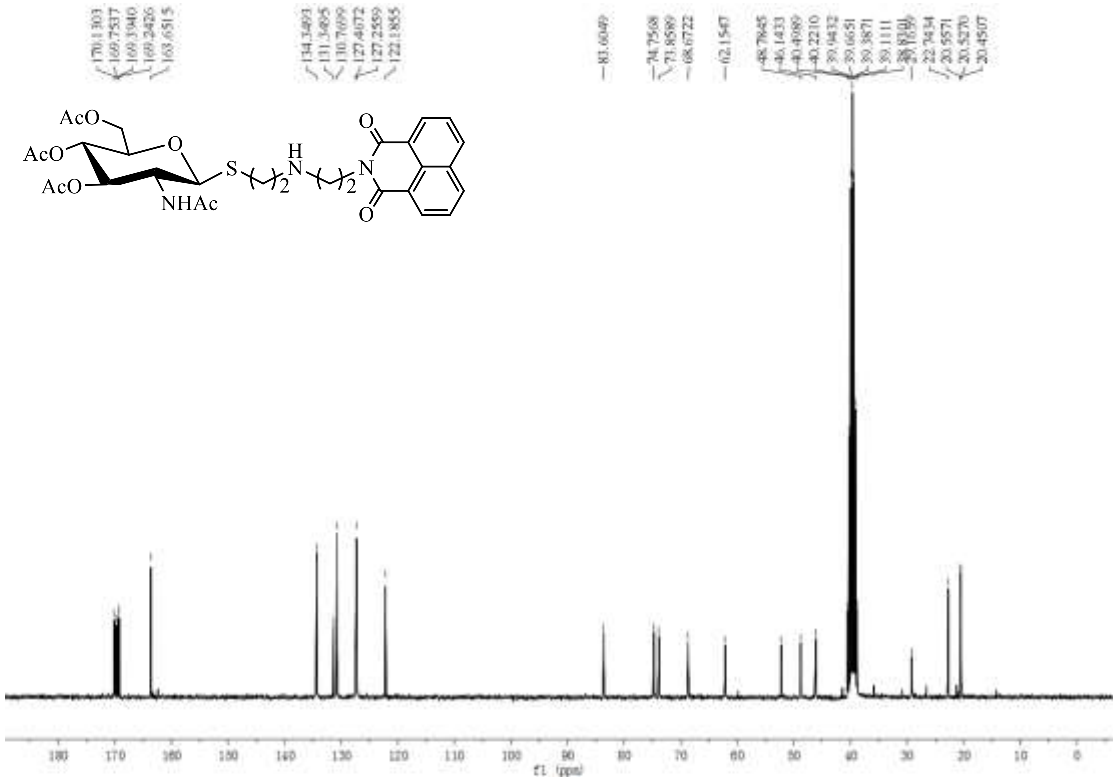

^{1}H NMR spectrum of compound 14b

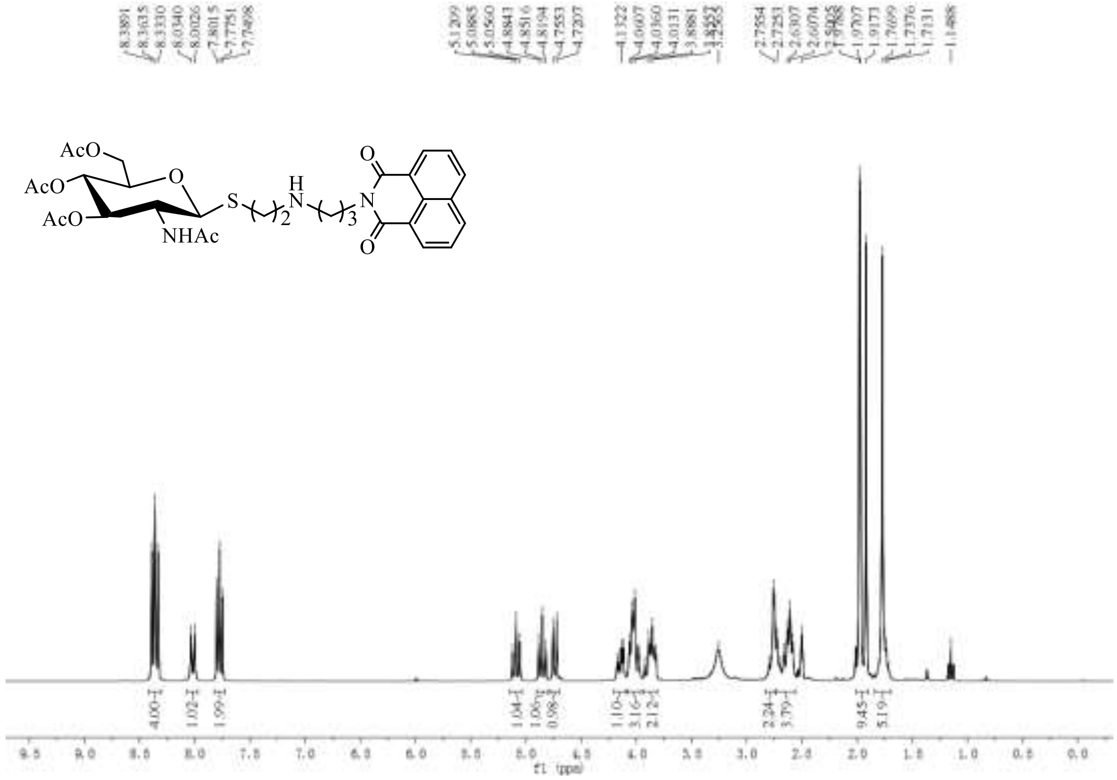

^{13}C NMR spectrum of compound 14b

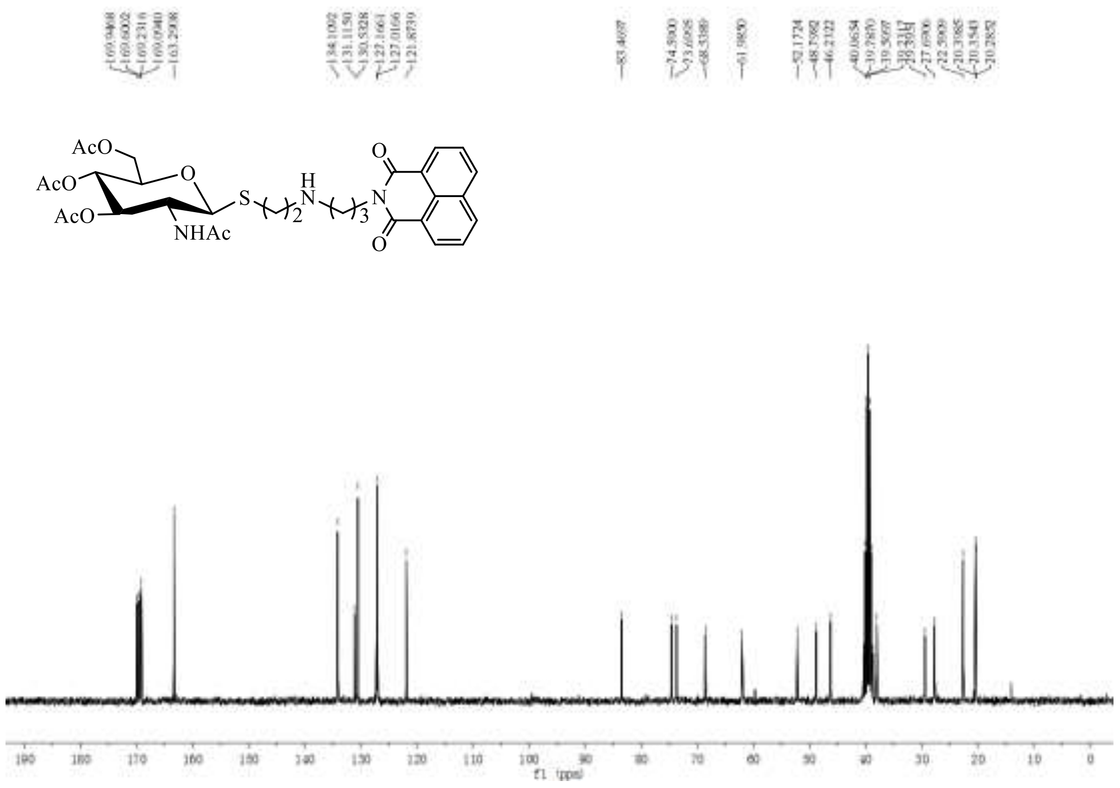

^{1}H NMR spectrum of compound 14c

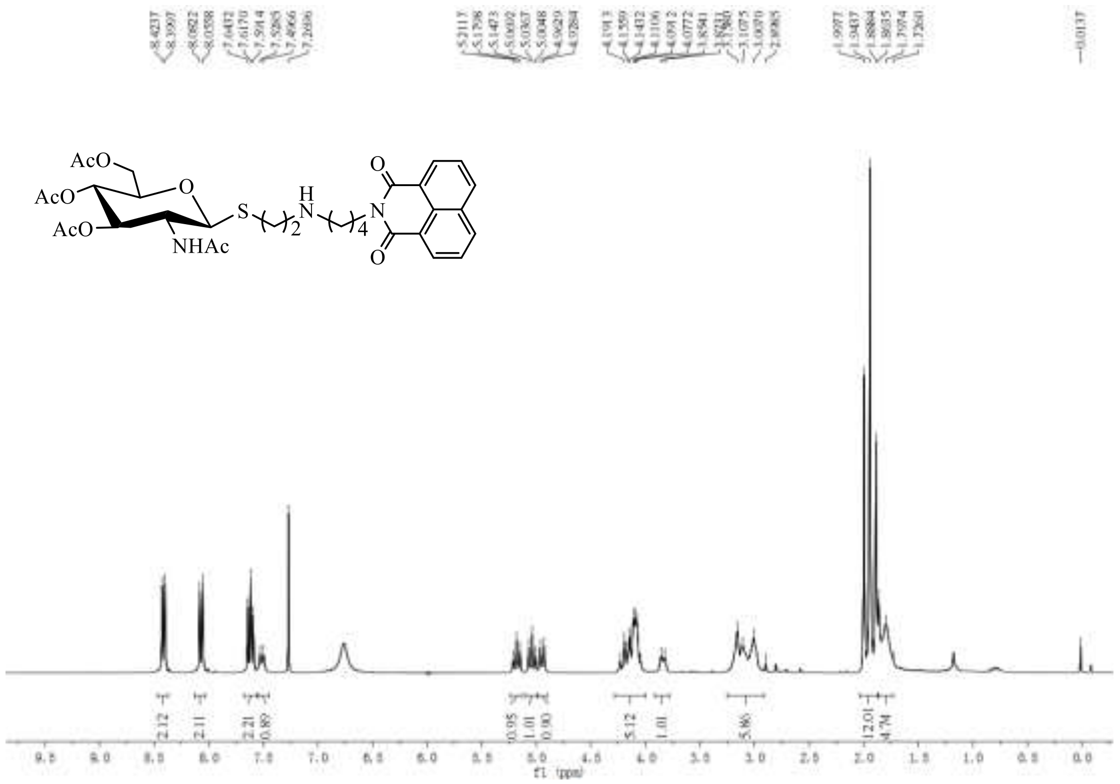

^{13}C NMR spectrum of compound 14c

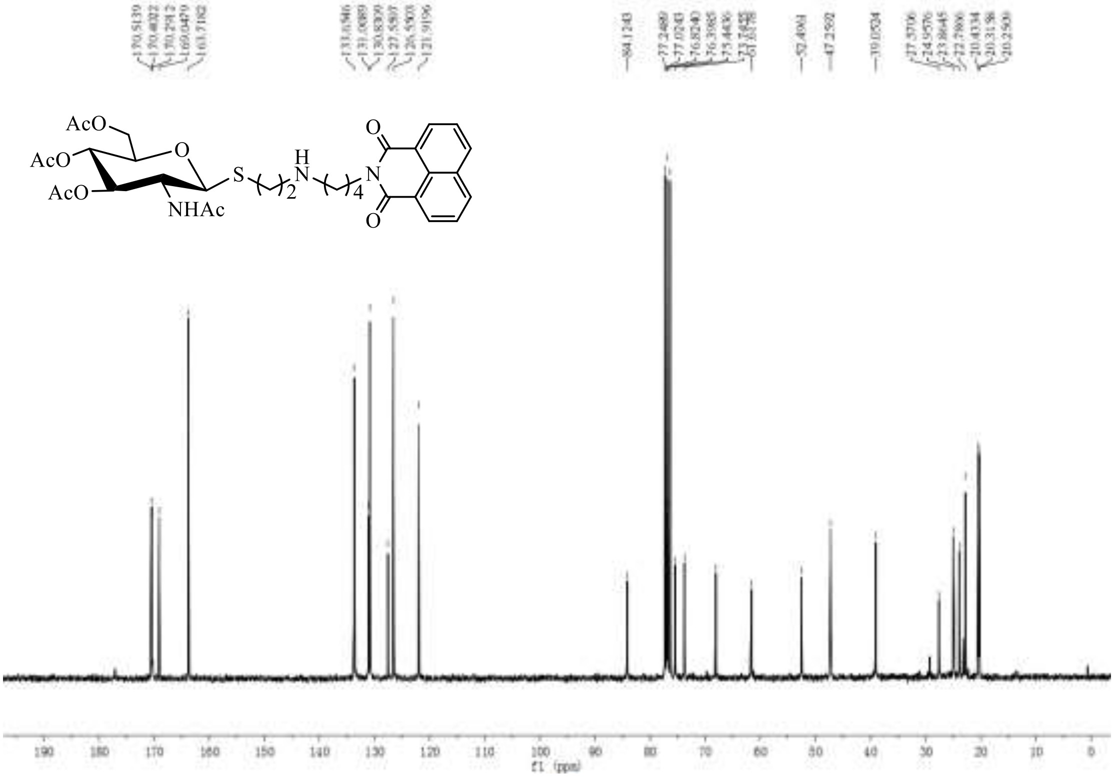

^{1}H NMR spectrum of compound 14d

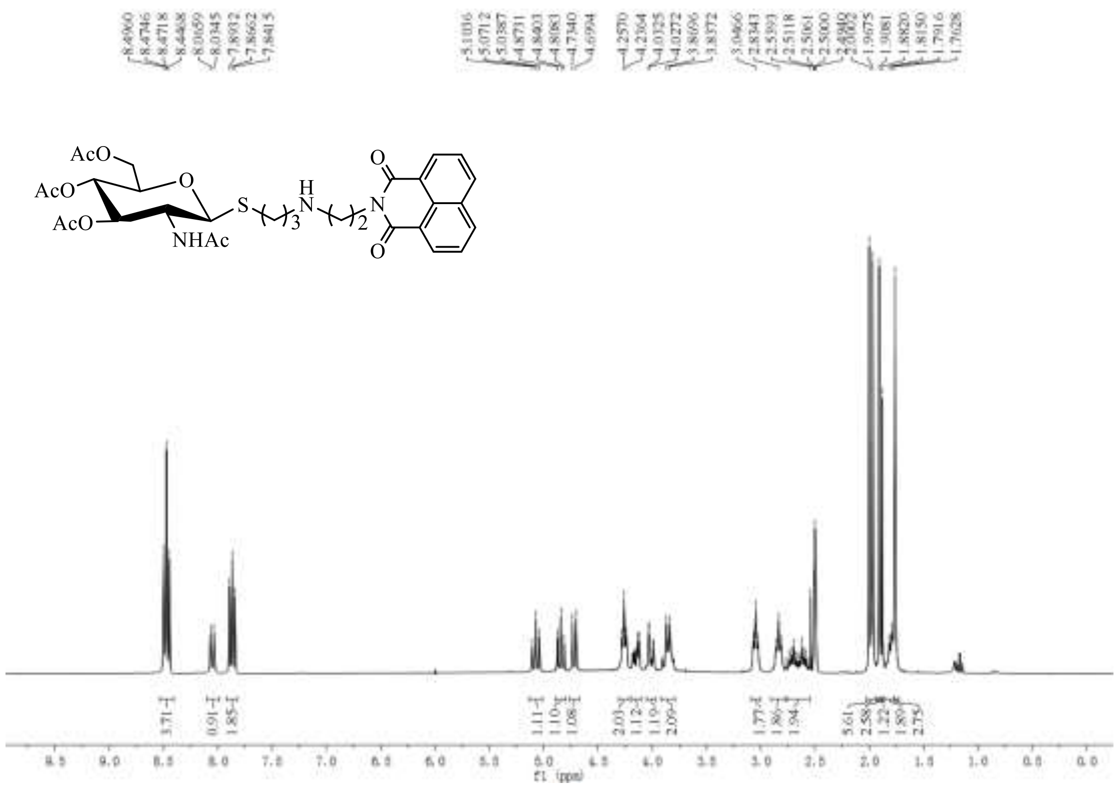

^{13}C NMR spectrum of compound 14d

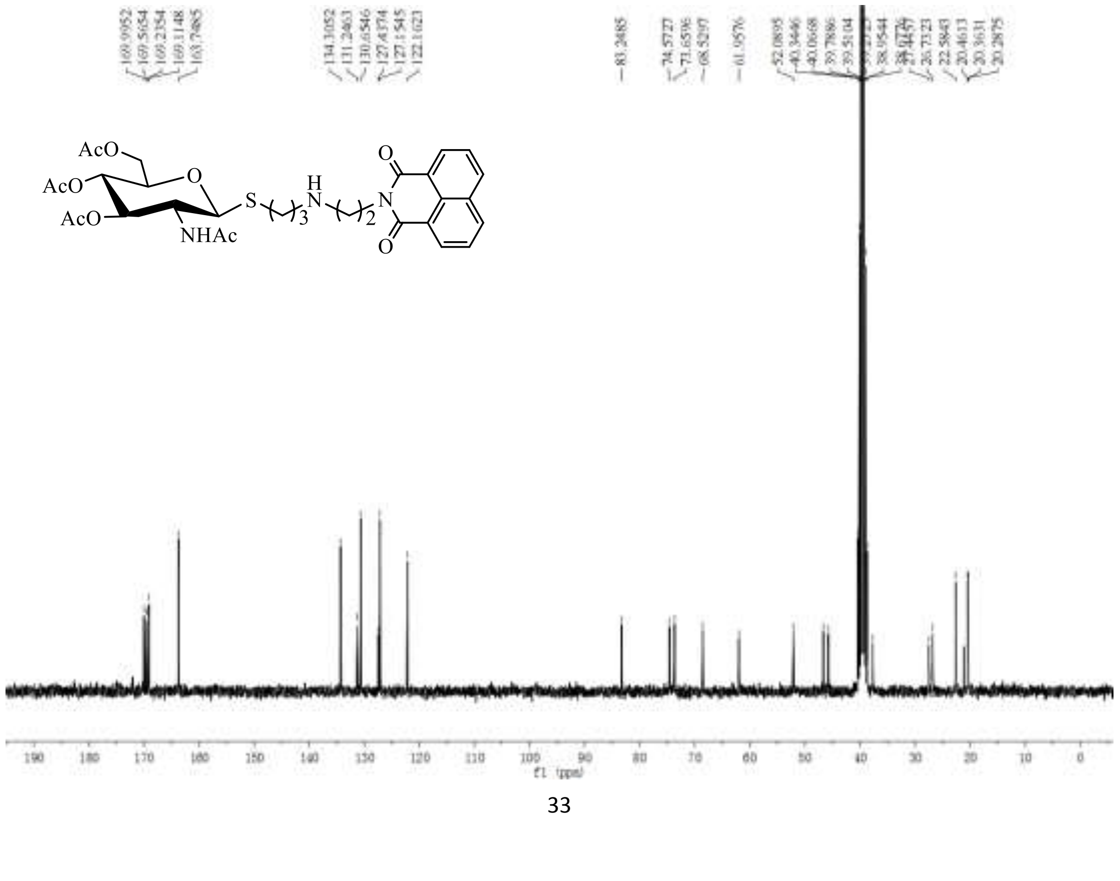

^{1}H NMR spectrum of compound 14e

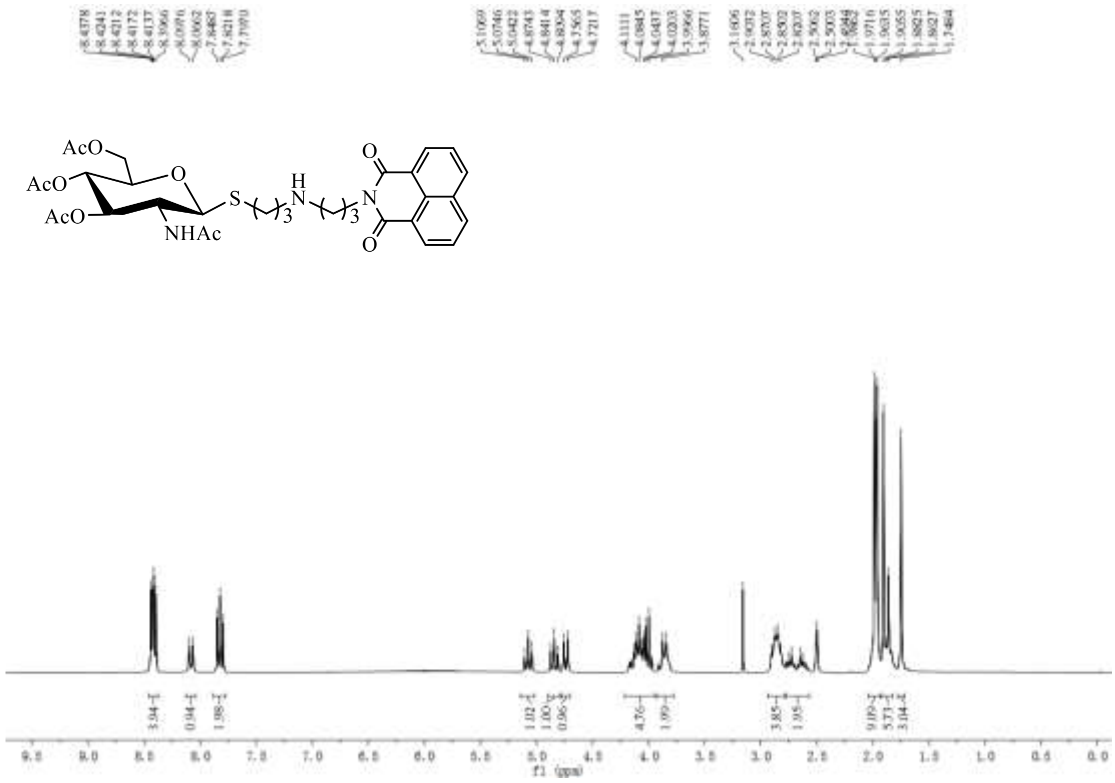

^{13}C NMR spectrum of compound 14e

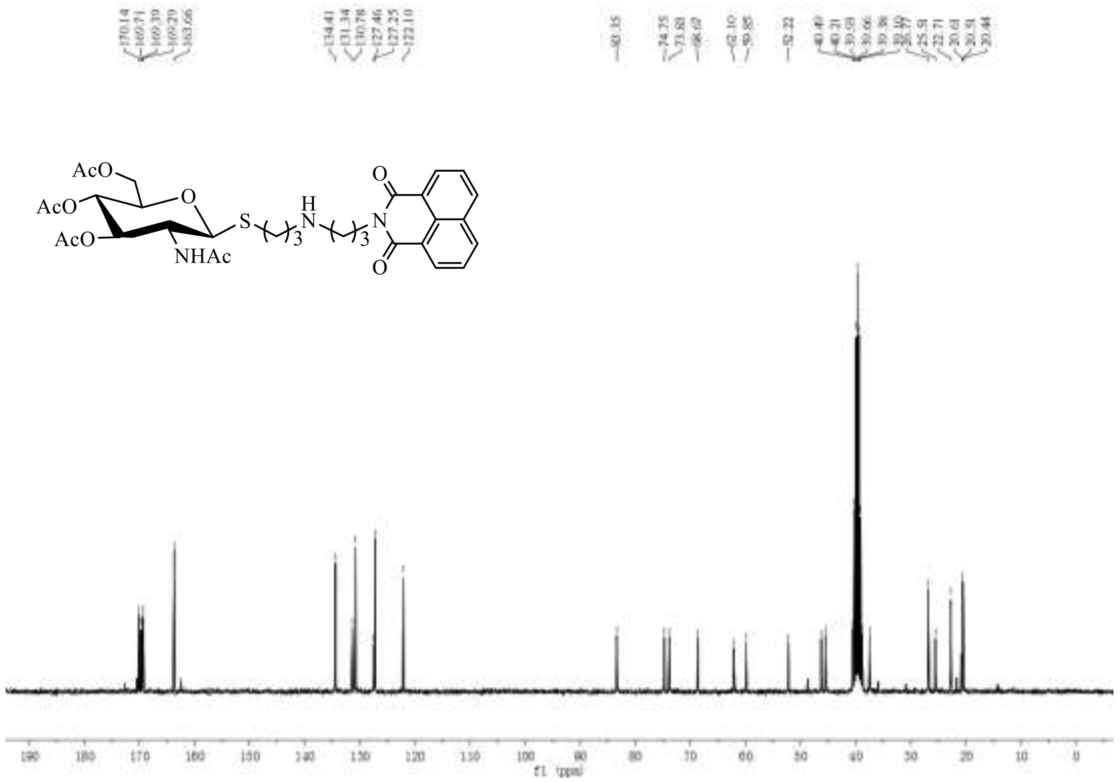

^{1}H NMR spectrum of compound 14f

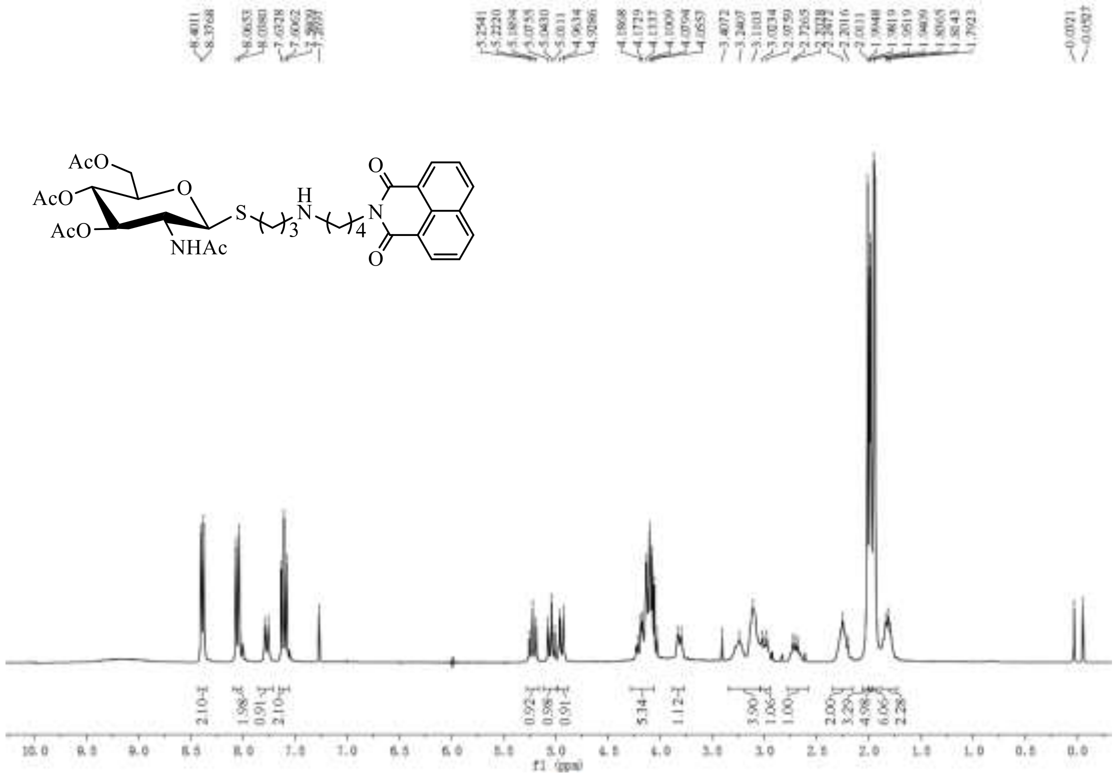

^{13}C NMR spectrum of compound 14f

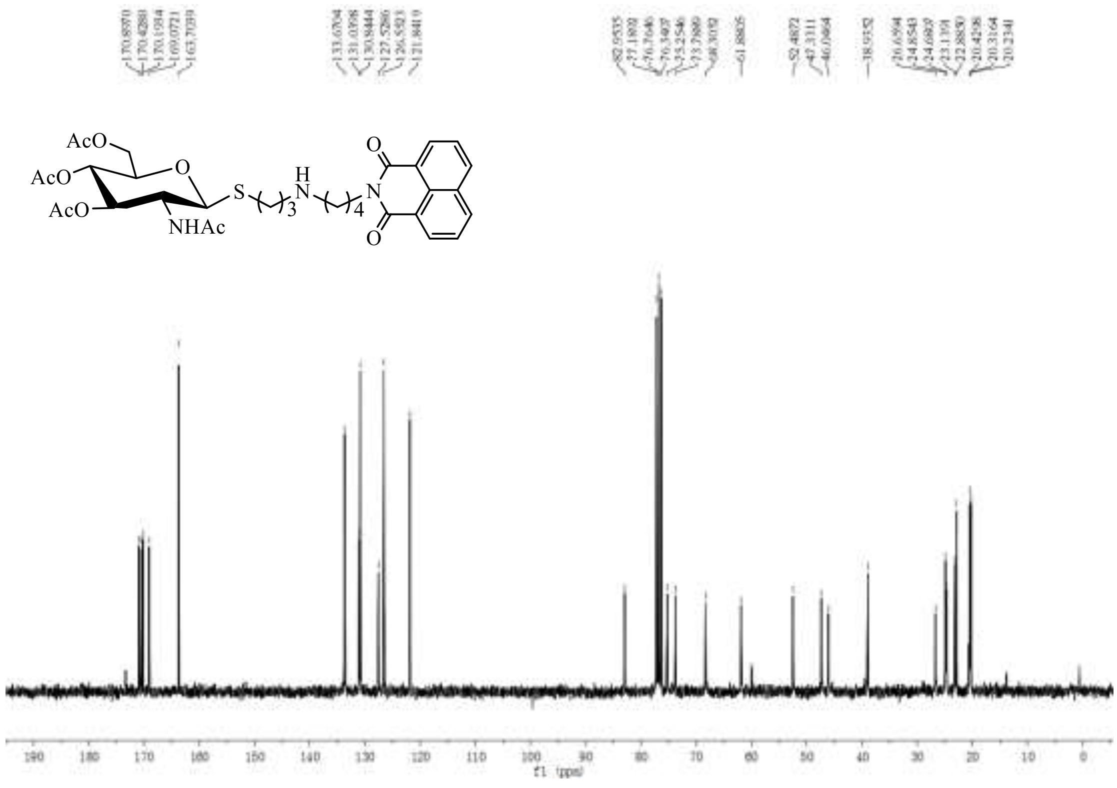

^{1}H NMR spectrum of compound 14g

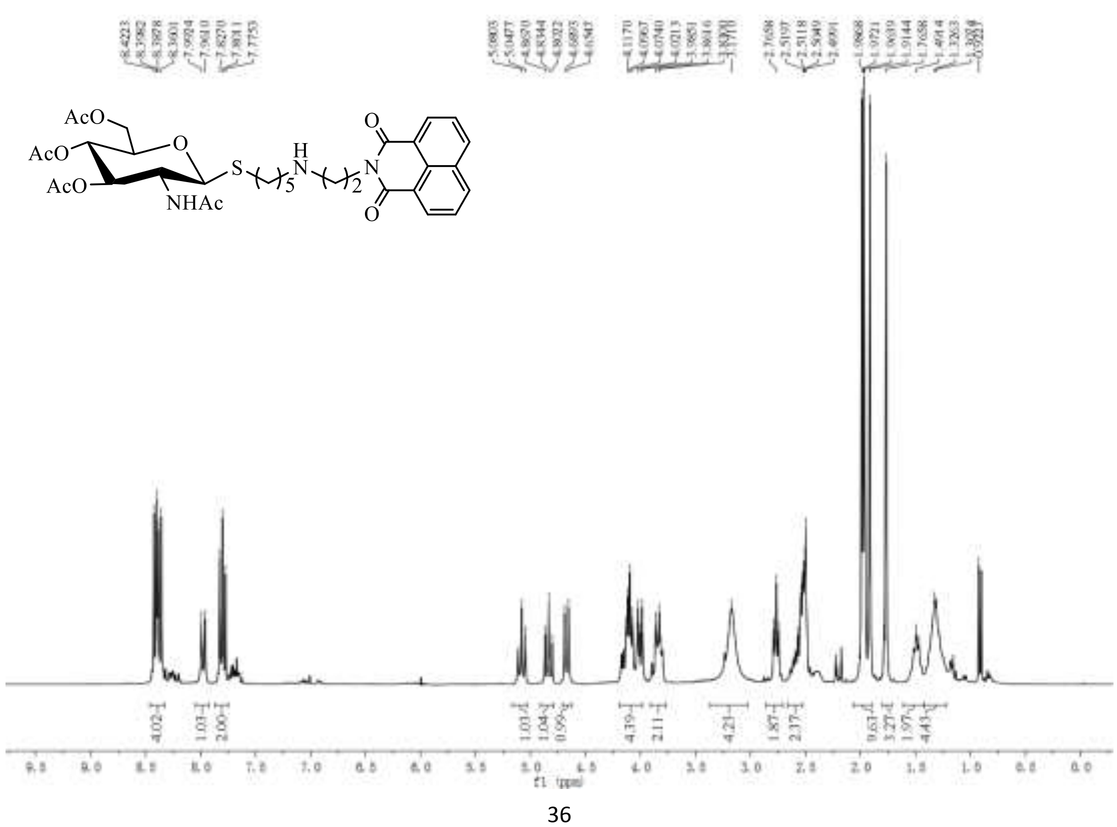

^{13}C NMR spectrum of compound 14g

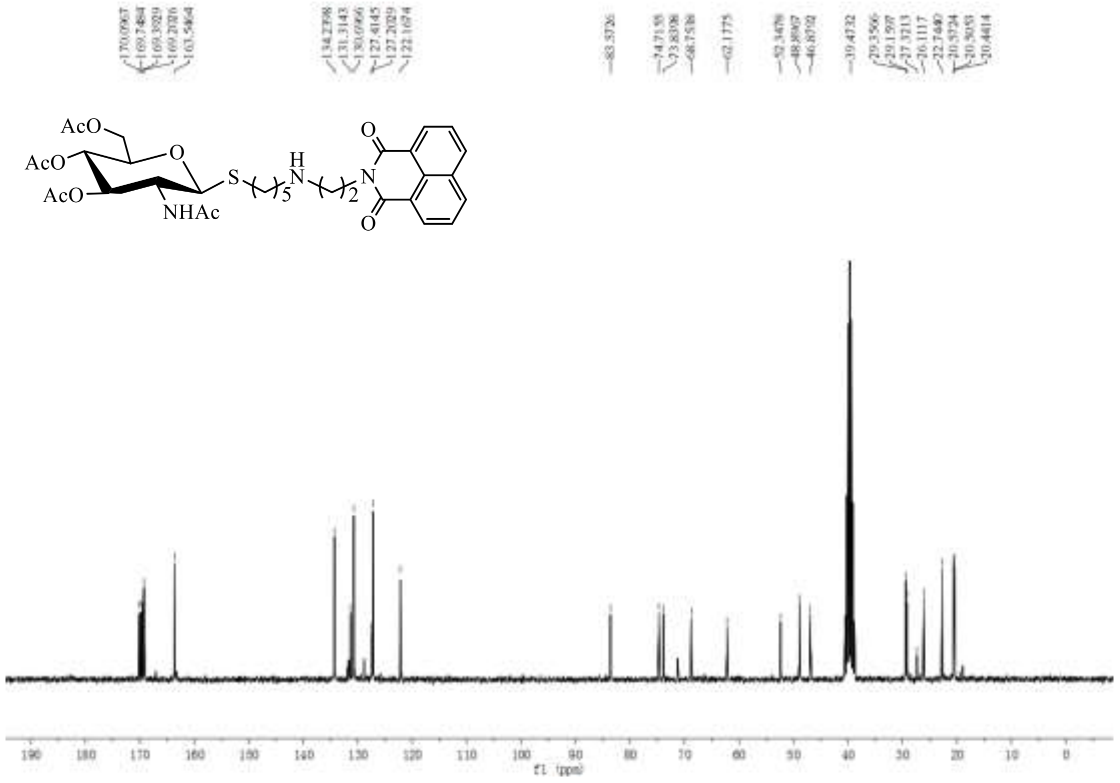

^{1}H NMR spectrum of compound 14h

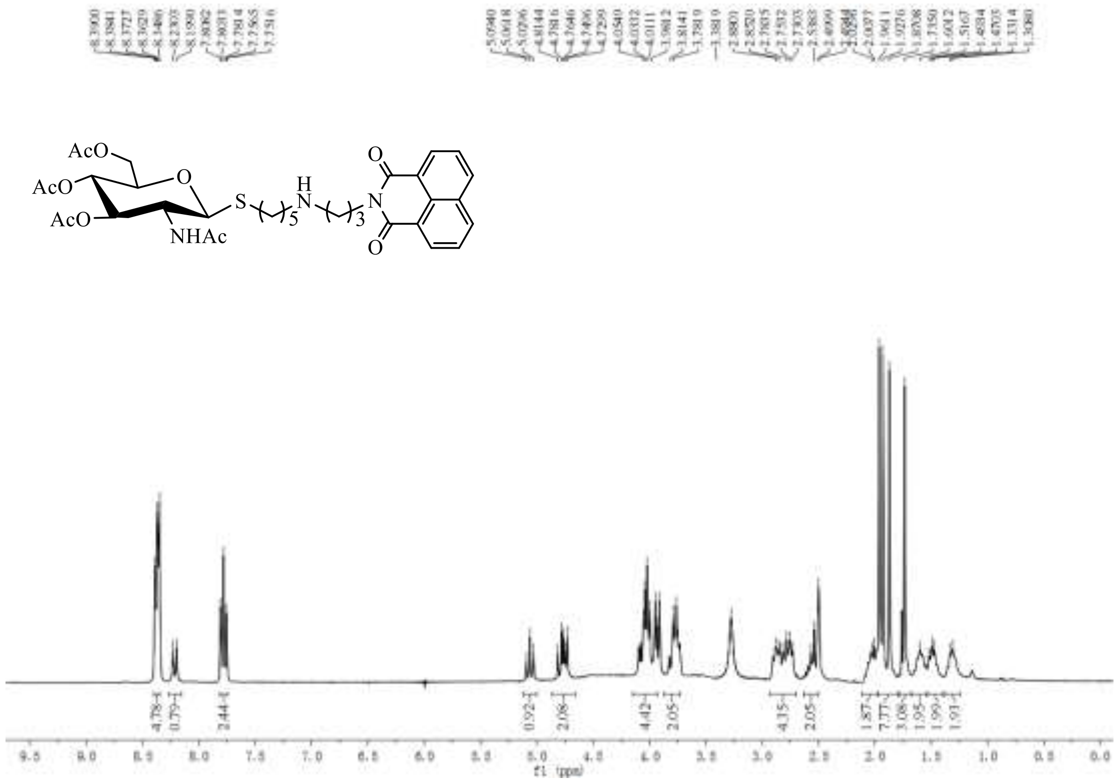

^{13}C NMR spectrum of compound 14h

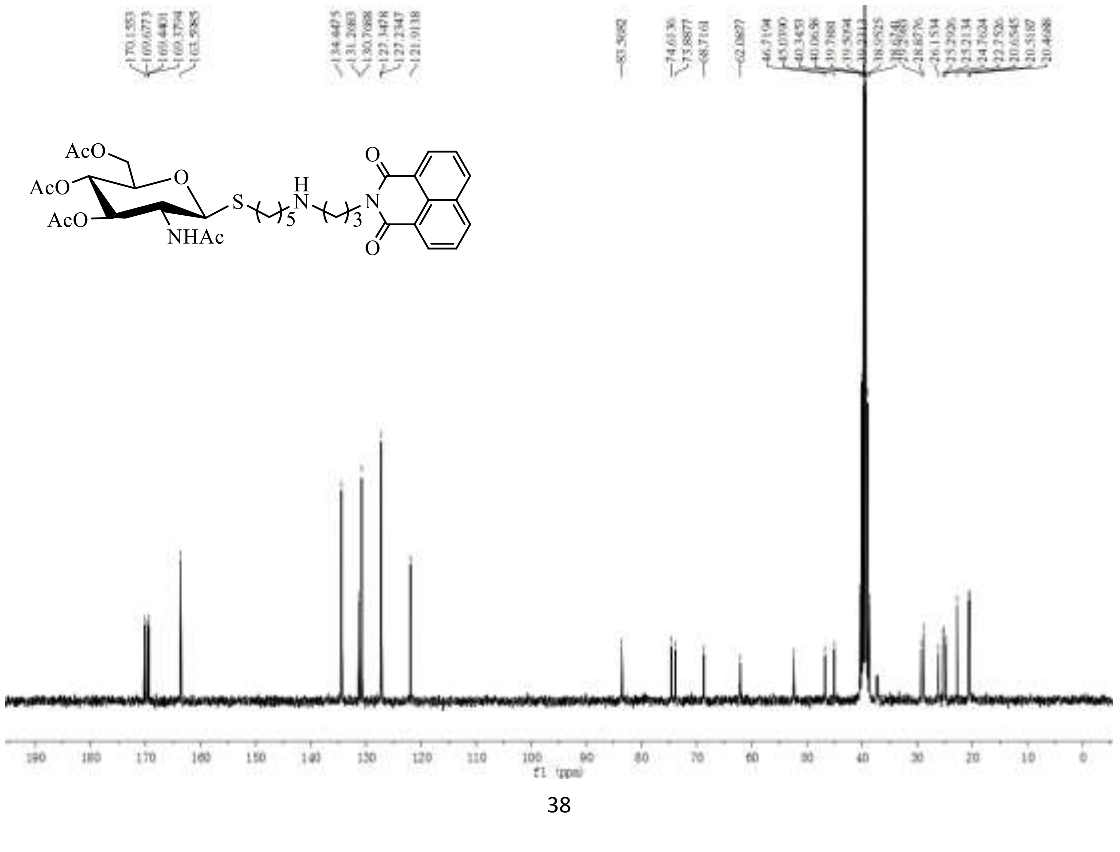

^{1}H NMR spectrum of compound 14i

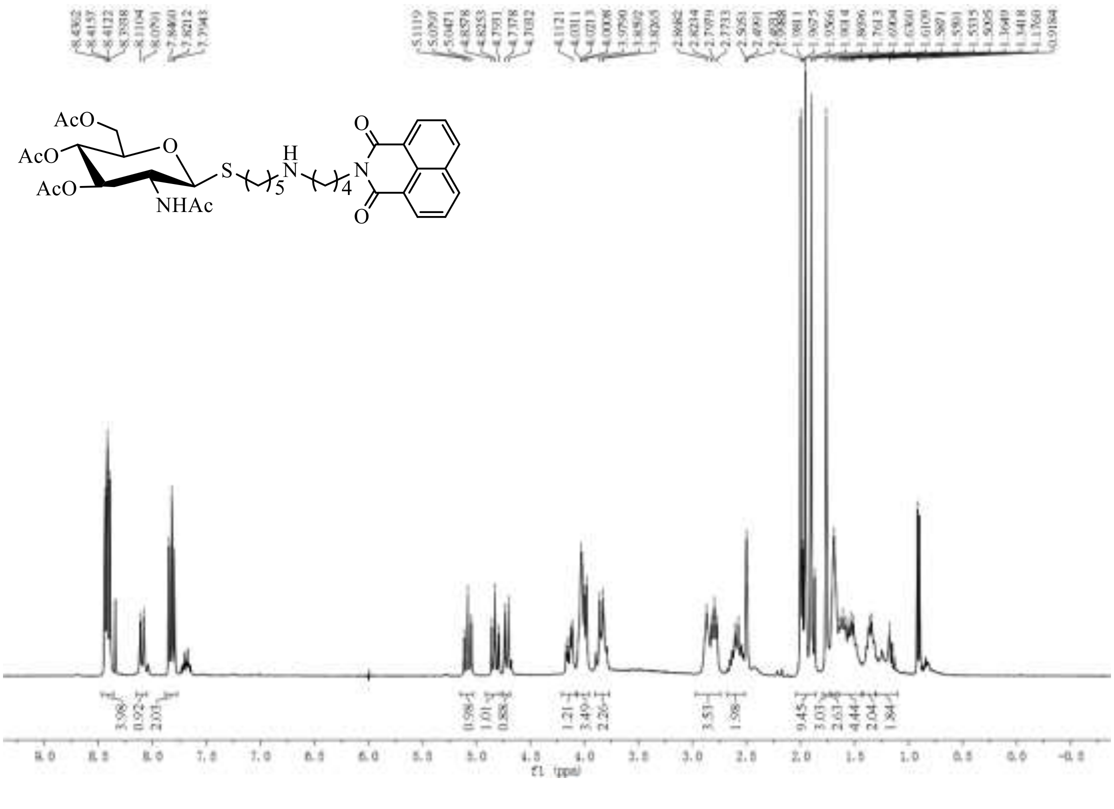

^{13}C NMR spectrum of compound 14i

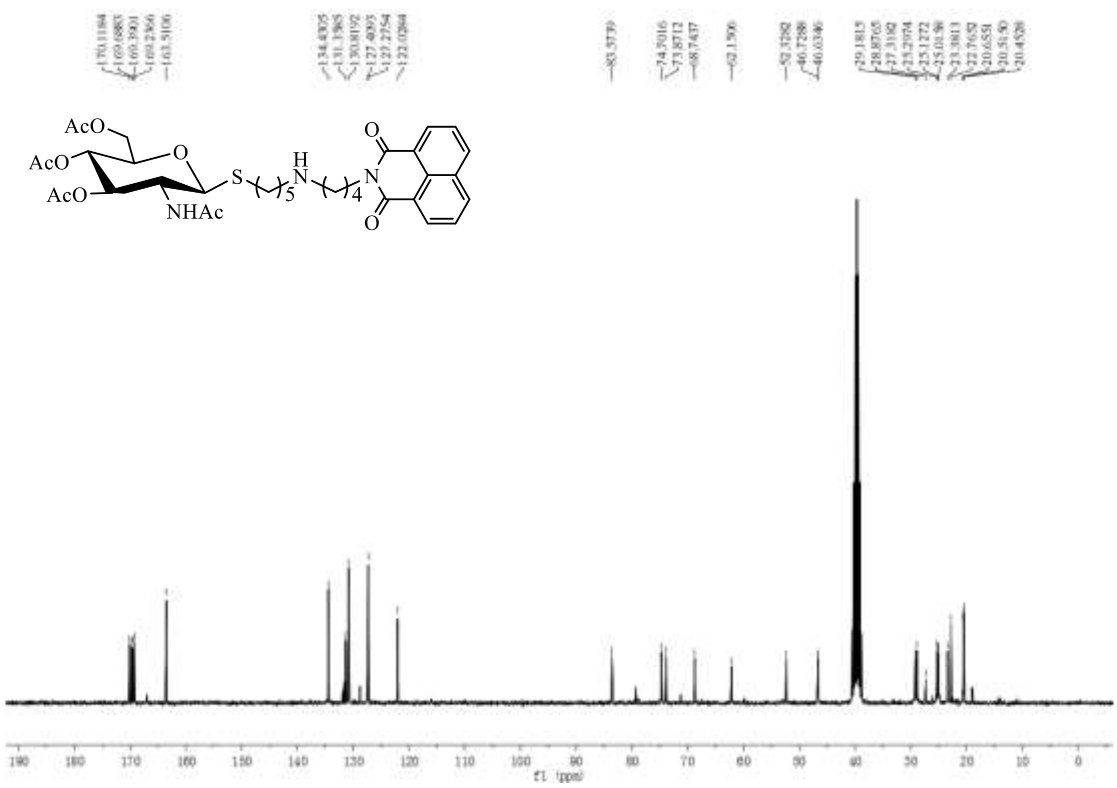

^{1}H NMR spectrum of compound 14j

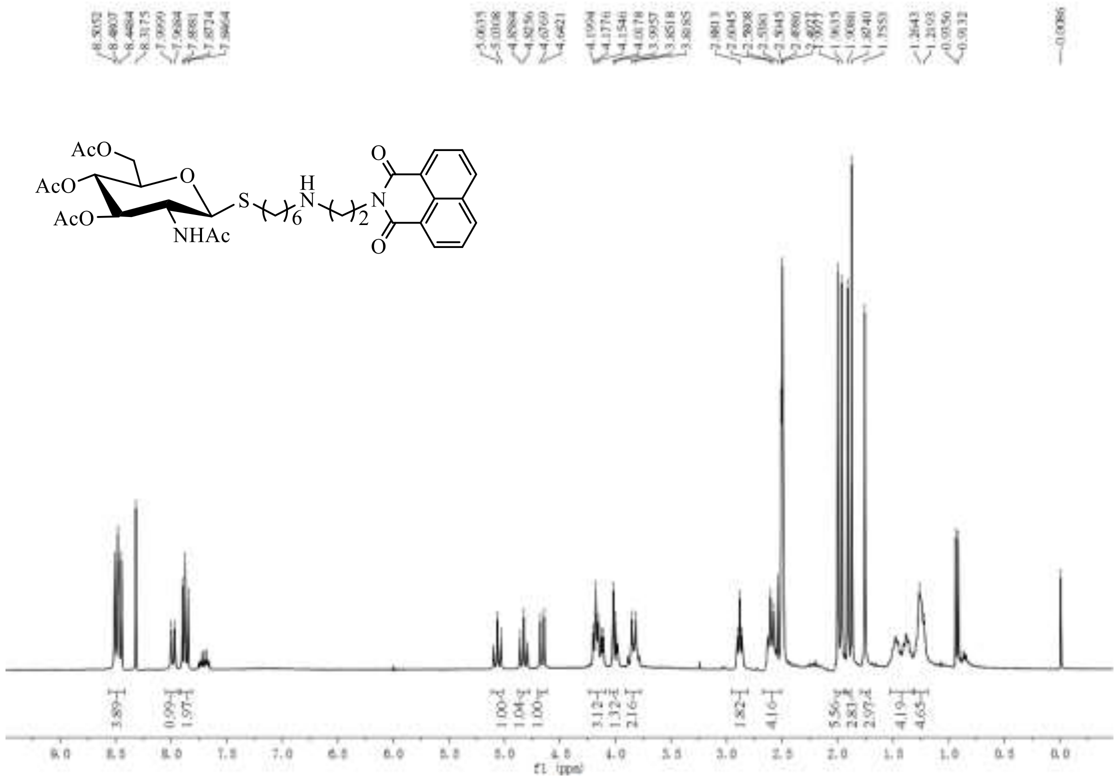

^{13}C NMR spectrum of compound 14j

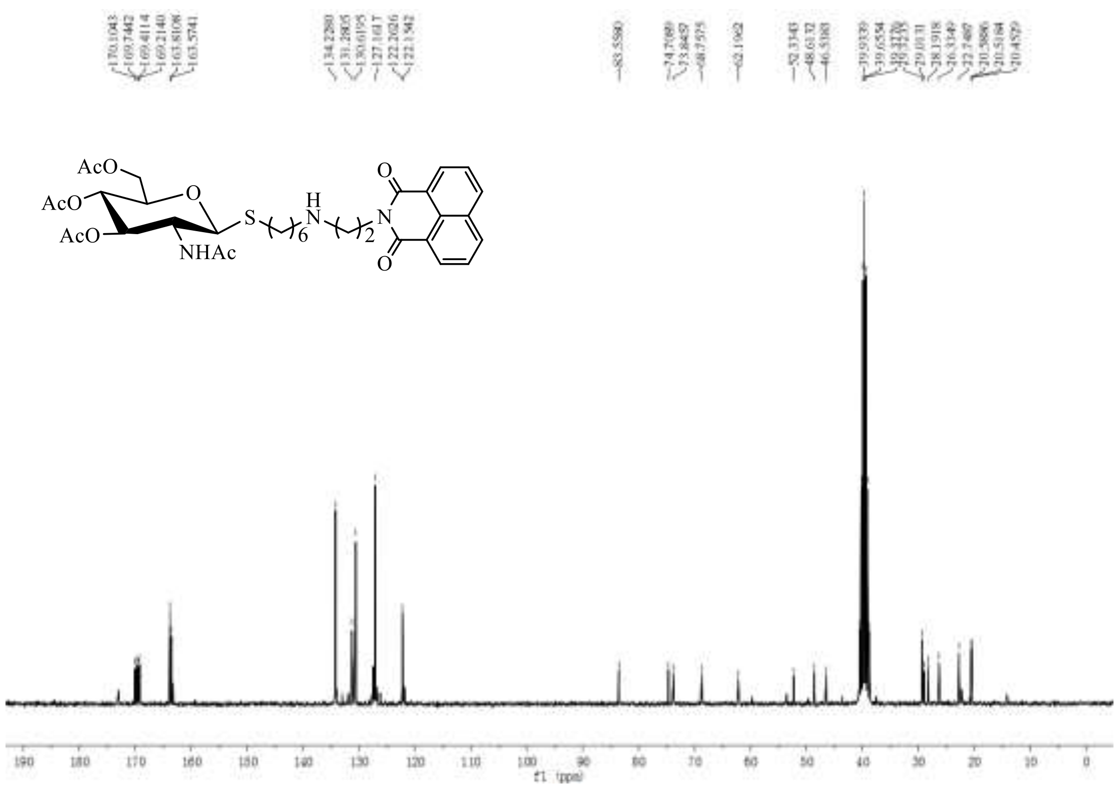

^{1}H NMR spectrum of compound 14k

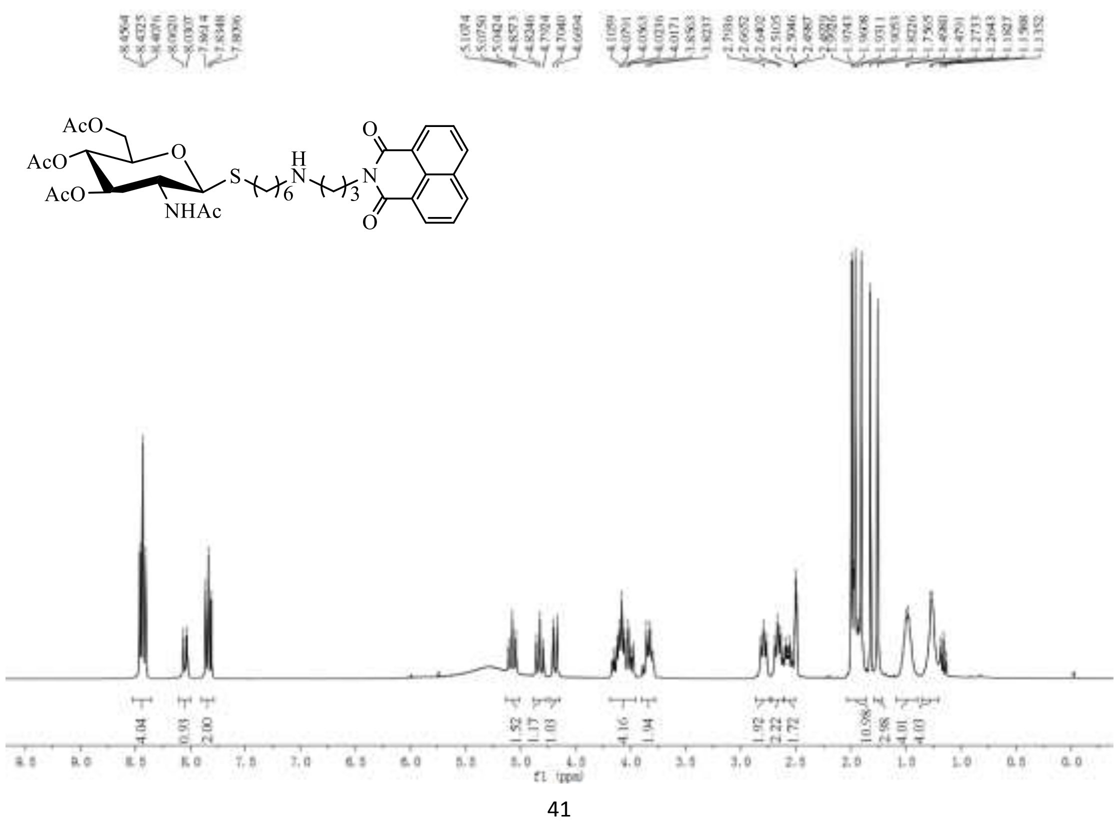

^{13}C NMR spectrum of compound 14k

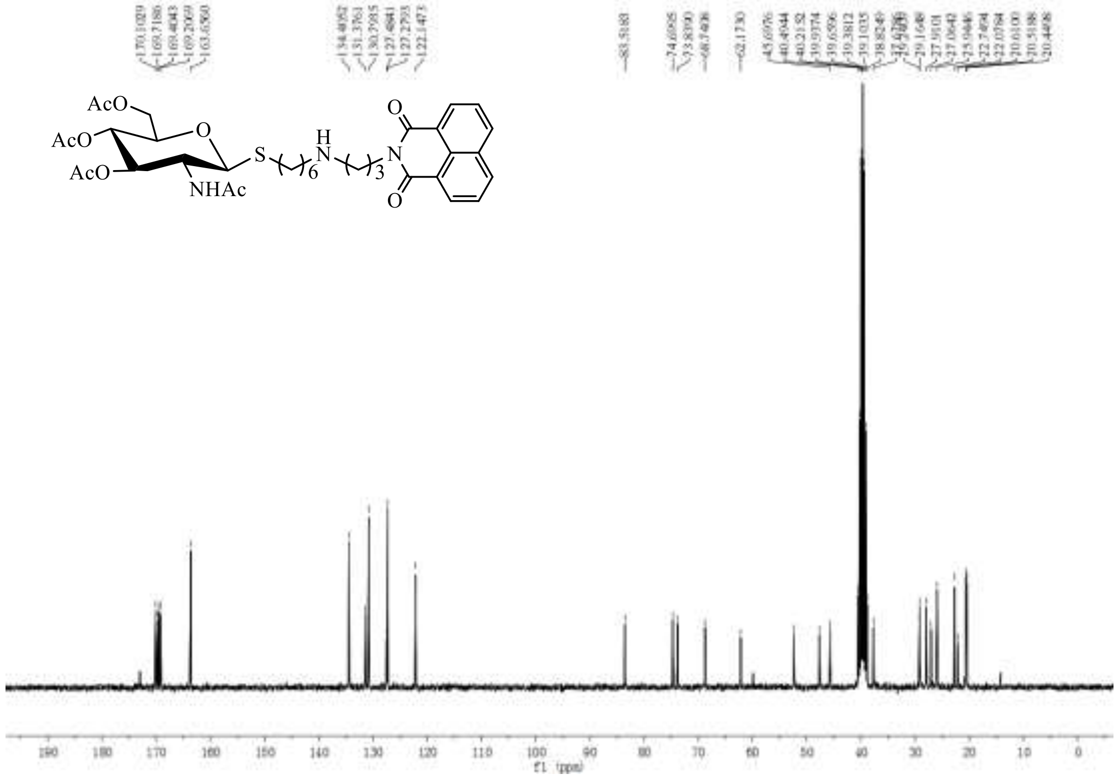

^{1}H NMR spectrum of compound 14l

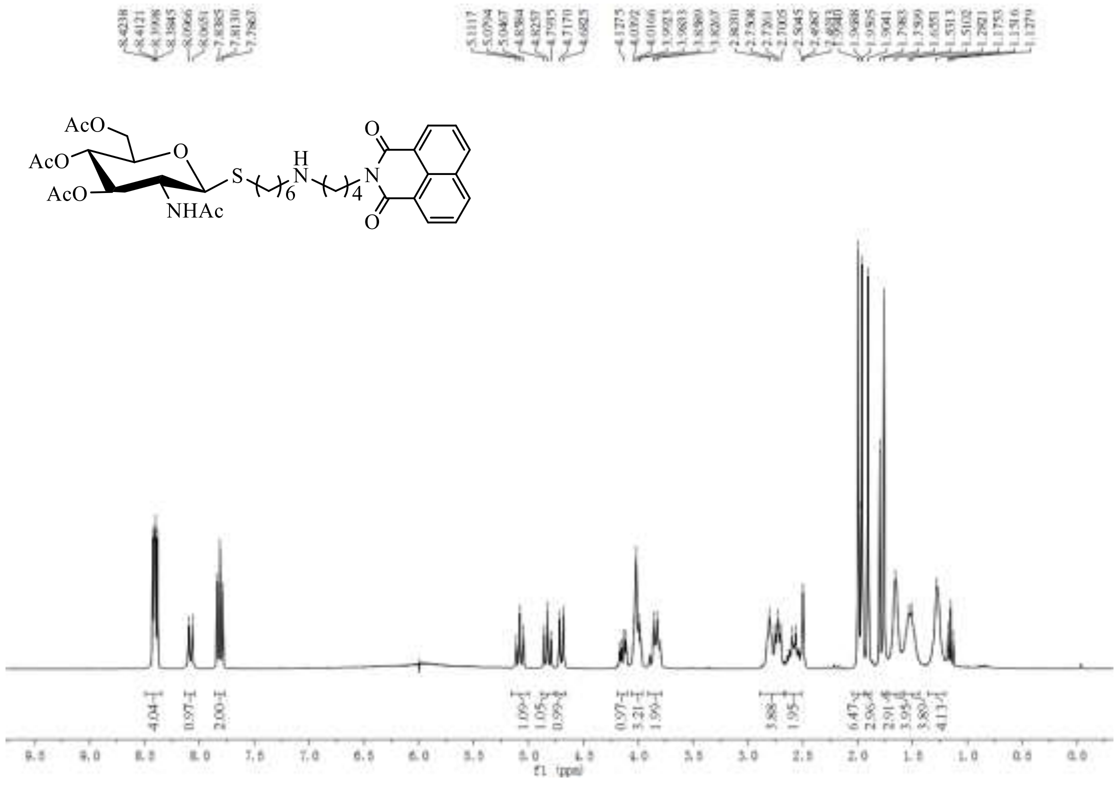

^{1}H NMR spectrum of compound 14l

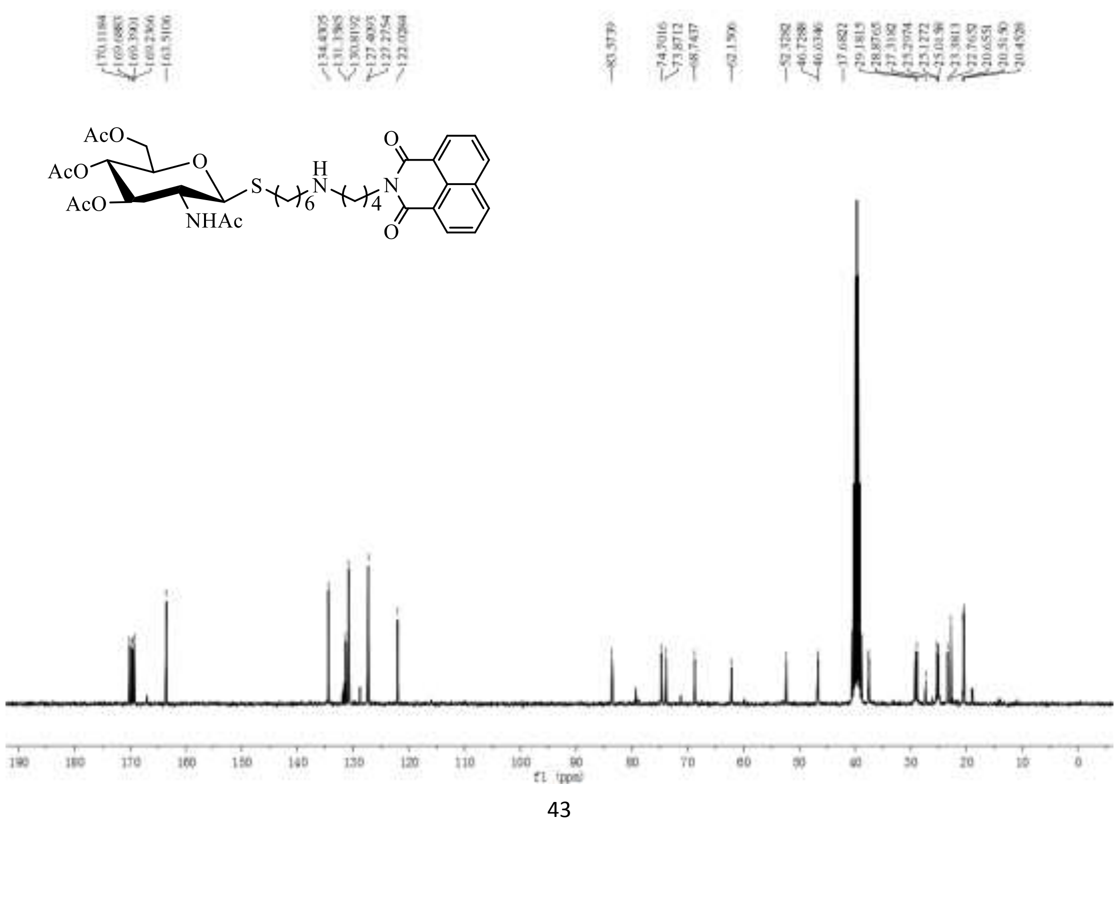

^{1}H NMR spectrum of compound 15a

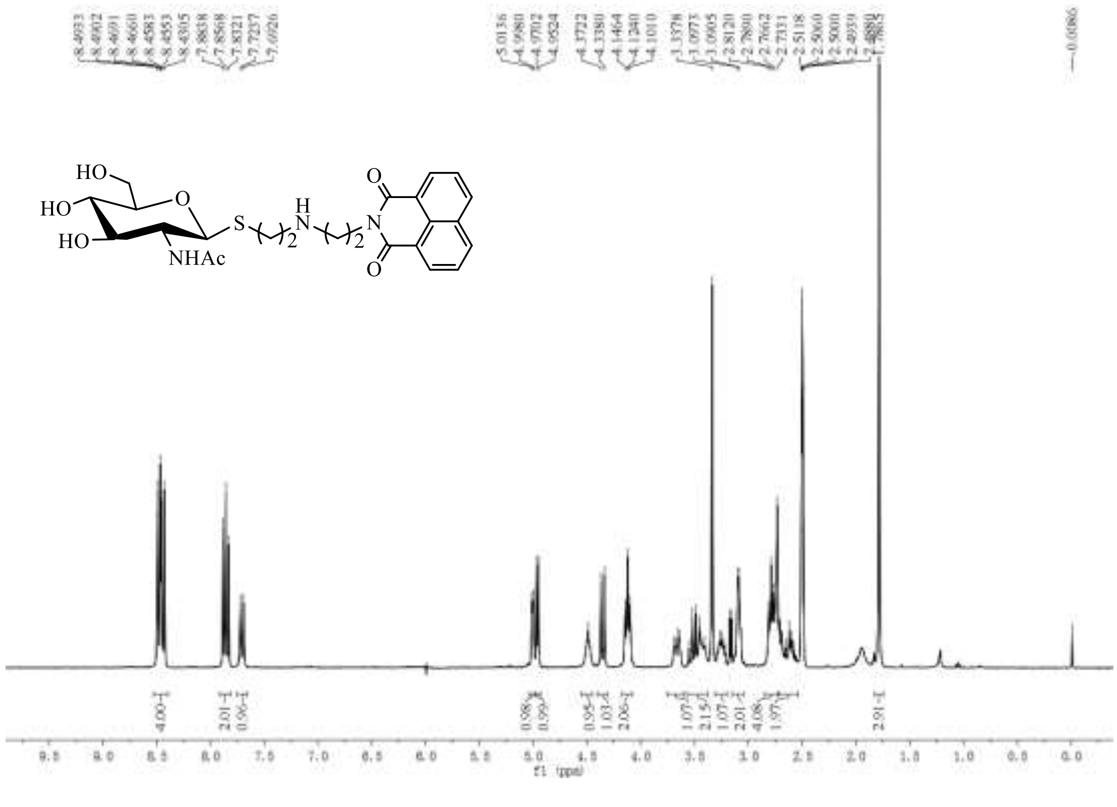

^{13}C NMR spectrum of compound 15a

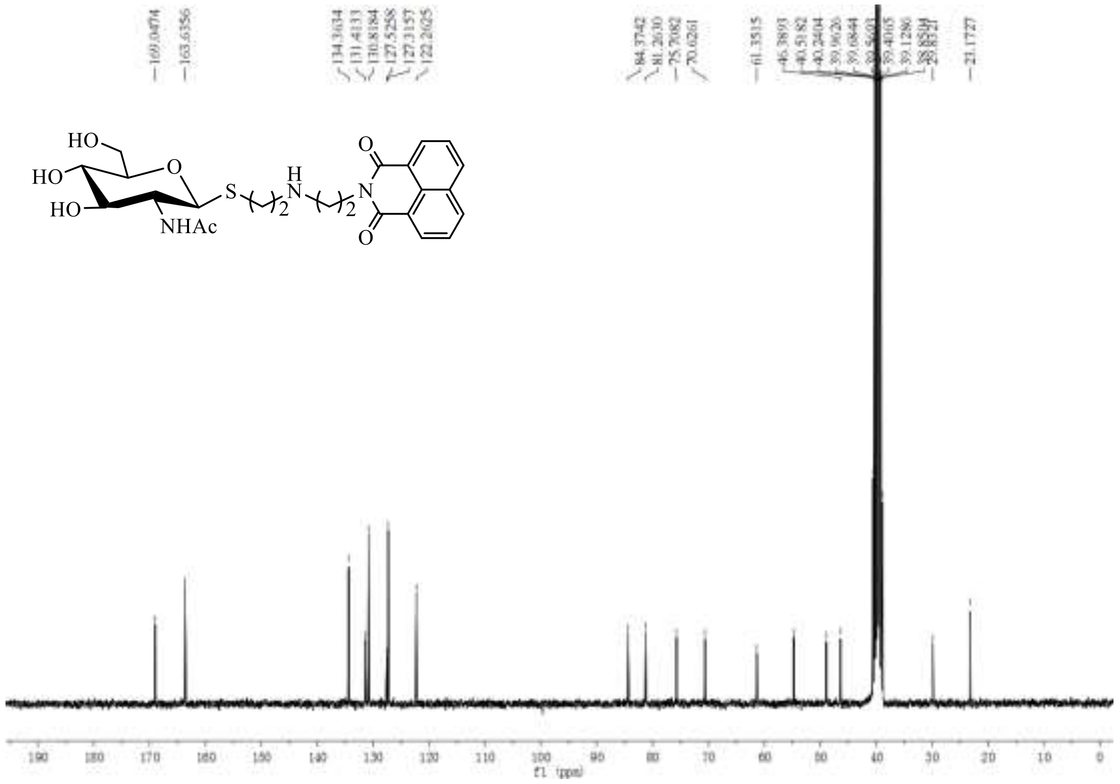

^{1}H NMR spectrum of compound 15b

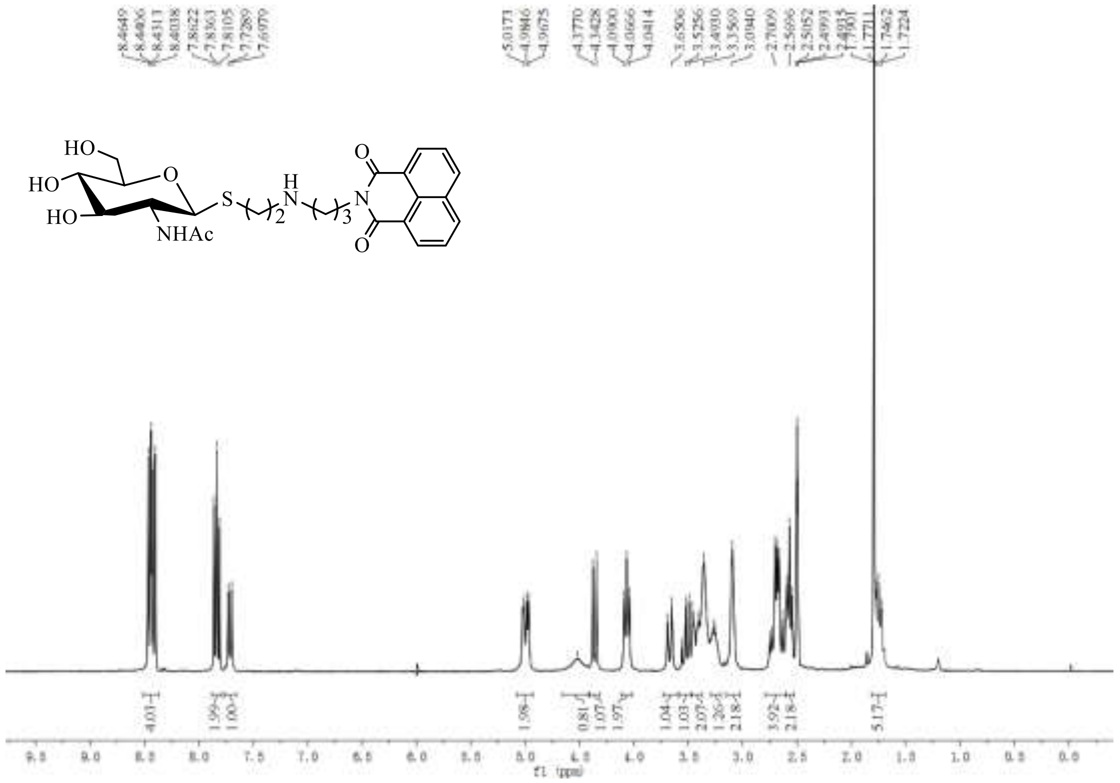

^{13}C NMR spectrum of compound 15b

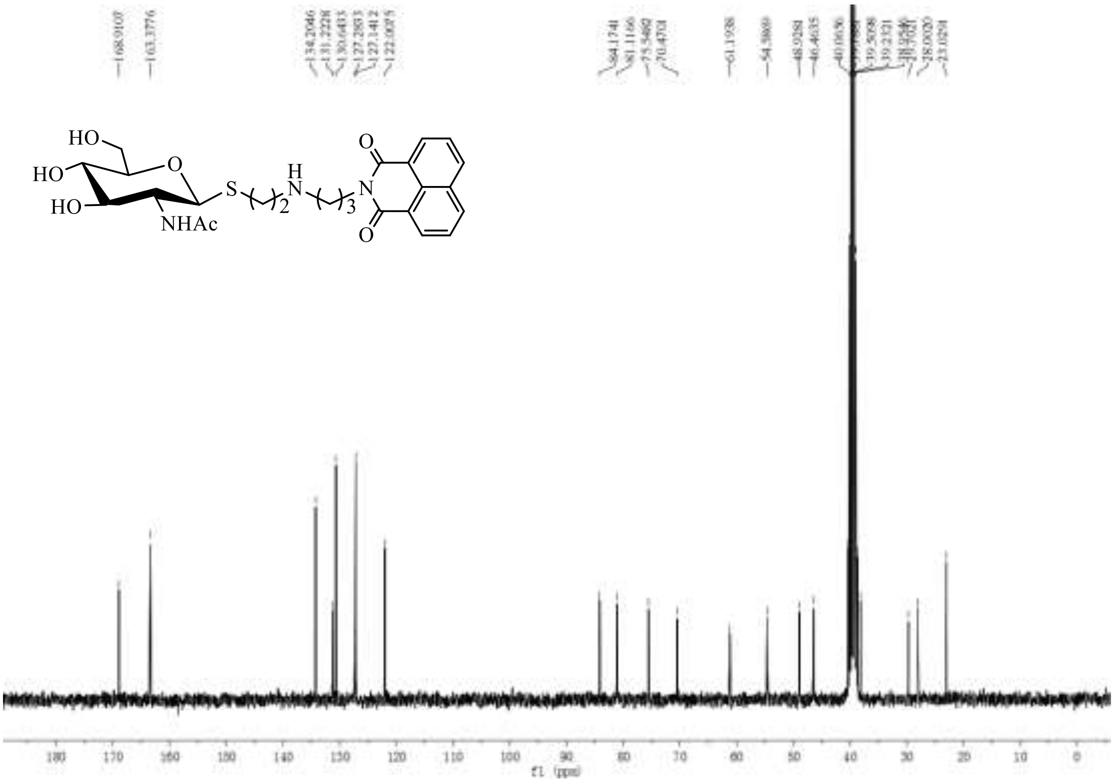

^{1}H NMR spectrum of compound 15c

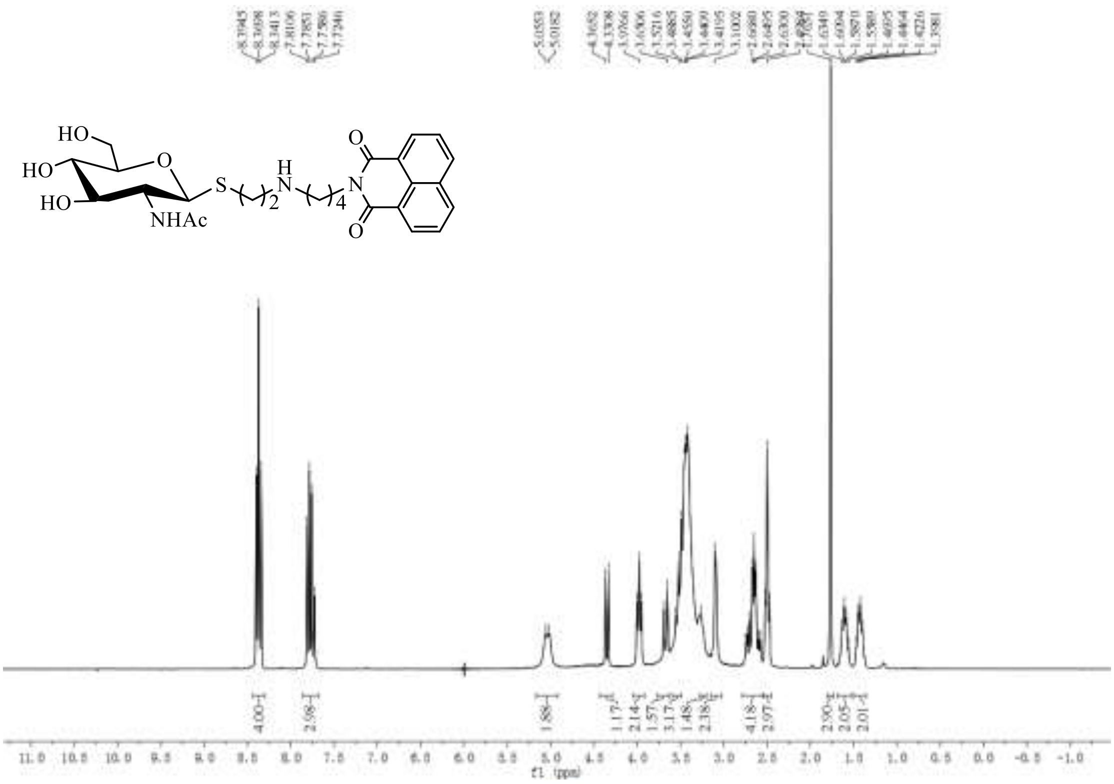

^{13}C NMR spectrum of compound 15c

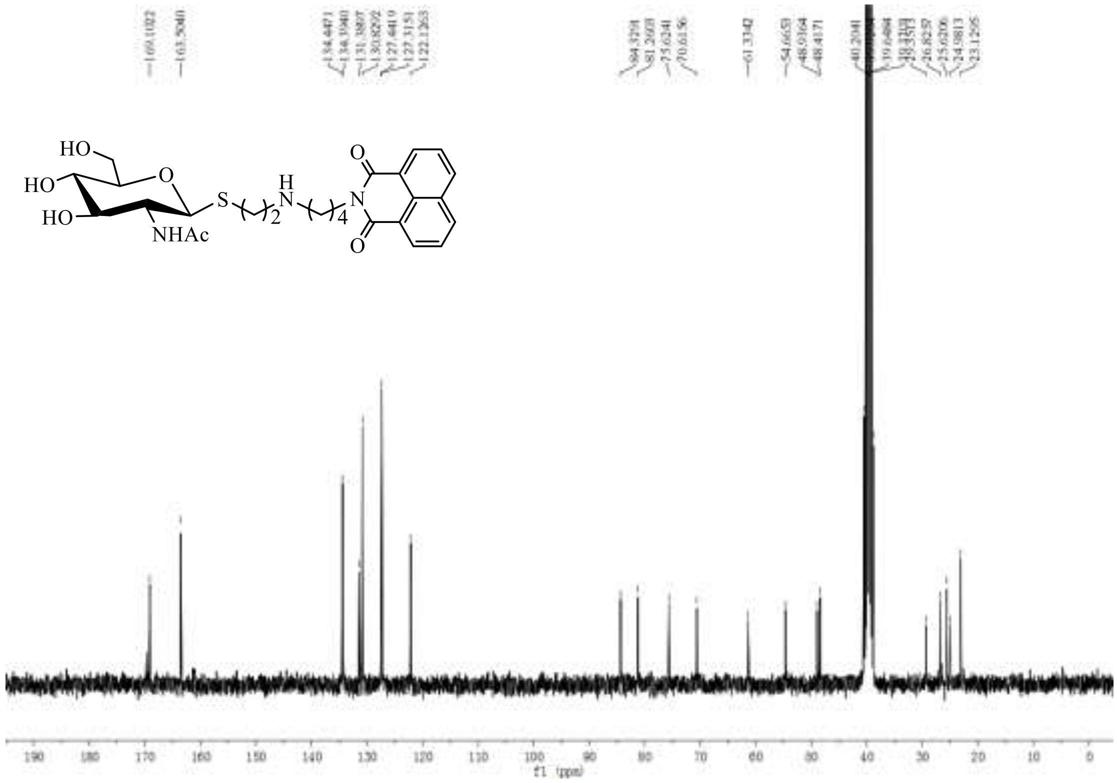

^{1}H NMR spectrum of compound 15d

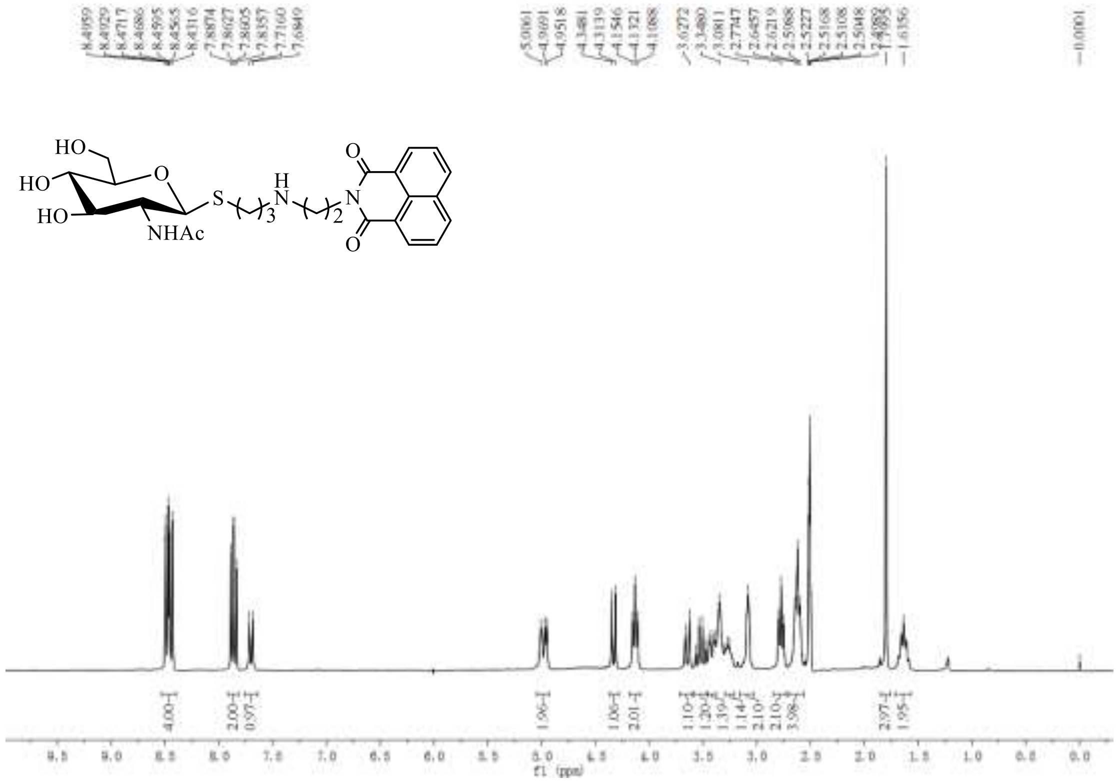

^{13}C NMR spectrum of compound 15d

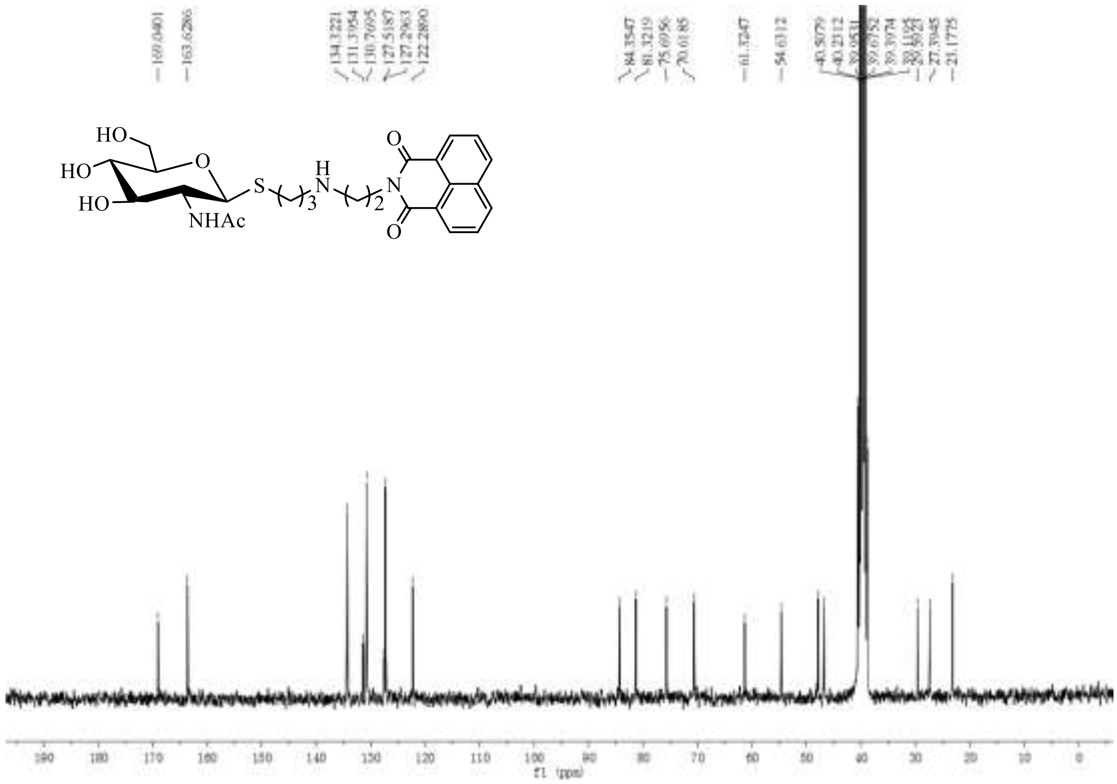

^{1}H NMR spectrum of compound 15e

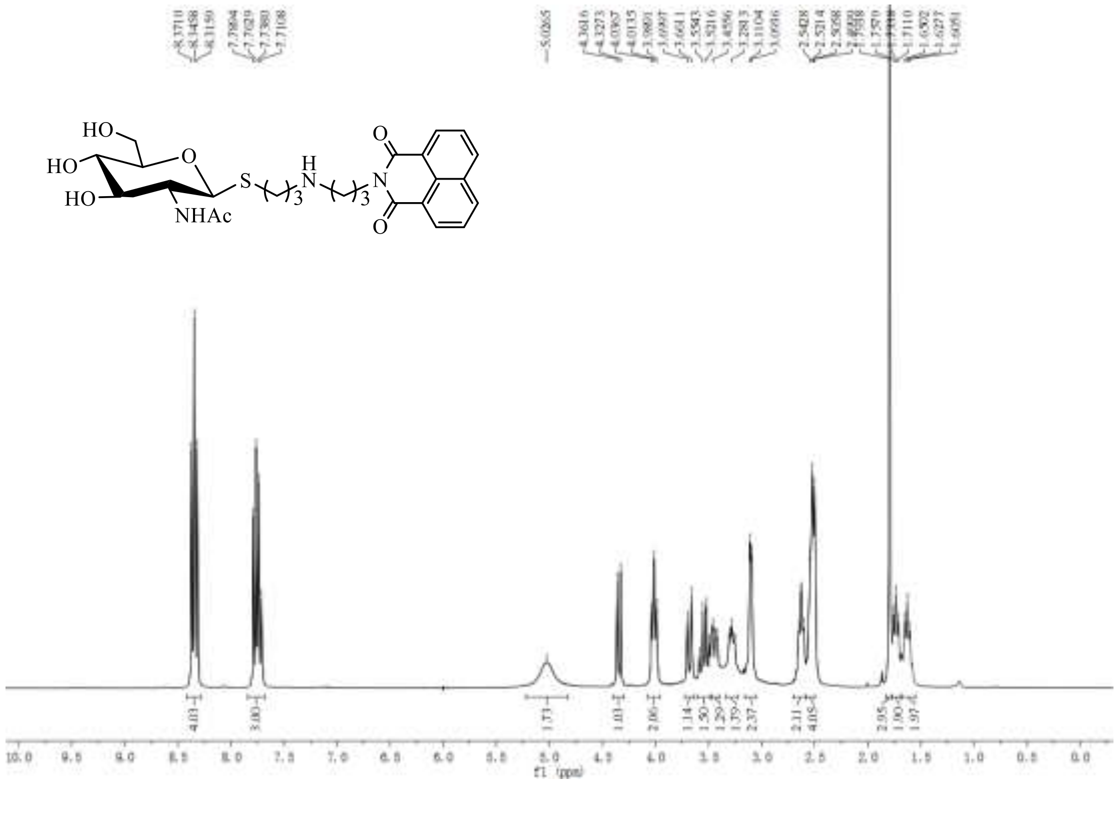

^{13}C NMR spectrum of compound 15e

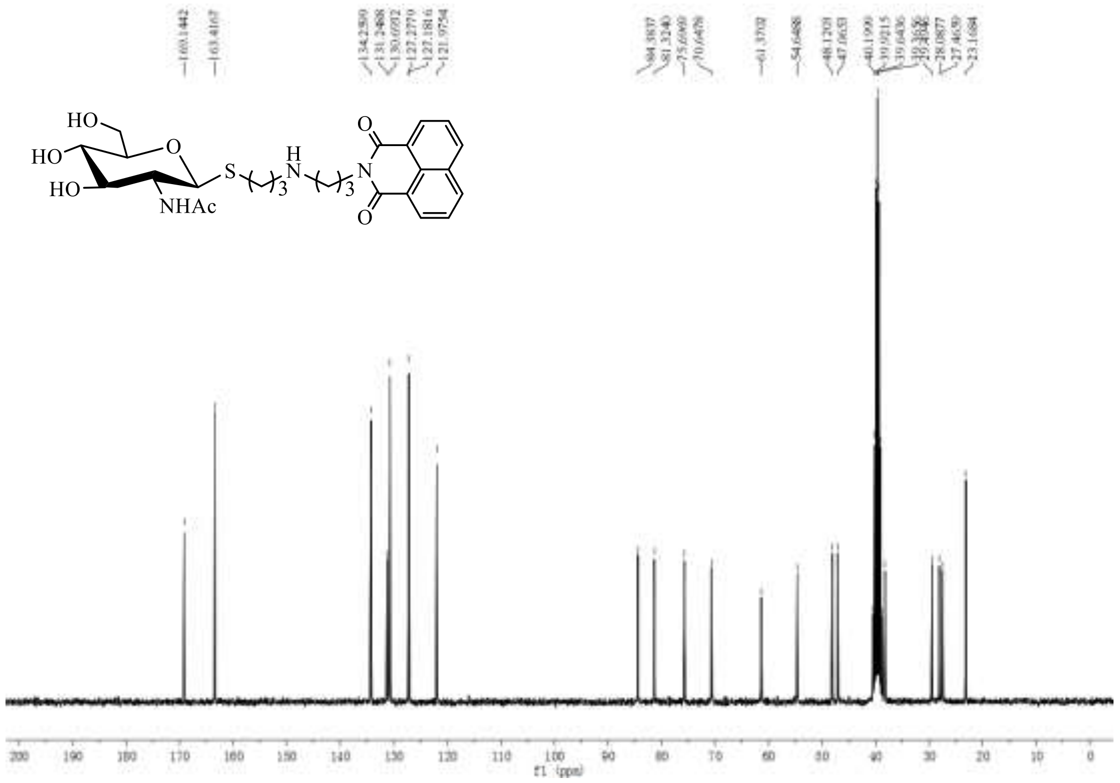

^{1}H NMR spectrum of compound 15f

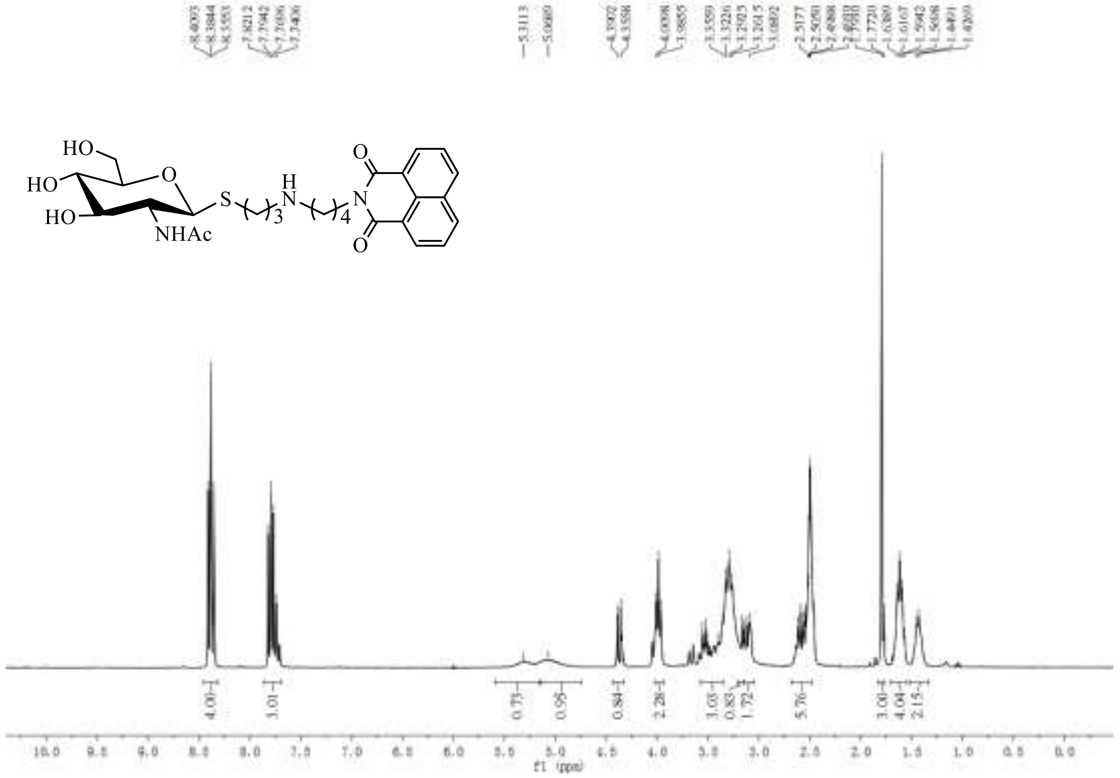

^{13}C NMR spectrum of compound 15f

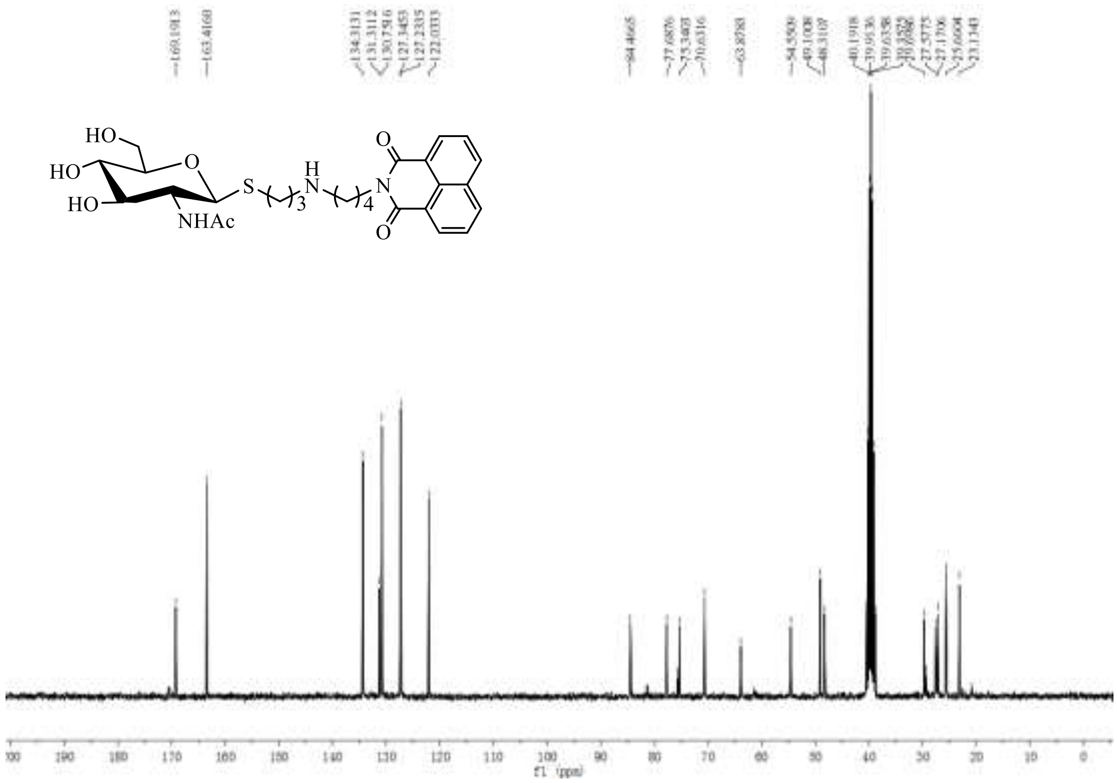

^{1}H NMR spectrum of compound 15g

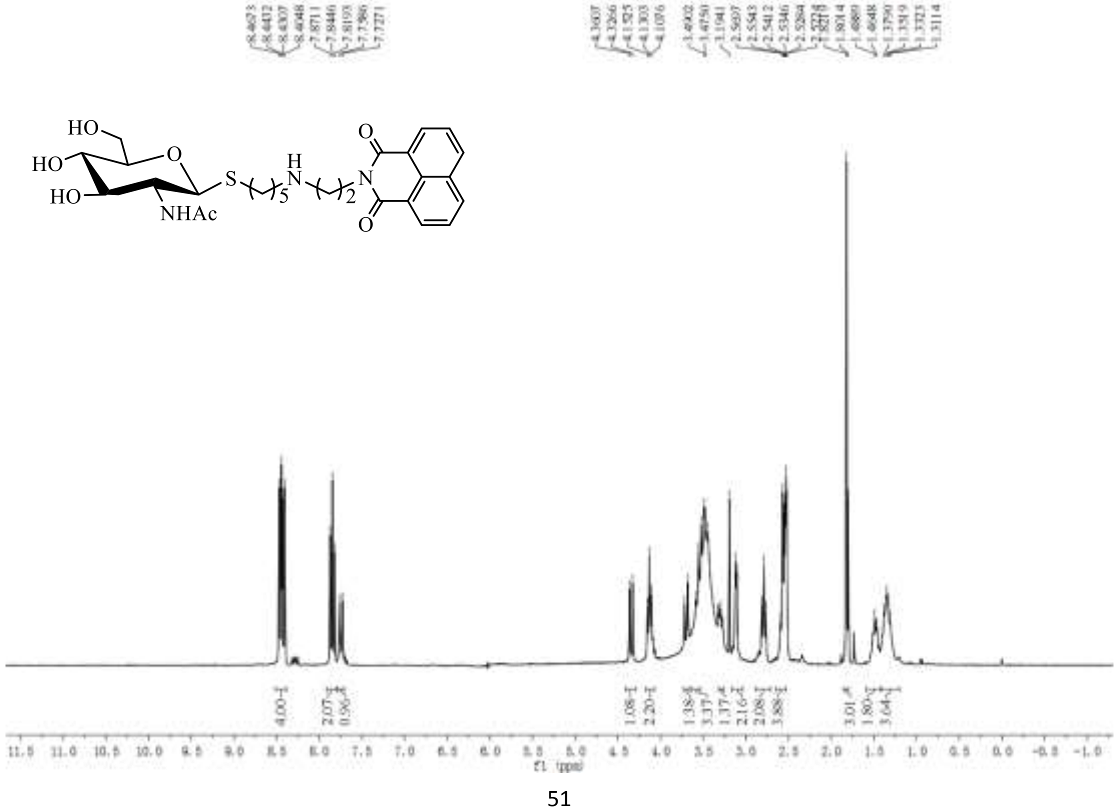

^{13}C NMR spectrum of compound 15g

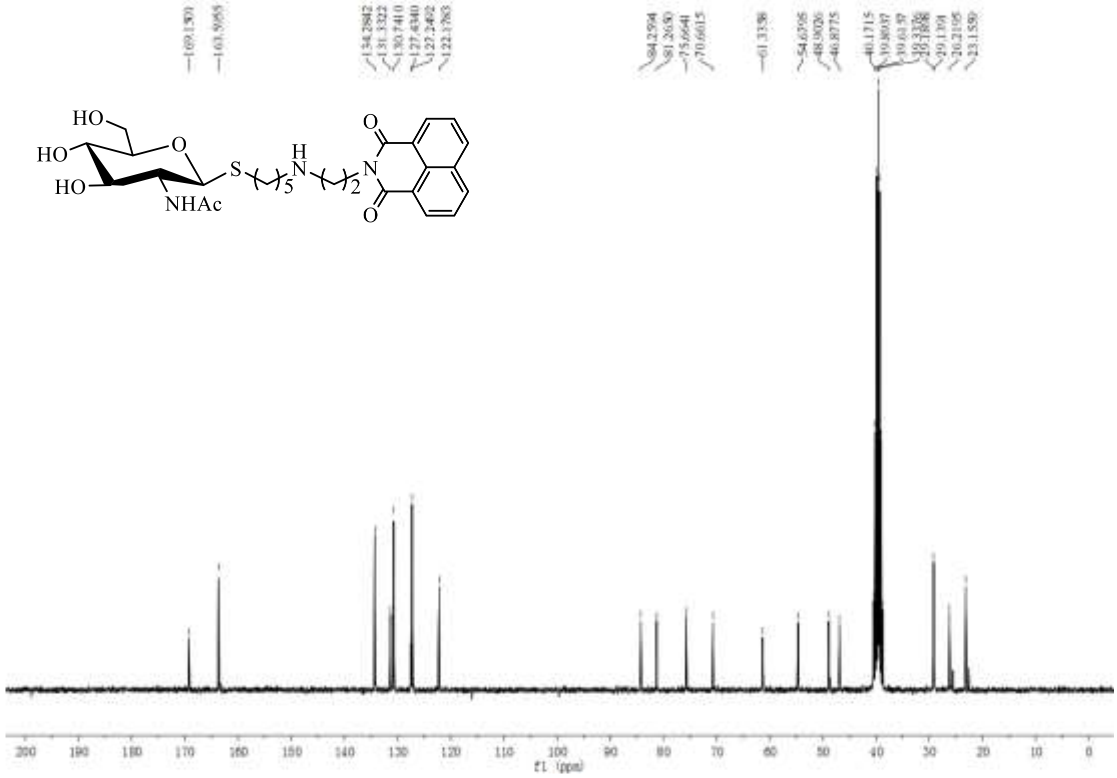

^{1}H NMR spectrum of compound 15h

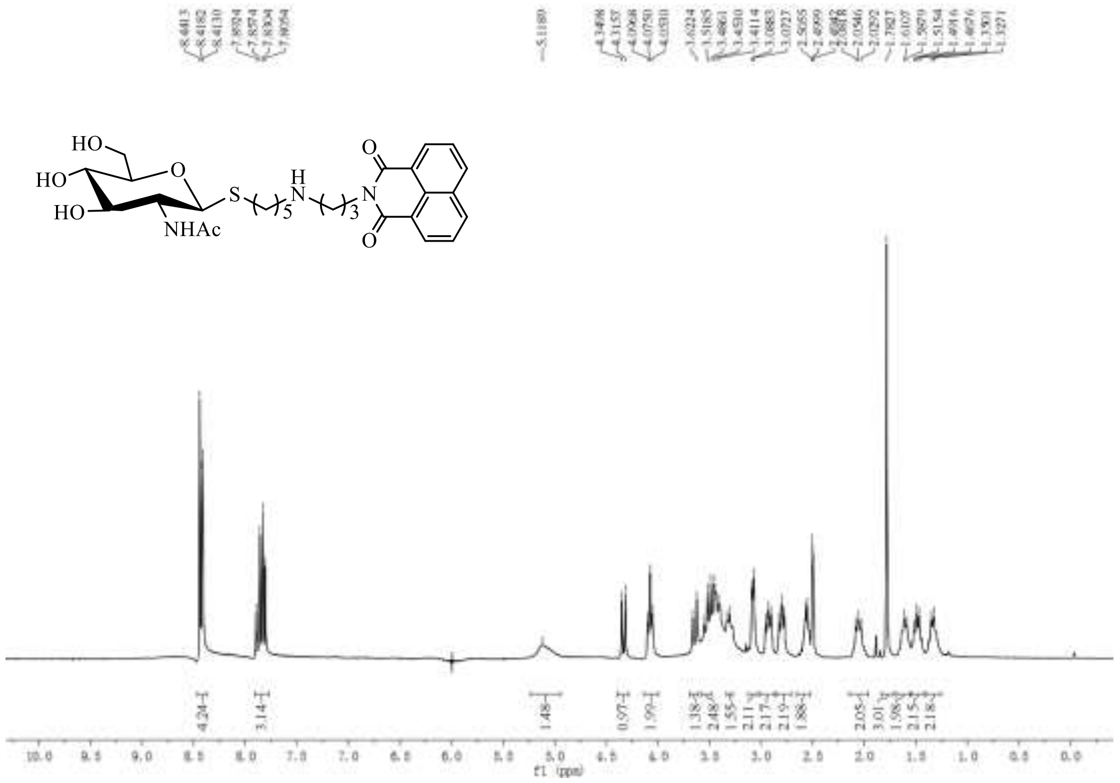

^{13}C NMR spectrum of compound 15h

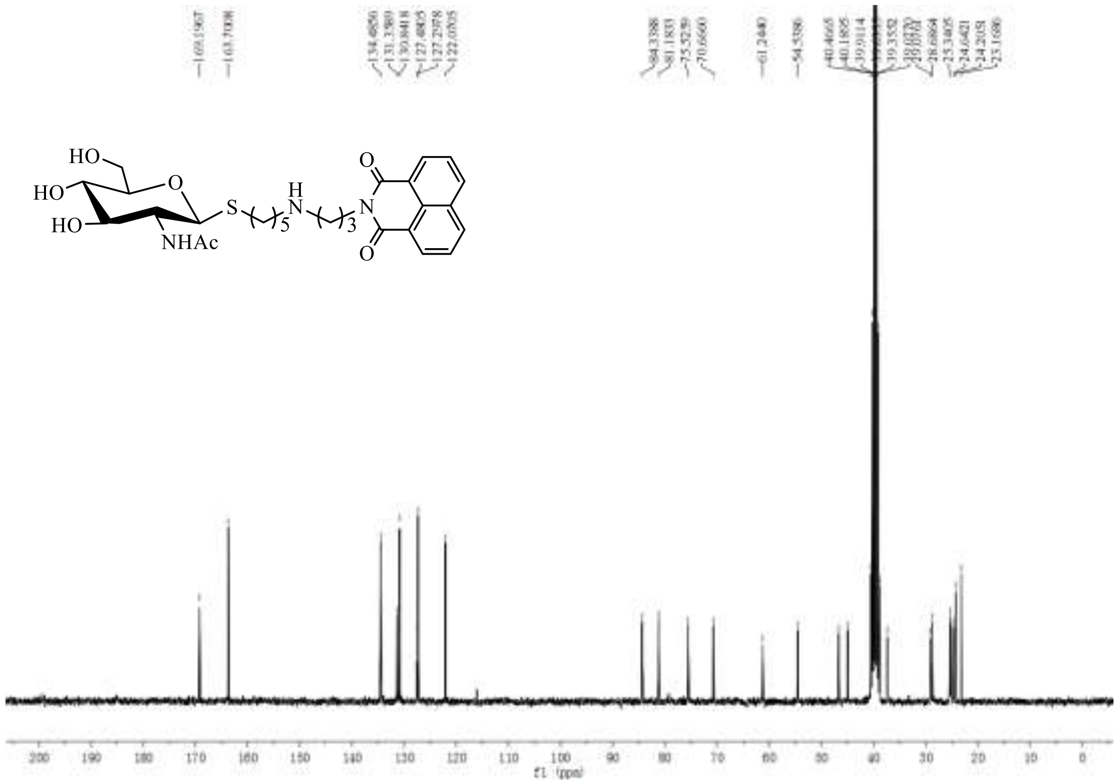

^{1}H NMR spectrum of compound 15i

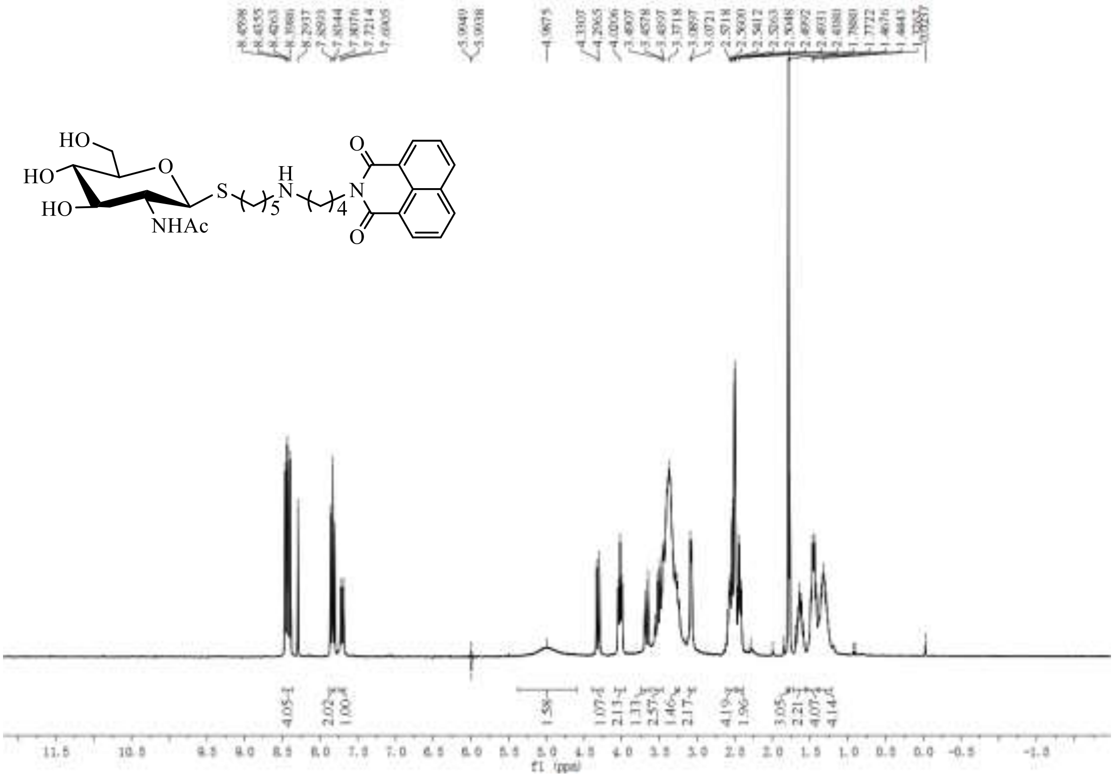

^{13}C NMR spectrum of compound 15i

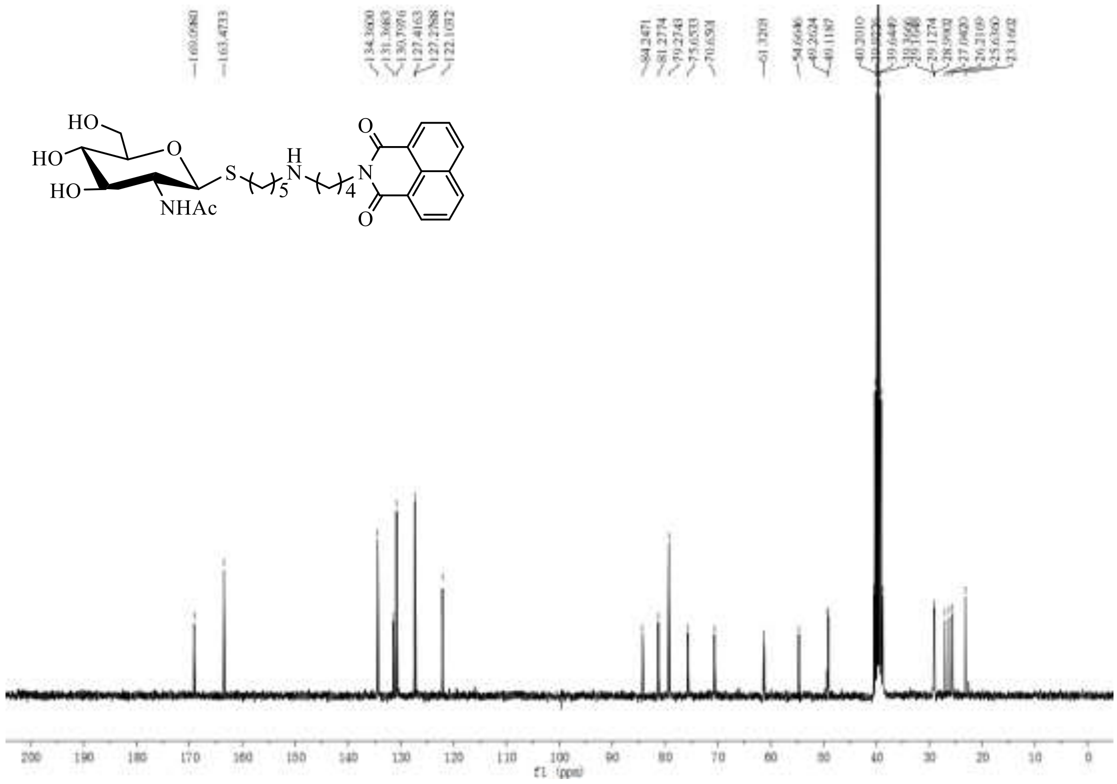

^{1}H NMR spectrum of compound 15j

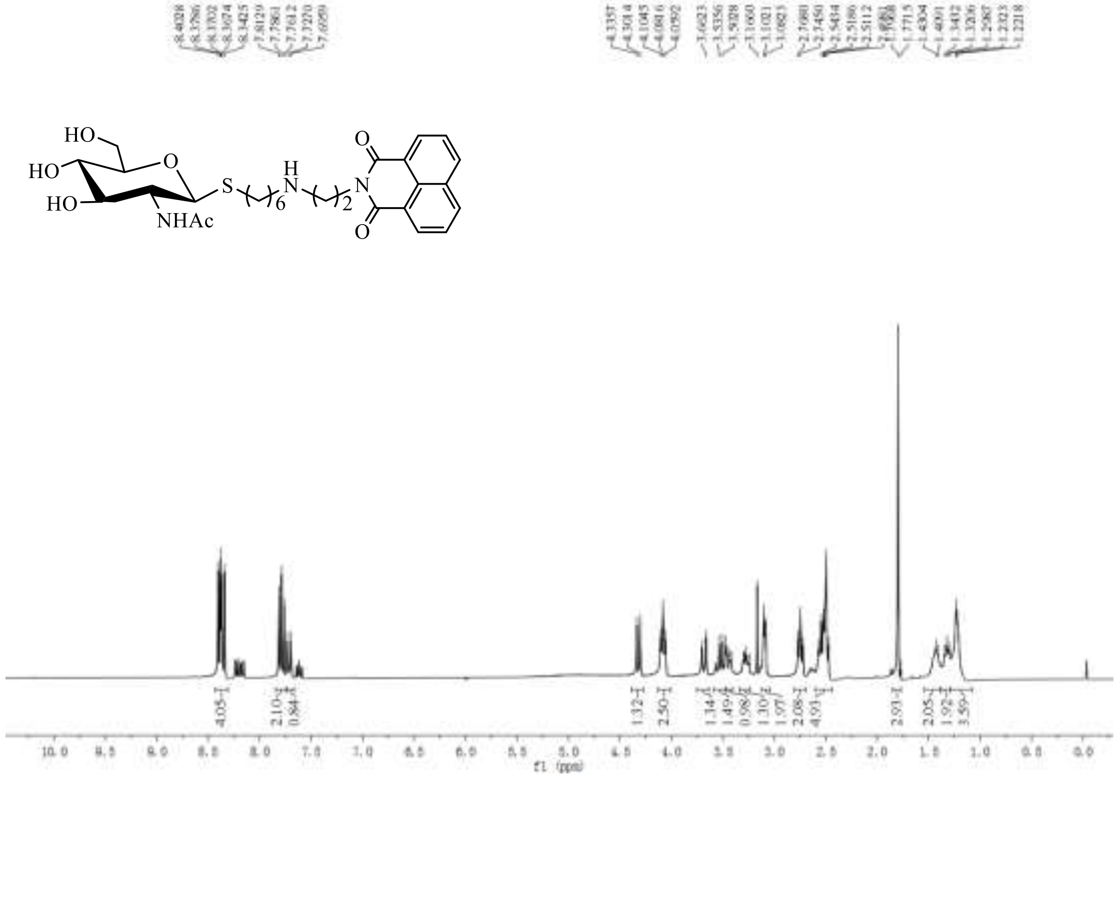

^{13}C NMR spectrum of compound 15j

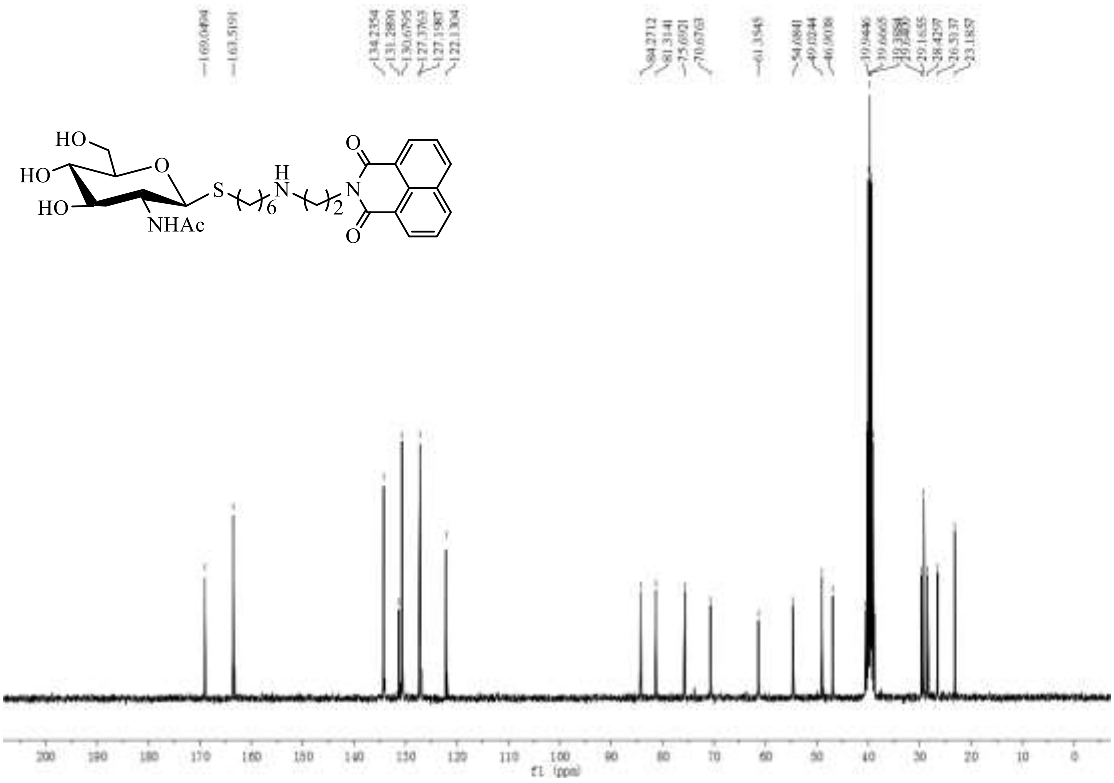

^{1}H NMR spectrum of compound 15k

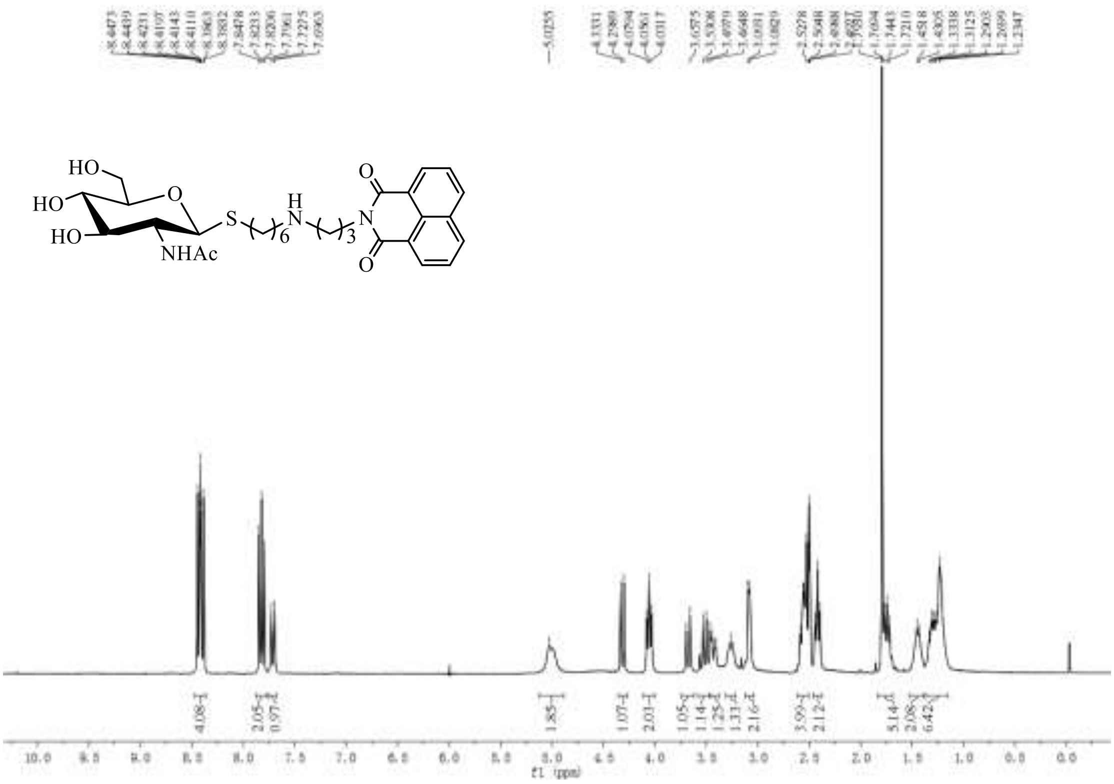

^{13}C NMR spectrum of compound 15k

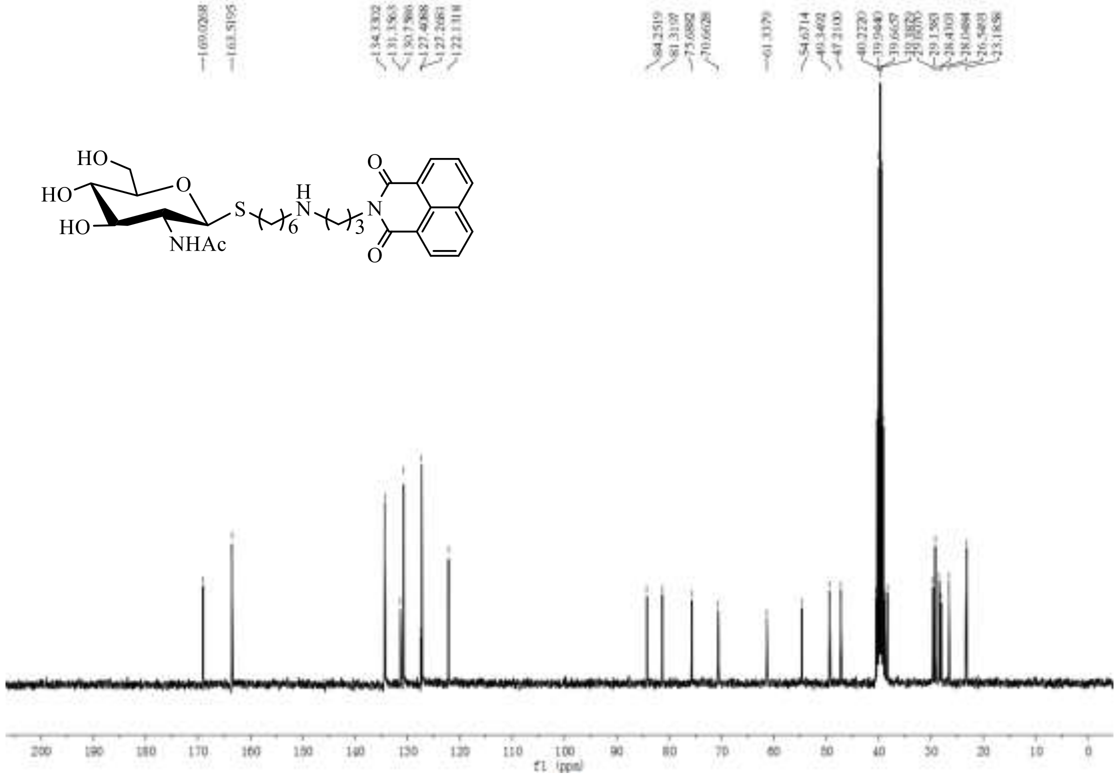

^{1}H NMR spectrum of compound 15l

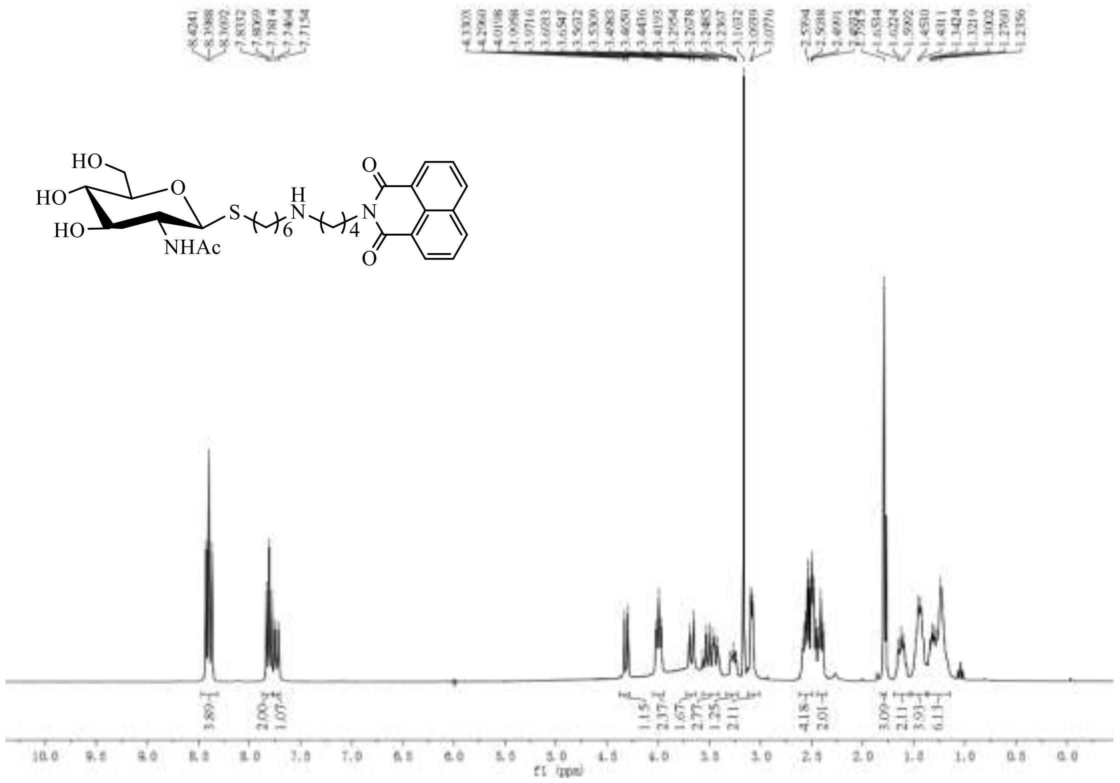

^{13}C NMR spectrum of compound 15l

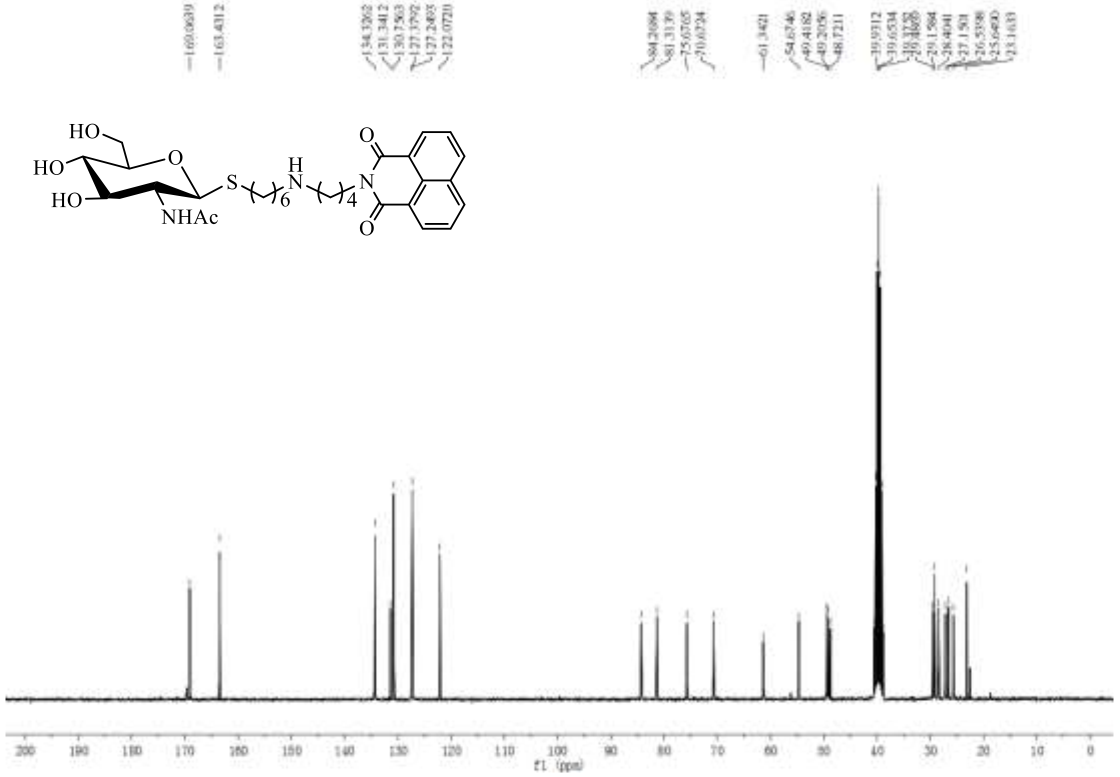

^{1}H NMR spectrum of compound 17

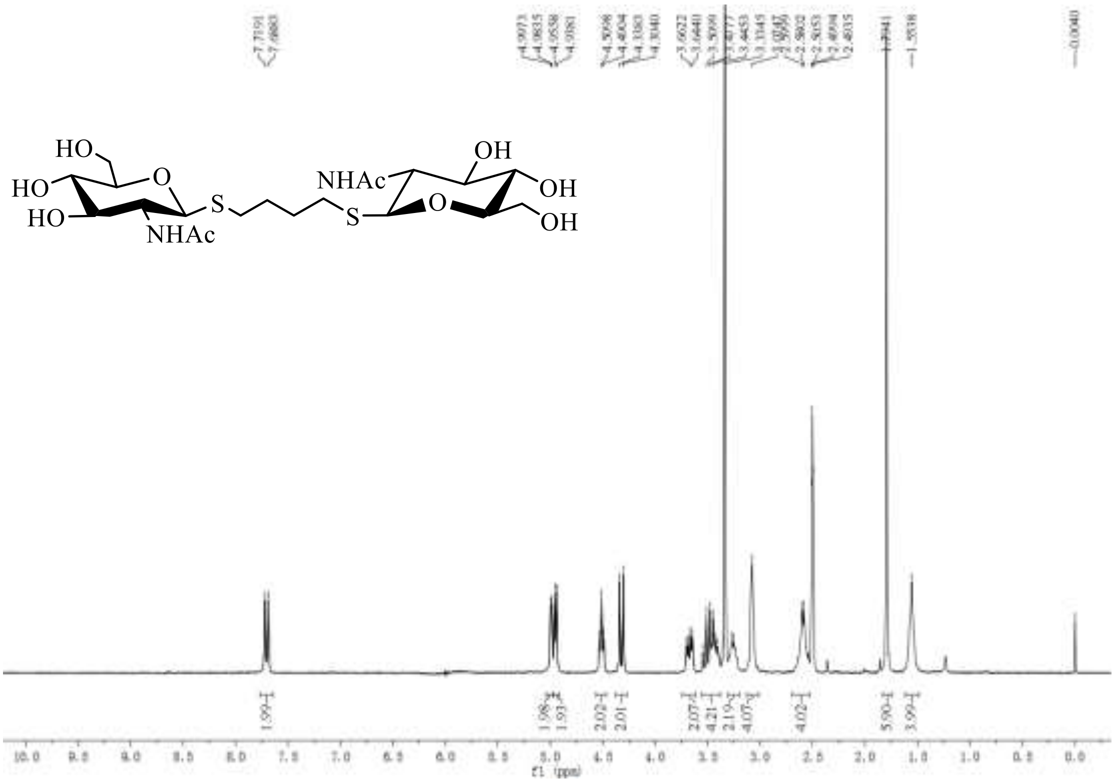

^{13}C NMR spectrum of compound 17

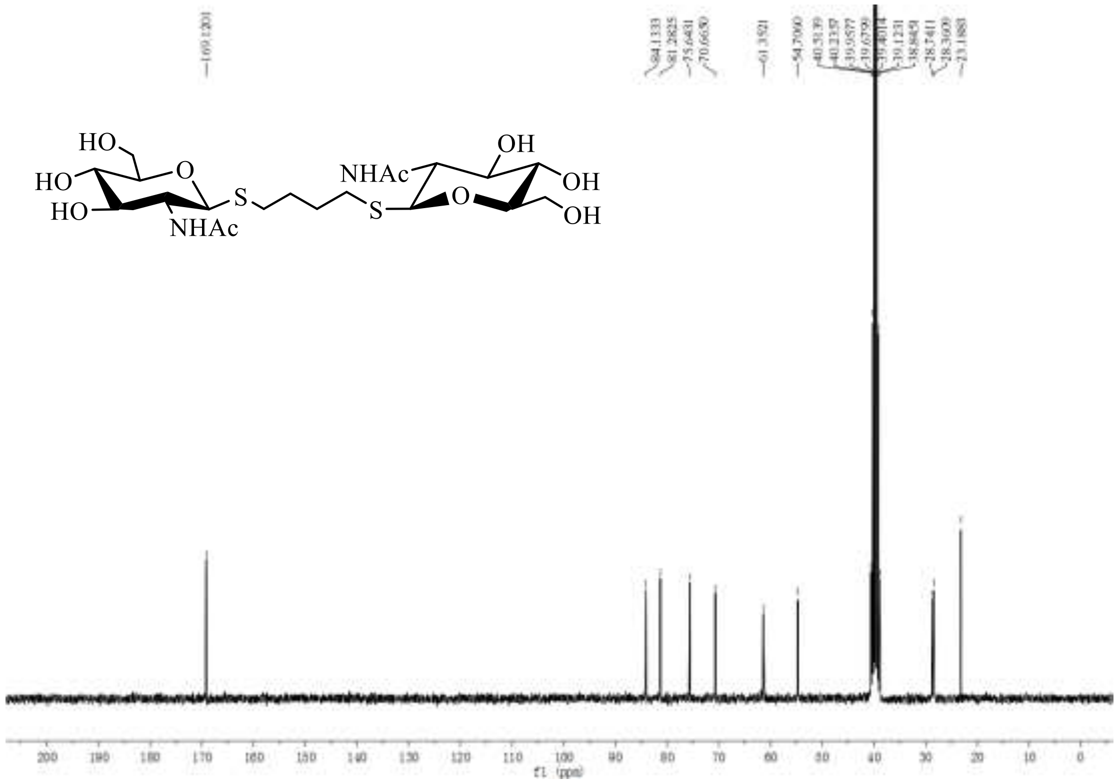

^{1}H NMR spectrum of compound 20a

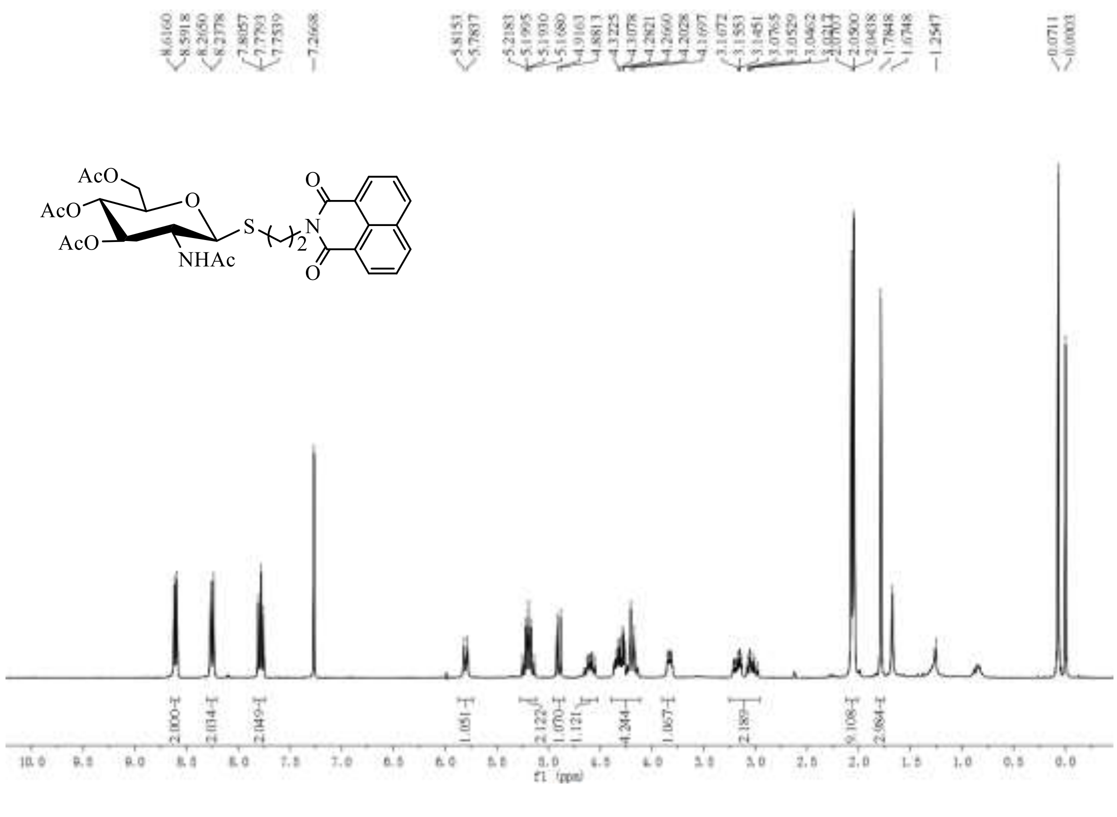

^{1}H NMR spectrum of compound 20a

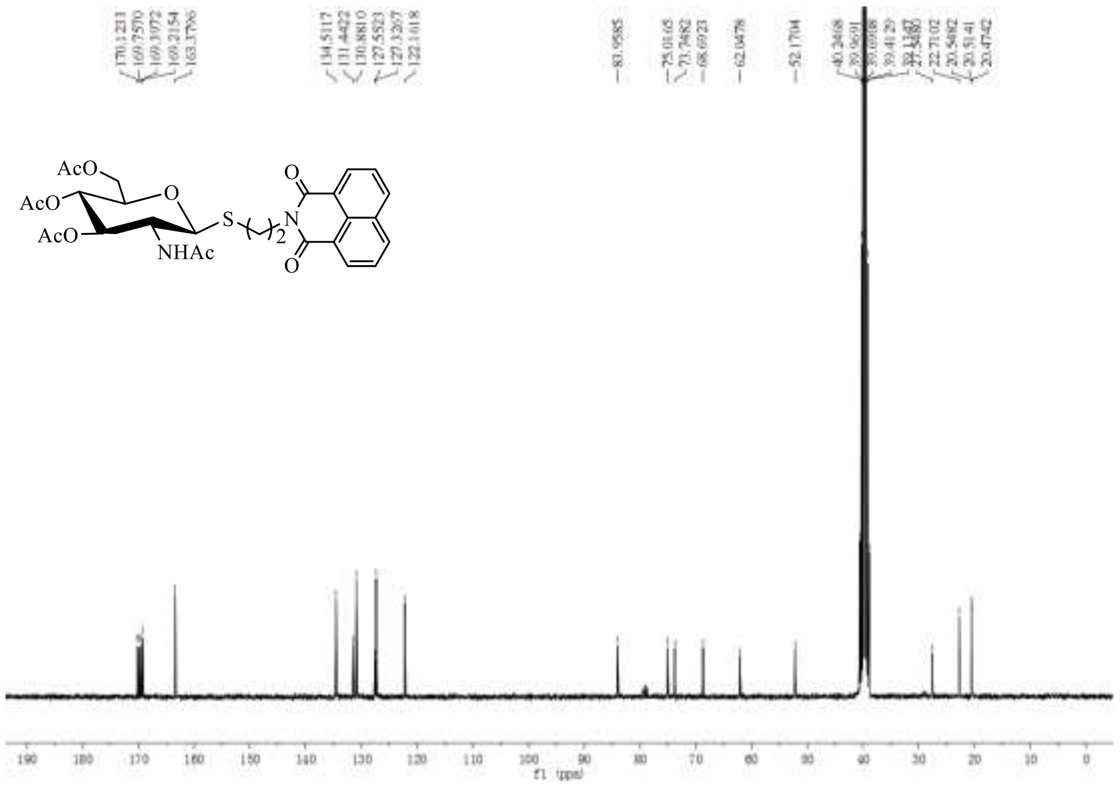

^{1}H NMR spectrum of compound 20b

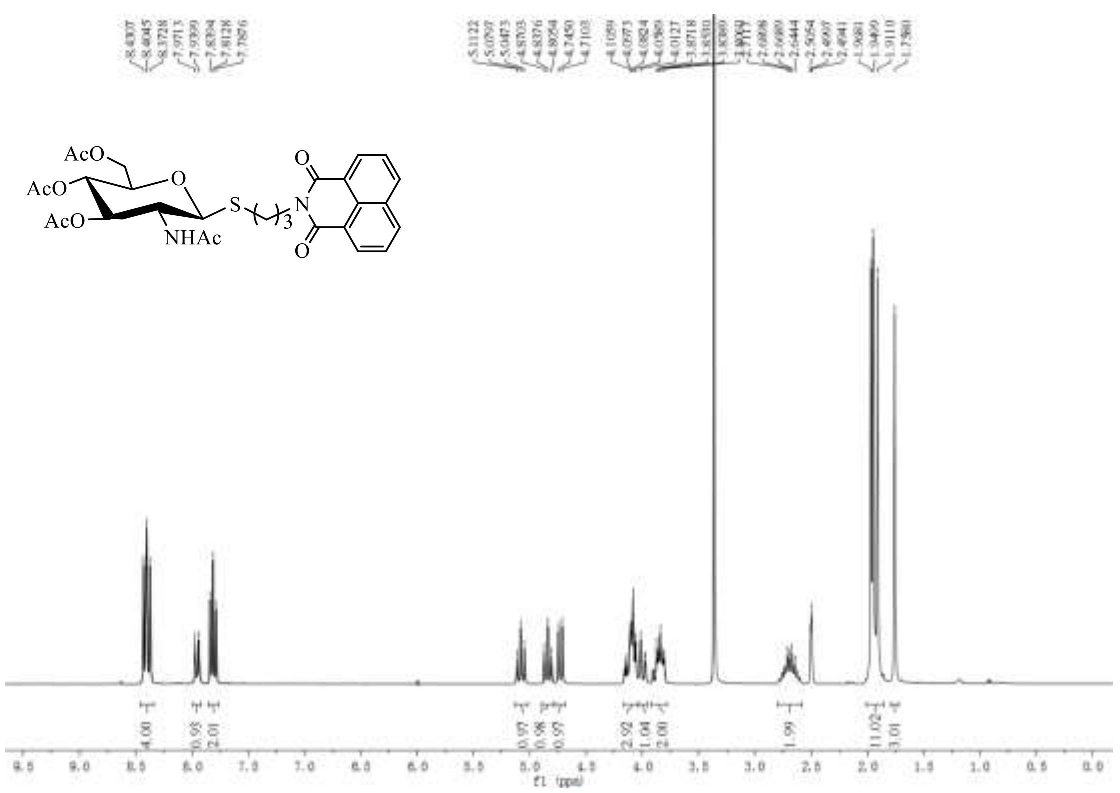

^{13}C NMR spectrum of compound 20b

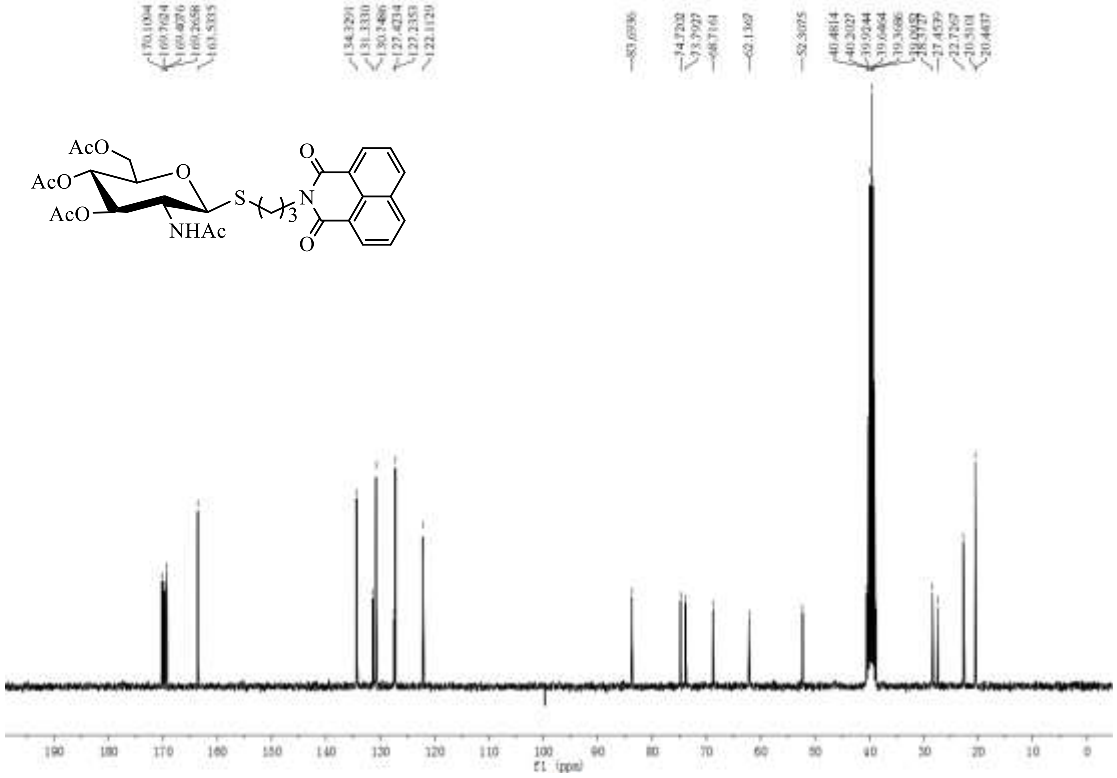

^{1}H NMR spectrum of compound 20c

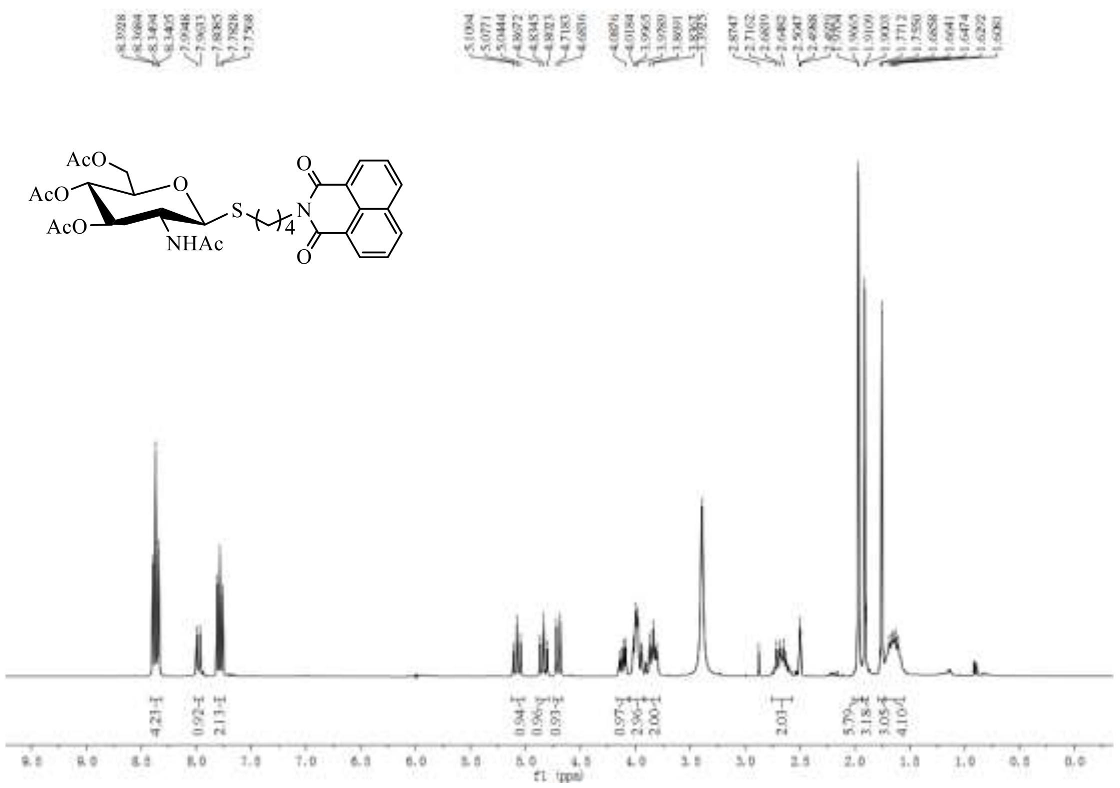

^{13}C NMR spectrum of compound 20c

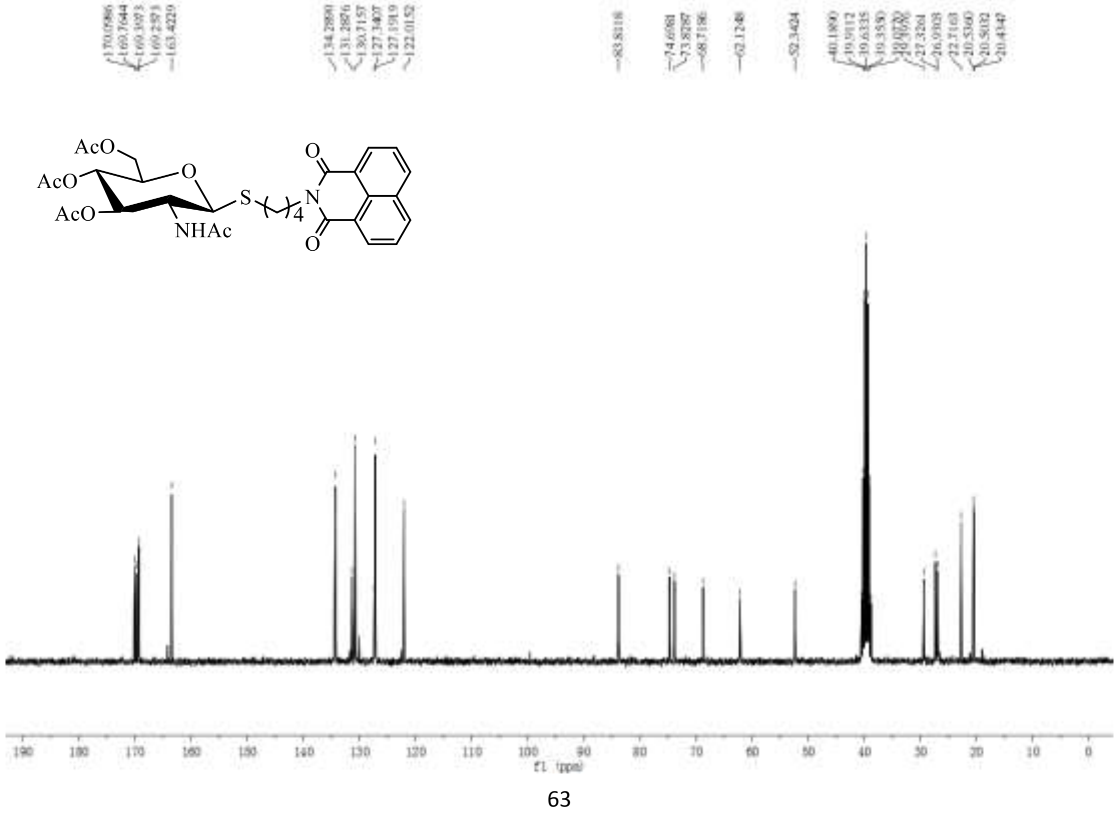

^{1}H NMR spectrum of compound 20d

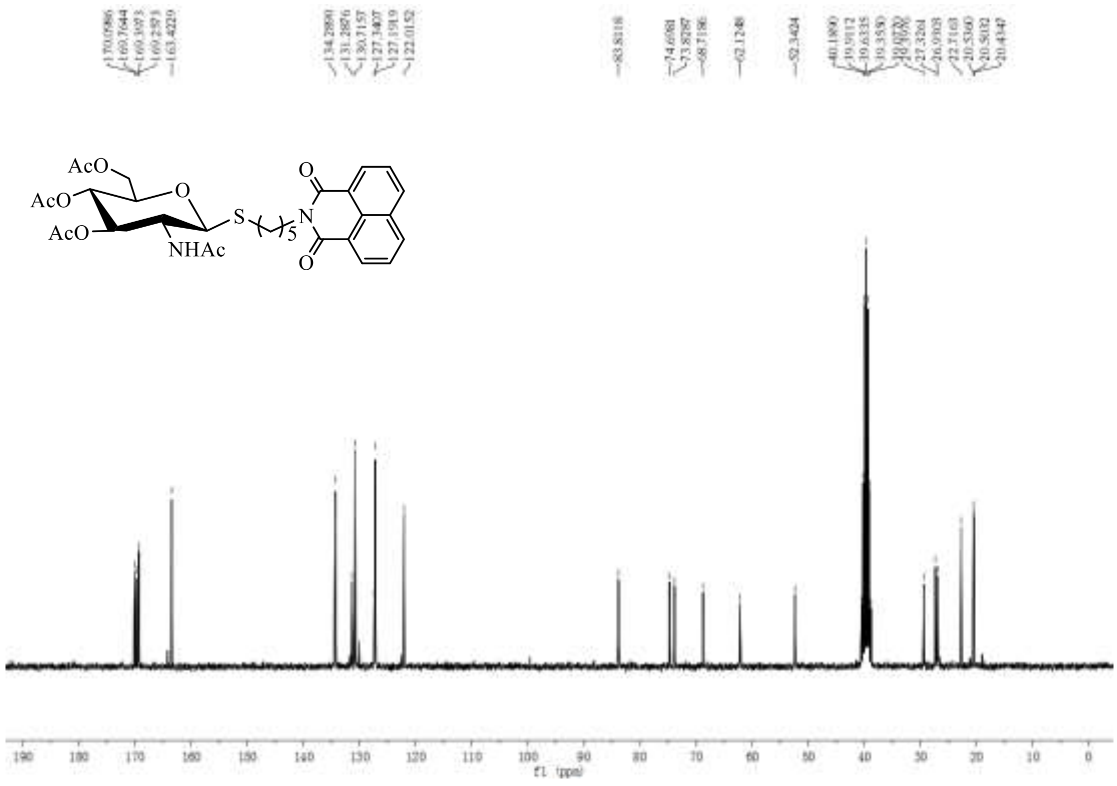

^{13}C NMR spectrum of compound 20d

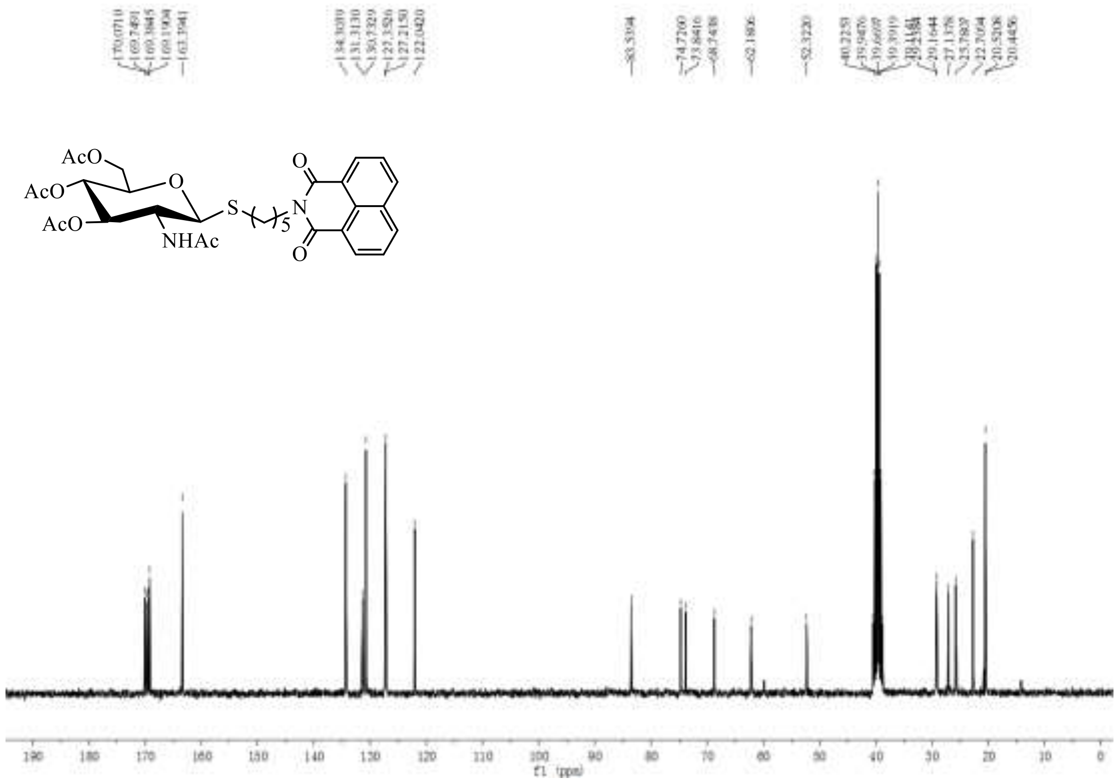

^{1}H NMR spectrum of compound 20e

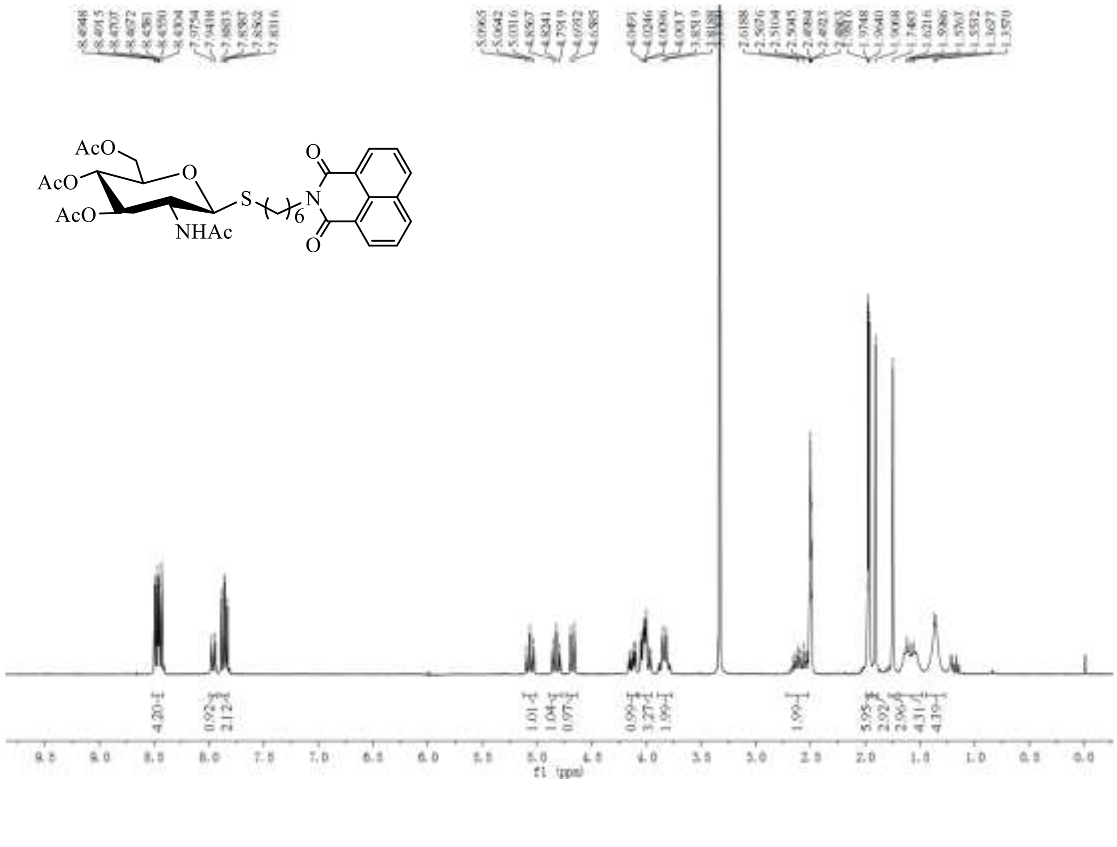

^{13}C NMR spectrum of compound 20e

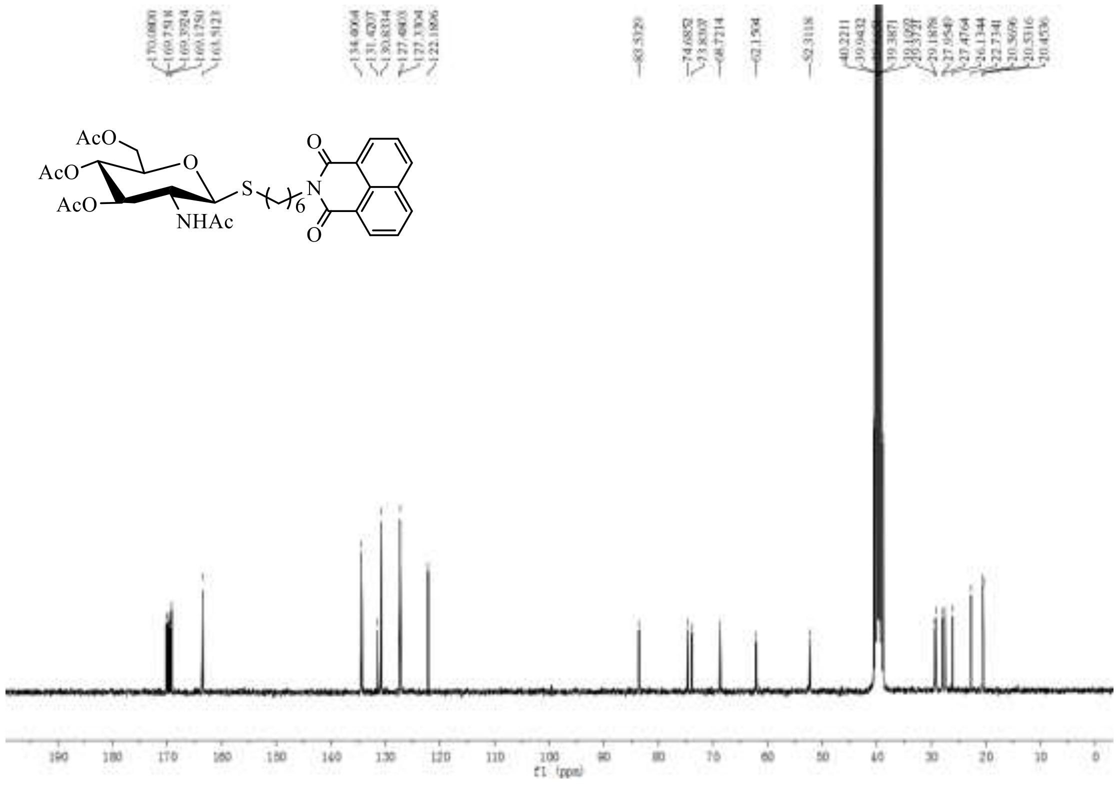

^{1}H NMR spectrum of compound 21a

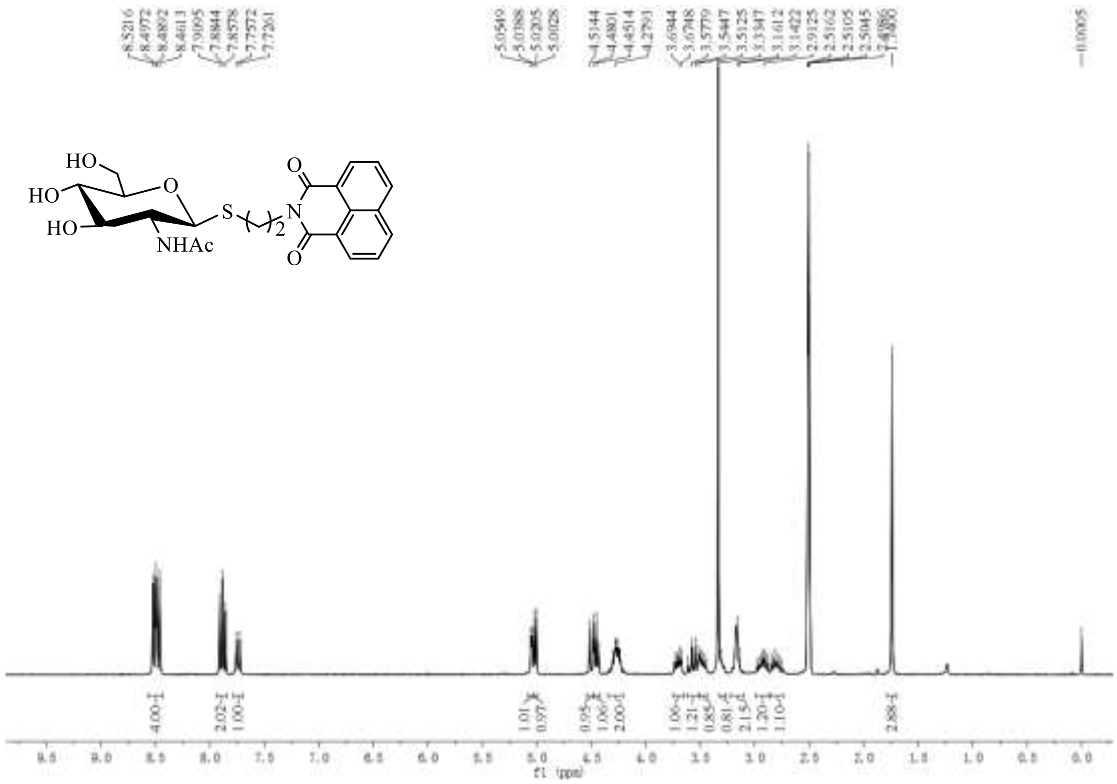

^{13}C NMR spectrum of compound 21a

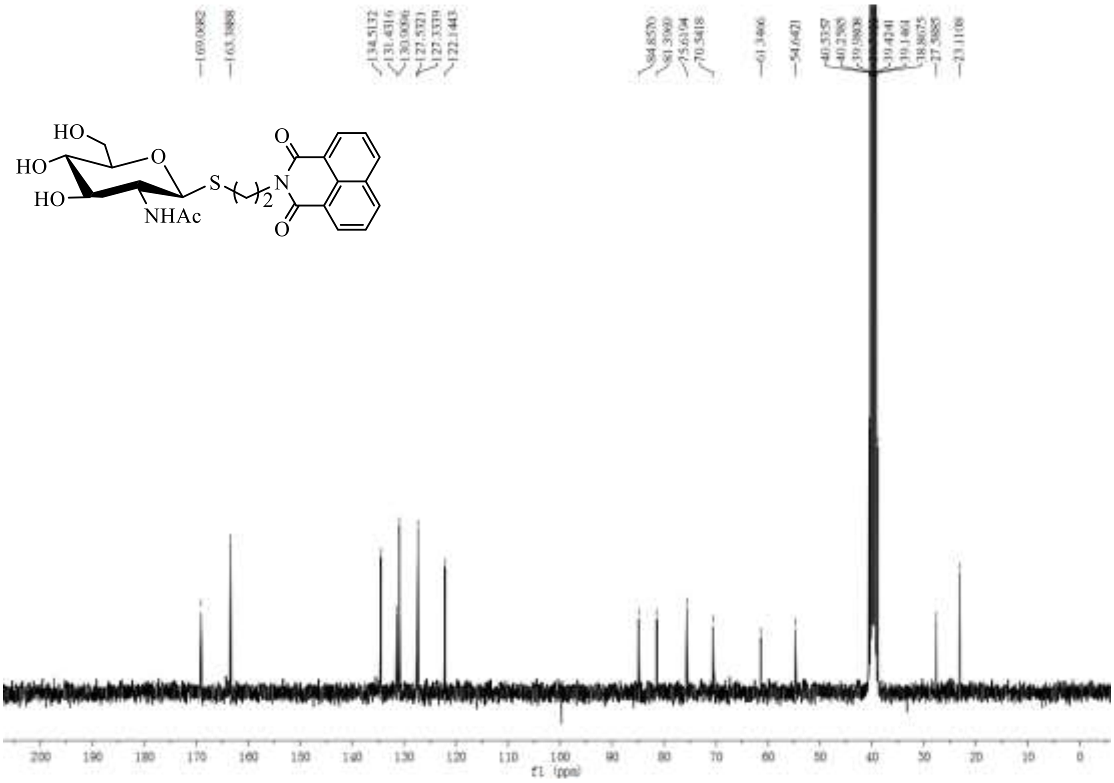

^{1}H NMR spectrum of compound 21b

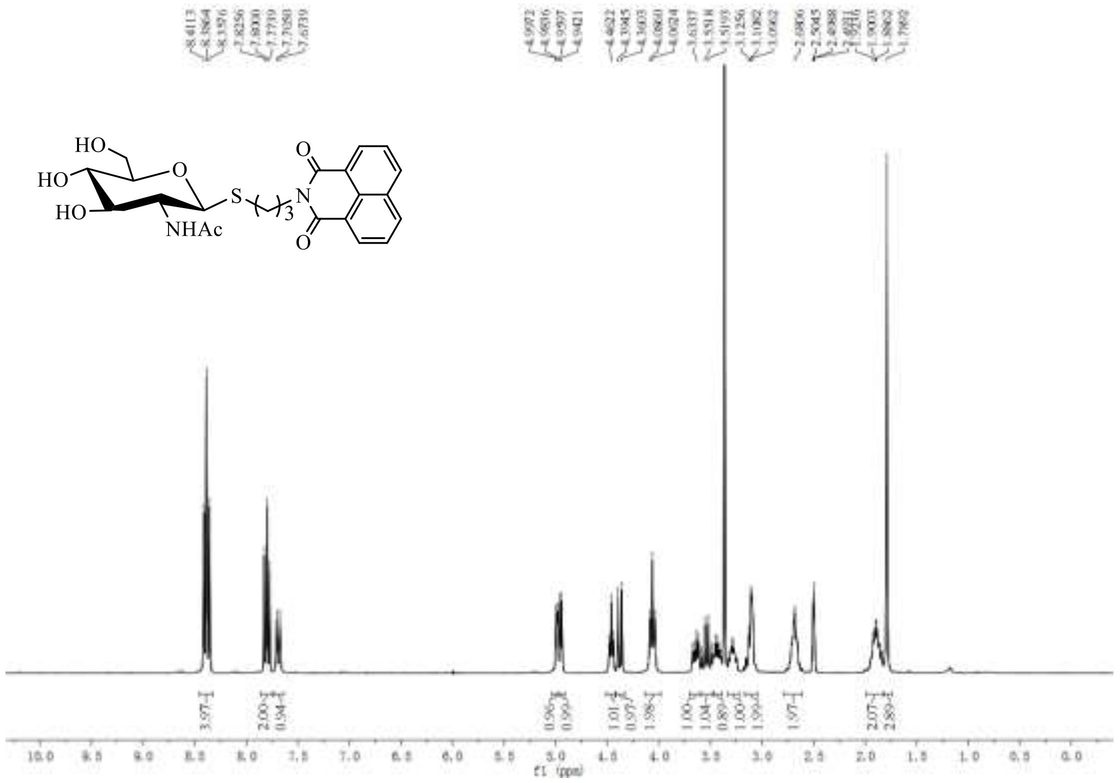

^{13}C NMR spectrum of compound 21b

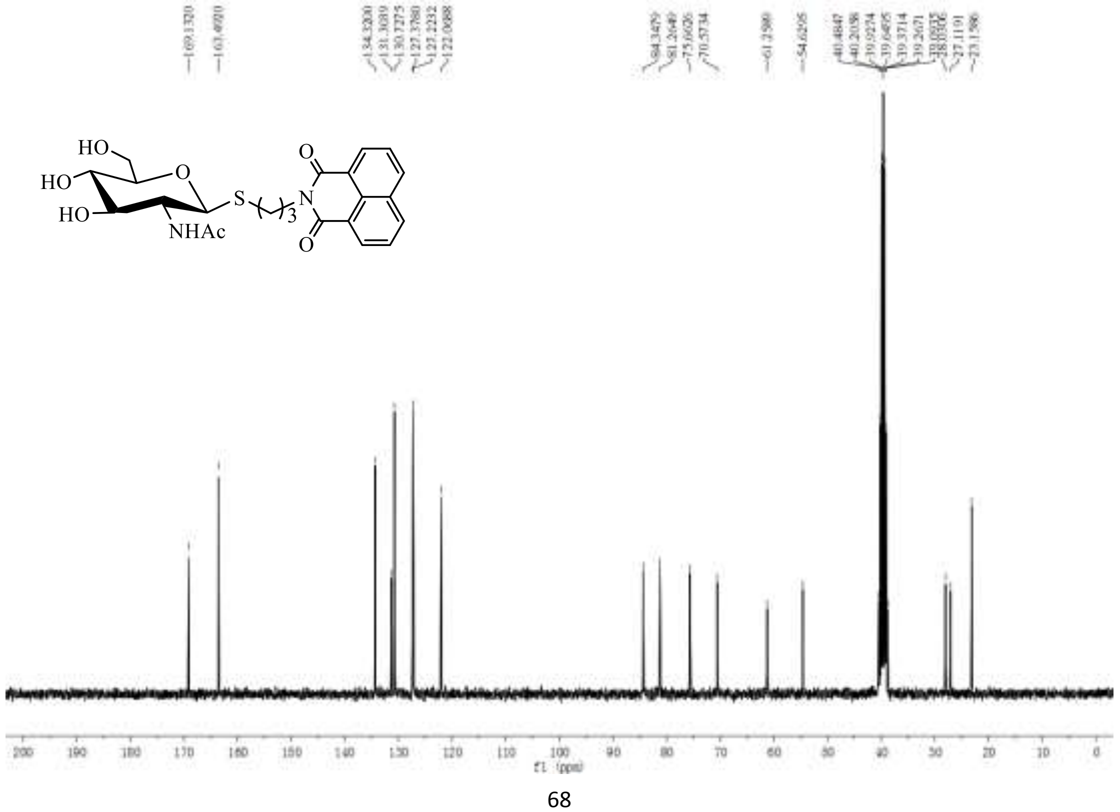

^{1}H NMR spectrum of compound 21c

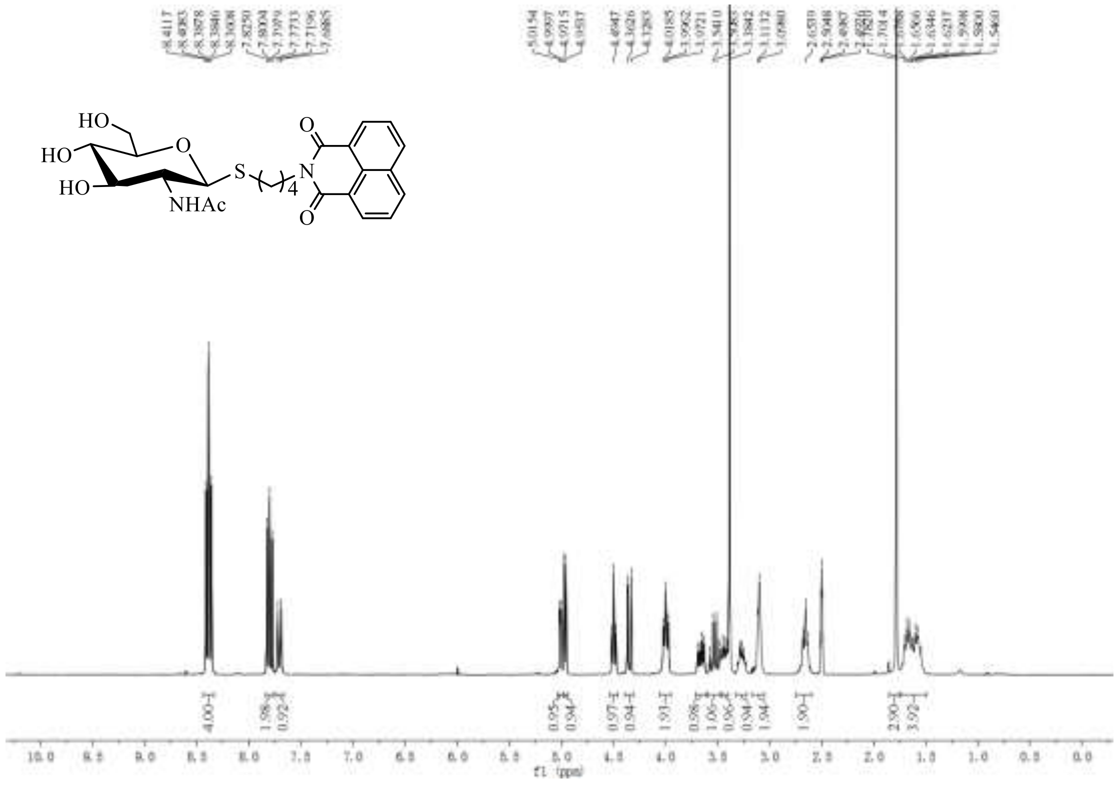

^{13}C NMR spectrum of compound 21c

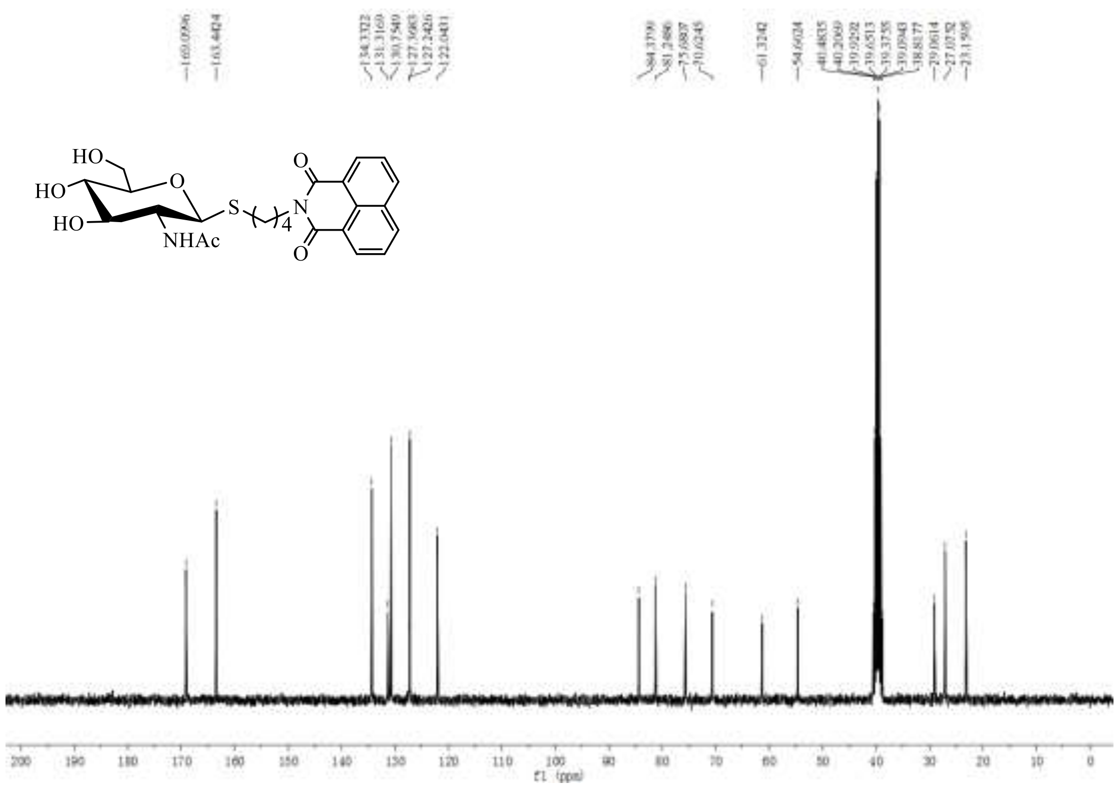

^{1}H NMR spectrum of compound 21d

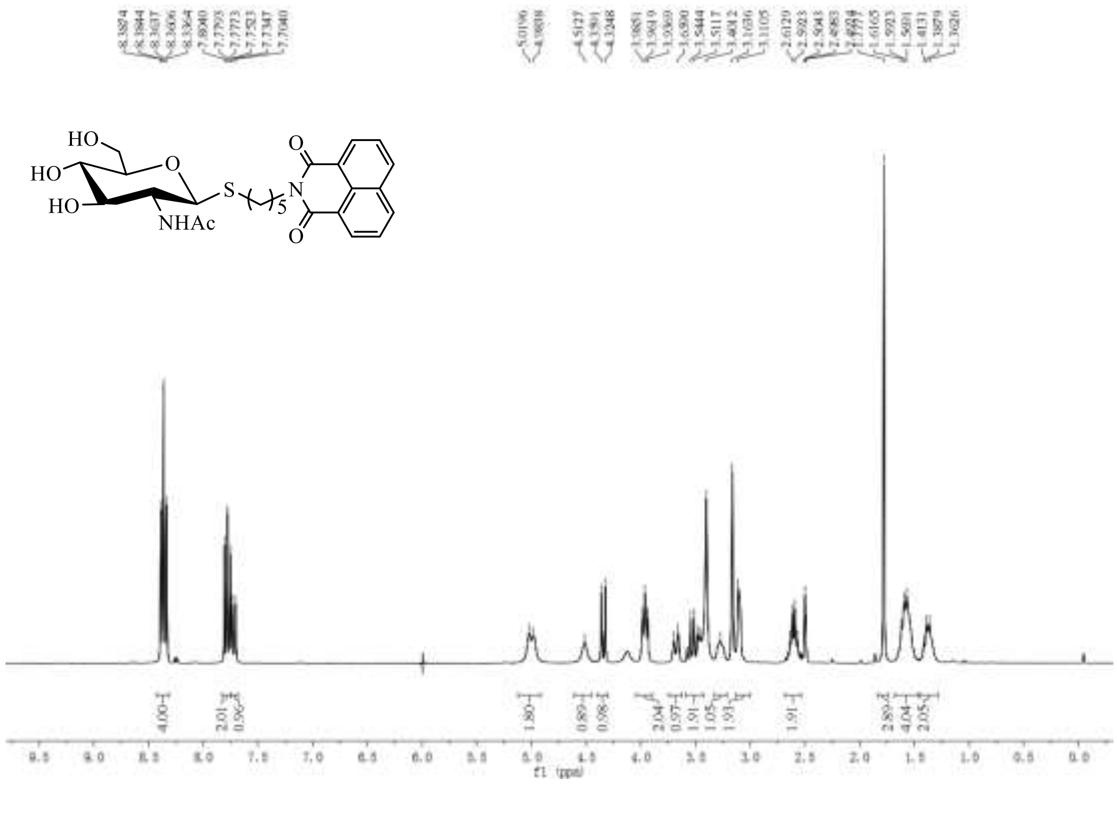

^{13}C NMR spectrum of compound 21d

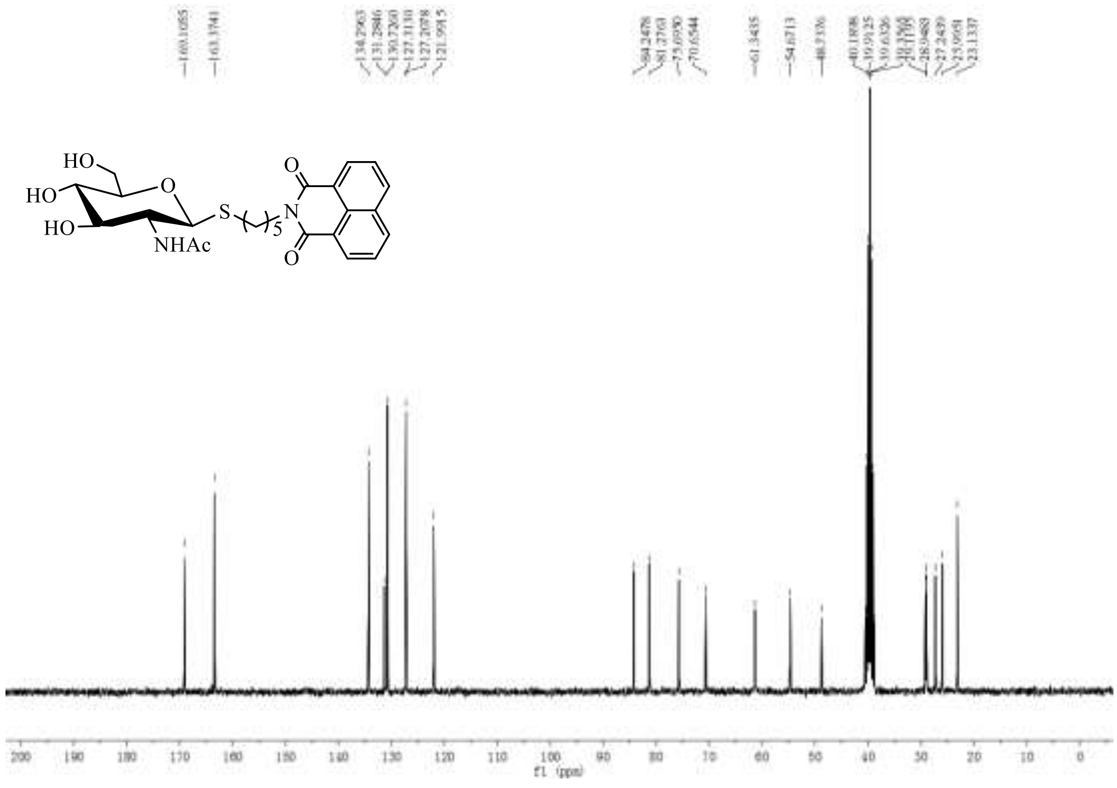

^{1}H NMR spectrum of compound 21e

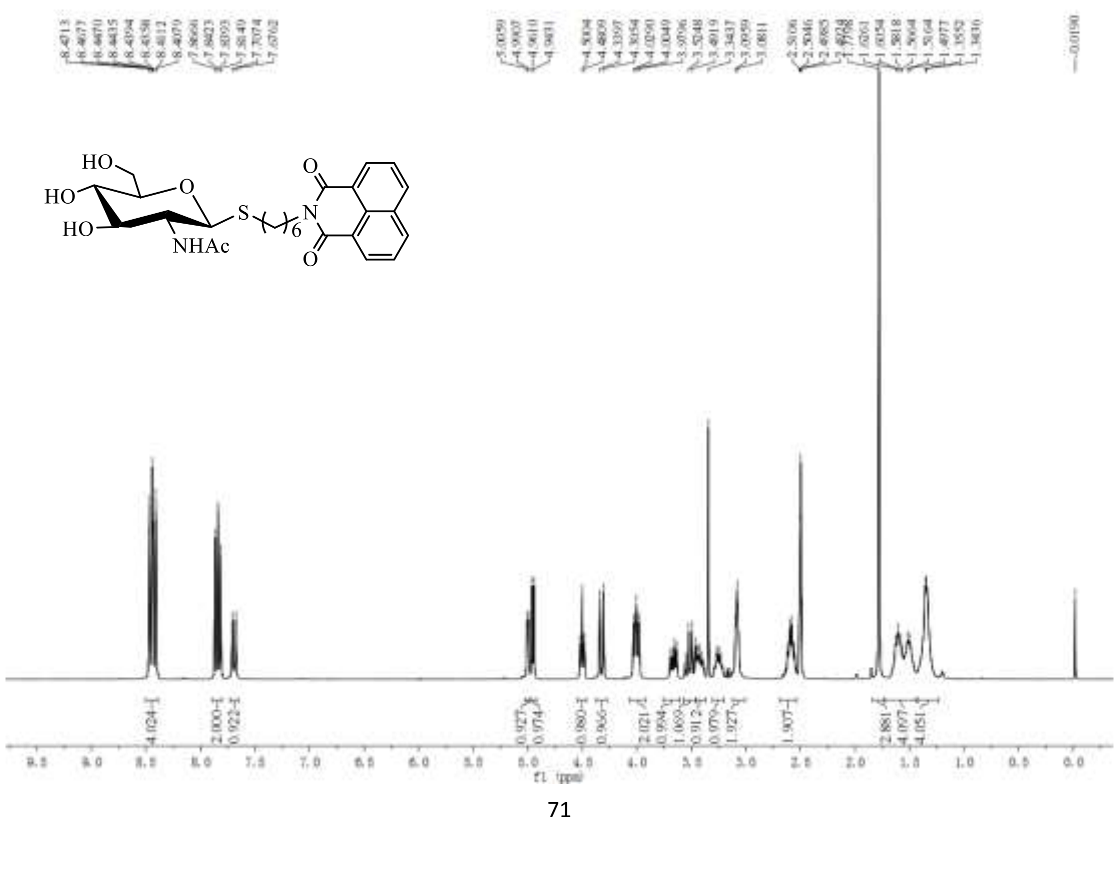

^{13}C NMR spectrum of compound 21e

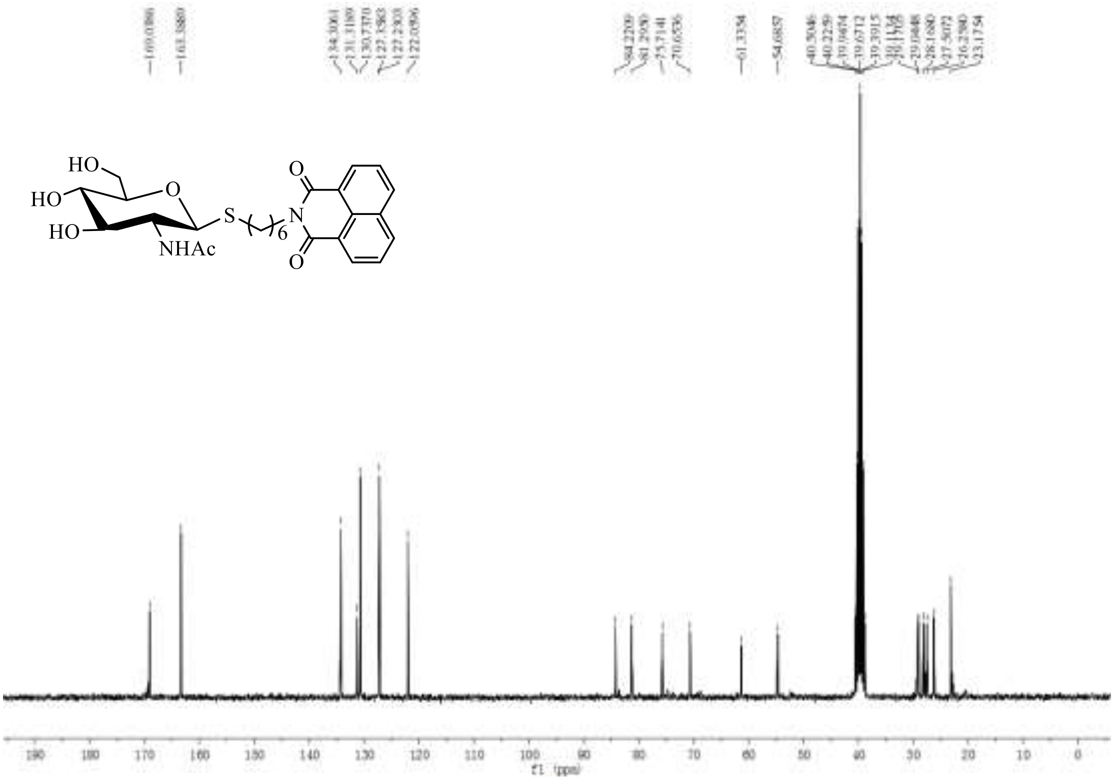

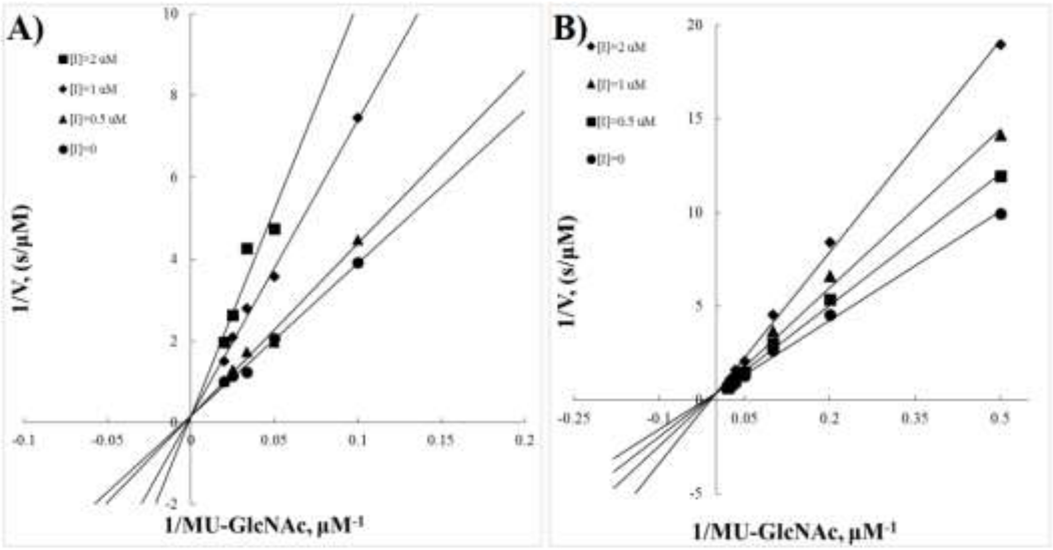

Fig. S1. A) Linewaver-Burk plots for inhibition of hsHexB by compound **15j**. B) Linewaver-Burk plots for inhibition of hOGA by compound **15b**

References

1. Floyd N, Vijayakrishnan B, Koeppe JR, et al. Thiyl glycosylation of olefinic proteins: S-linked glycoconjugate synthesis. Angew Chem Int Ed; 2009; 48, 7789-802.

2. Paul B, Korytnyk W. S-, N-, and O-glycosyl derivatives of 2-acetamido-2-deoxy-D-glucose with hydrophobic aglycons as potential chemotherapeutic agents and N-acetyl-beta-D-glucosaminidase inhibitors. Carbohydr Res; 1984; 126, 27-43.

3. André S, O'Sullivan S, Murphy PV, et al. Glycoclusters as lectin inhibitors: comparative analysis on two plant agglutinins with different folding as a step towards rules for selectivity. Tetrahedron; 2015; 71, 6867-80.

4. Landeyálvarez MA, Ochoaterán A, Pinaluis G, et al. Novel naphthalimide–aminobenzamide dyads as OFF/ON fluorescent supramolecular receptors in metal ion binding. Supramol Chem; 2016; 28, 892-906.

5. Ramchander J. Synthesis of 3(N(1,3dioxo 1H benzo[de]isoquinolin- 2(3H)-yl)alkyl)-2-(4-substituted) phenylthiazolidine-4- carboxylic Acid. Chem Sci Trans; 2016; 5, 163-70.
